# Prediction of immunogenicity of Rh antigens using *in silico* analysis of binding to human leukocyte antigen peptide, Basic/Translational Research

**DOI:** 10.1371/journal.pone.0334851

**Published:** 2025-10-27

**Authors:** Hee-Jeong Youk, Yong-Joon Cho, Yong-Hyun Han, Seong Who Kim, Dae-Hyun Ko

**Affiliations:** 1 Department of Laboratory Medicine, AMIST, Asan Medical Center, University of Ulsan College of Medicine, Seoul, Republic of Korea; 2 Department of Laboratory Medicine, Kangwon National University Hospital, Kangwon National University School of Medicine, Chuncheon, Republic of Korea; 3 Department of Molecular Bioscience, Kangwon National University, Chuncheon, Republic of Korea; 4 Laboratory of Pathology and Physiology, College of Pharmacy, Kangwon National University, Chuncheon, Republic of Korea; 5 Department of Biochemistry and Molecular Biology, Biomedical Research Center, University of Ulsan College of Medicine, Seoul, Republic of Korea; 6 Department of Laboratory Medicine, Asan Medical Center, University of Ulsan College of Medicine, Seoul, Republic of Korea; German Red Cross Blood Service, GERMANY

## Abstract

The association between human leukocyte antigen (HLA) types and blood group alloimmunization remains unclear. Previous studies have predominantly focused on predicting immunization events in cancer immunotherapy, but not blood group antigens. In this study, we investigated whether HLA peptide binding could predict the immunogenicity of blood group antigens. We performed *in silico* binding analysis of Rh antigens and representative HLA class II alleles using NetMHCpan-4.1 and NetMHCIIpan-4.1 algorithms. The distribution of strong binding regions (hotspots) differed across HLA loci and ethnic groups. In particular, the RhD and RhCE antigens showed several distinct hotspots for the HLA-DRB, -DQA-DQB, and -DPA-DPB HLA class II peptides. A hotspot of RHD*01W.1 in HLA-DRB had a substitution in p.Val270Gly. The number of hotspots and core amino acids was different for each HLA locus, and the amino acid regions (exofacial, transmembrane, and intracellular region) differed among the hotspots. Our findings underscore the significance of immunogenicity between the Rh antigens and HLA-DR, suggesting the potential clinical utility of predicting antibody development in blood transfusions. This *in silico* approach offers novel insights into understanding and managing alloimmunization events, particularly in patients with multiple alloantibodies when blood transfusion is required.

## Introduction

Human red blood cells (RBCs) contain over 366 erythrocyte antigens, which can be classified into various blood group systems. As of October 2024, the International Society of Blood Transfusion classifies blood types according to 47 blood group systems [1]. Among these, the ABO and RhD antigens have the highest clinical significance and immunogenicity. The Rh blood group system comprises numerous antigens located on variant forms of RhD and RhCE proteins [2]. Alloimmunization occurs when RBC antigens provoke the production of alloantibodies following exposure through transfusion, pregnancy, or transplantation [3]. In RBC alloimmunization, epitopes are antigen parts recognized by antibodies or T-cell receptors. Blood group antigens bind to human leukocyte antigen (HLA) molecules and are presented on antigen-presenting cells (APCs, such as macrophages and dendritic cells), triggering an immune response through T-cell receptors [4–6]. Two key epitope types are involved: B-cell epitopes, which are three-dimensional structures that bind antibodies, and T-cell epitopes, which are linear peptides presented by major histocompatibility complex (MHC) molecules on APCs. This dual recognition is essential for the immune response against transfused RBCs [4]. The risks of RBC alloimmunization increase based on disease type, ethnicity, HLA alleles, sex, and transfusion volume [7–11].

Pre-transfusion testing aims to prevent hemolytic transfusion reactions (HTRs) through ABO and RhD typing, antibody screening/identification, and crossmatching. Extended antigen-matched transfusions are used in diseases such as thalassemia and sickle cell disease. The clinical significance of the antibody against the corresponding antigen, as well as the immunogenicity of the corresponding antigen, should be considered when selecting a matched antigen for patients with HTR/hemolytic disease of the fetus and newborn severity. The clinical significance of several unexpected antibodies is well known; however, the underlying mechanisms responsible for the immunogenicity of blood group antigens remain unclear.

Giblett investigated the relative immunogenicity of blood group antigens for Caucasians and African Americans [12]. In this study, immunogenicity was estimated based on the distribution of antigens by ethnic group and the frequency of unexpected antibodies against the antigen. Tormey improved Giblett’s method [13,14]. In addition, Chung et al. showed the relative immunogenicity of blood group antigens in a Korean population [15].

In addition to antigen characteristics, immunogenicity can also be affected by the HLA background, which also greatly varies based on the ethnic group [16]. In particular, HLA class II variants at the DR locus are associated with RBC alloimmunity [11]. Recently, Trueba-Gómez et al. suggested that the antigenic variability among RhD variants contributes to their alloimmunization capacity [17]. RhD can be presented by a wide range of HLA molecules, making it highly immunogenic. Diego et al. identified the HLA class II haplotype DRB1 [18]. The HLA-DRB1 locus is polymorphic, and HLA-DR molecules account for >90% of the HLA class II expressed on APCs. Herein, we focused on HLA class II DR molecules.

HLA class II molecules may be the “gatekeepers” of CD4^+^ T-helper (T_H_) cell immunity [18–20]. Lessard et al. and McGill et al. have confirmed that novel DRB1 alleles are associated with factor VIII-neutralizing antibodies [21,22]. A case suggesting the involvement of HLA typing in alloimmunization events for Asian-type DEL was recently reported [23]. Similarly, other studies suggest an association between HLA typing and blood group alloimmunization events [6,24–26].

However, the association between HLA type and blood group alloimmunization events and the underlying mechanism have not been elucidated. Existing studies have attempted to predict immunization events triggered by the binding of HLA peptides to foreign antigen peptides [27]. These studies were primarily used to predict neoantigens in cancer immunotherapy but have not been applied to blood group antigens. Our study focused on T-cell epitopes rather than antibody formation by B cells. Thus, this study aimed to address the following question: can HLA peptide binding explain the immunogenicity of blood group antigens in RBC transfusions?

## Materials and methods

We selected the Rh blood group system for *in silico* analysis. This study used publicly available information and did not involve human studies or participants; therefore, no Institutional Review Board approval was sought.

### Blood group antigen selection

We analyzed the best-known Rh antigens based on the International Society of Blood Transfusion [1]. We selected *RHD* normal alleles (RHD*01, RHD*01.01), three weak D alleles (RHD*01.W.1, RHD*01.W.2, and RHD*01.W.3), known as RHD variants that do not affect immunogenicity, and the *RHCE* normal allele (RHCE*01). RHCE*01 was sequenced similarly to RhD but has significant differences in immunogenicity. RhD and RHCE reference sequences were used (Accession No. NM_016124.6 and NM_020485.8).

### Pilot study

We conducted a pilot study before the *in silico* analysis. Representative HLA alleles for each locus, HLA-A (A*01:01, A*02:01, A*03:01), HLA-B (B*07:02, B*08:01, B*27:05), HLA-C (C*01:02, C*02:02, C*03:01), HLA-DRB1 (DRB1*01:01, DRB1*03:01, DRB1*04:01), and HLA-DQ (DQA1*01:01-DQB1*02:01, DQA1*01:01-DQB1*03:01, DQA1*01:01-DQB1*04:01) were selected for the analysis.

### Selecting an *in silico* binding prediction algorithm

*In silico* binding analysis of major Rh antigens and HLA was performed using the NetMHCpan-4.1 and NetMHCIIpan-4.1 algorithms [28]. The servers provide binding prediction scores and %Rank values and classify peptides as strong binders (SBs) or weak binders (WBs), aiding in the identification of potential T-cell epitopes. We used as input the FASTA data (RhD and RHCE reference peptide sequences) and the HLA allele, with all other options set to default (9-mer peptides and <0.5% rank threshold for SBs). For MHC class II binding prediction, we used 9-mer peptides to represent the canonical binding core. Although MHC class II molecules can accommodate peptides of variable lengths, often ranging from 12 to 30 amino acids, the binding specificity is primarily determined by a 9–amino acid core sequence [28]. The 9-mer is the core of MHC II binding. However, both the length of the entire peptide and the position of the 9-mer core must be carefully considered when predicting MHC II binding. “Predicted peptide (core amino acid)” denotes the list of peptides that have been interrogated against the selected MHC molecules on this server.

### HLA allele selection

We analyzed the HLA class II peptides HLA-DRB, HLA-DQA-DPB, and HLA-DPA-DPB, focusing on HLA-DRB1 during the analysis of ethnic groups. HLA allele frequencies in the populations (USA Caucasian pop4, USA African American pop4, USA Hispanic pop2, and USA Asian pop2) were derived from the Allele Frequency Net Database (http://www.allelefrequencies.net/hla6006a.asp) [29].

### Statistical analysis

Peptide-MHC binding affinities were predicted using NetMHCpan-4.1 and NetMHCIIpan-4.1 on a Linux platform.

## Results

### Pilot study of Rh antigen binding to HLA class I

Six blood group alleles (RHD*01, RHD*01.01, RHD*01W.1, RHD*01W.2, RHD*01W.3, and RHCE*01) were subjected to *in silico* binding analysis to HLA-A, HLA-B, and HLA-C. Hotspots (defined as an epitope that binds to the receptor of a CD4^+^ T cell, or an RBC peptide that strongly binds to MHC class II) were observed in HLA-A (A*01:01, A*02:01, and A*03:01), HLA-B (B*07:02, B*08:01, and B*27:05), and HLA-C (C*01:02, C*02:02, and C*03:01; S1 Fig). However, they were not observed in the remaining HLA class I and Rh systems. Strong binding was scattered and did not show significant positions.

### Pilot study of Rh antigen binding to a part of HLA class II

The six blood group alleles were analyzed for binding to DRB1 and DQ. Hotspots were observed in HLA-DRB1 (DRB1*03:01 and DRB1*04:01), and HLA-DQ (DQA1*01:01-DQB1*03:01, and DQA1*01:01-DQB1*04:01; S2 Fig). Unlike the pilot study investigating the binding of the Rh blood group to HLA class I, the HLA class II pilot study showed a few clustered hotspots.

### Hotspot analysis of the Rh blood group system and HLA class II alleles: Consideration of the potential clinical impact of p.Val270Gly substitution in RHD*01W.1

**Fig 1:**
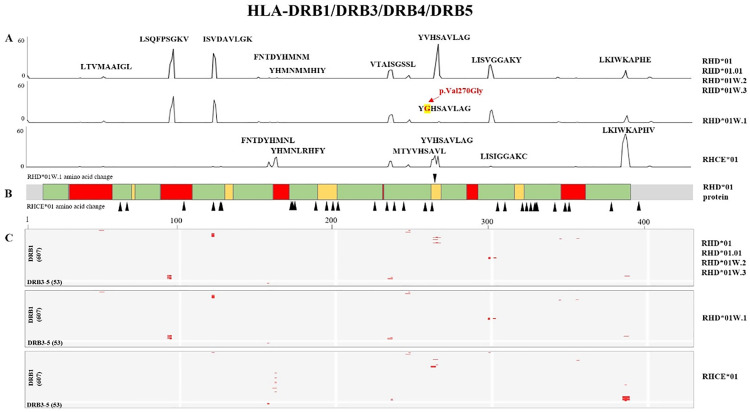
*In silico* binding analysis of *RHD* and HLA-DRB alleles. (**A**) The x-axis shows the position of the RH peptide sequence, and the y-axis shows the strong binding number of the HLA allele. RHD*01W.1 in HLA-DRB has a substitution of p.Val270Gly, unlike RHD*01W.2, RHD*01W.3, and RHD*01.01. The RhD and RhCE antigens differ in several strong binding regions for HLA-DR peptides. (**B**) Differences between RHD*01 and RHCE*01 amino acids are marked with black triangles. RHD*01 protein; green: transmembrane region, red: exofacial region, yellow: intracellular region. (**C**) Heatmap of the strong bonds of Rh antigens and 607 HLA-DRB1 and 53 HLA-DRB3–5. Red spots represent strong bonds (hotspots).

**Fig 2:**
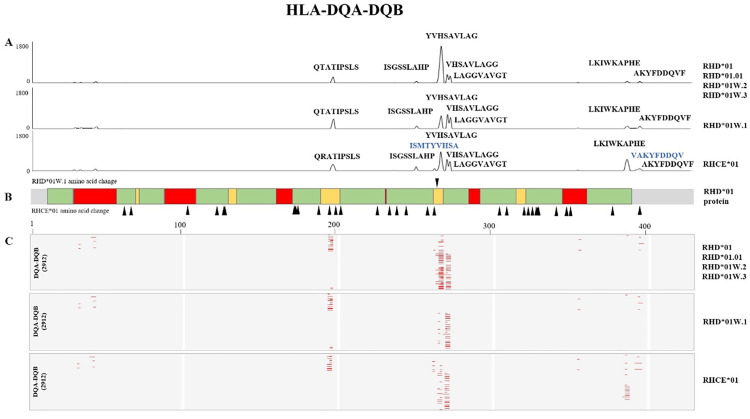
*In silico* binding analysis of *RHD* alleles and HLA-DQA-DQB. (**A**) The x-axis shows the position of the RH peptide sequence, and the y-axis shows the strong binding number of the HLA allele. The RhD and RhCE antigens differ in several strong binding regions for HLA-DQA-DQB peptides. (**B**) Differences between RHD*01 and RHCE*01 amino acids are marked with black triangles. RHD*01 protein; green: transmembrane region, red: exofacial region, yellow: intracellular region. (**C**) Heatmap of the strong bond of Rh antigens and 2912 HLA-DQA-DQB. Red spots represent strong bonds (hotspots).

**Fig 3:**
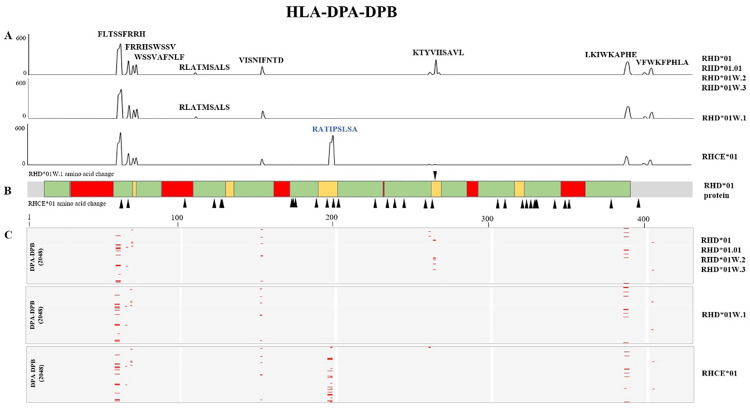
*In silico* binding analysis of *RHD* alleles and HLA-DPA-DPB. (**A**) The x-axis shows the position of the RH peptide sequence, and the y-axis shows the strong binding number of the HLA allele. The RhD and RhCE antigens differ in several strong binding regions for HLA-DPA-DPB peptides. (**B**) Differences between RHD*01 and RHCE*01 amino acids are marked with black triangles. RHD*01 protein; green: transmembrane region, red: exofacial region, yellow: intracellular region. (**C**) Heatmap of the strong bonds of Rh antigens and 2048 HLA-DPA-DPB. Red spots represent strong bonds (hotspots).

We performed *in silico* analysis on HLA class II alleles (HighQ-DRB, HighQ-DP, HighQ-DQ, DRB1, DRB3, DRB4, DRB5, DP, and DQ) and the six blood group alleles. The HLA class II alleles are listed in S1 Table. Results of the *in silico* analysis are shown in Figs 1–3. The analysis of the hotspots of the Rh blood group system, HLA-DRB1, DP, and DQ are shown in each figure. Although the genes encoding the Rh proteins (RhD and RhCE) are highly homologous, their immunogenicity differs [2]. In our study, RhD and RhCE antigens showed different strong binding regions for HLA-DRB1, DP, and DQ peptides (Figs 1–3).

**Fig 4:**
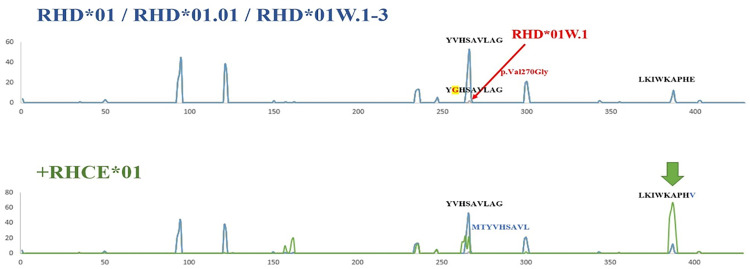
A merged line graph of the bond between *RHD* and HLA-DRB alleles. Blue: the x-axis shows Rh peptide sequences (RHD*01, RHD*01.01, RHD*01W.2, and RHD*01W.3), and the y-axis shows the number of HLA-DRB allele strong binding. Black: the x-axis shows weak D 1 peptide sequences (RHD*01W.1), and the y-axis shows the number of HLA-DRB alleles with strong bonds. Green: the x-axis shows the RHCE peptide sequences (RHCE*01), and the y-axis shows the number of HLA-DRB alleles with strong bonds.

RHD*01, RHD*01.01, RHD*01W.2, RHD*01W.3, and HLA-DR groups (HLA-DRB1, HLA-DRB3, HLA-DRB4, and HLA-DRB5) had the same hotspots, except for the RHD*01W.1. The most common type of weak D was the weak D type 1 (RHD*01W.1), which is designated as Val270Gly [23]. RHD*01W.1 also showed a hotspot on p.Val270Gly, unlike RHD*01, RHD*01.01, RHD*01W.2, and RHD*01W.3 in HLA-DRB1, -DRB3, -DRB4, and DRB5 (Figs 1 and 4). Therefore, we wondered if the 270th amino acid substitution in RHD*01W.1 could cause a clinical difference. Weak D 1–3 have similar immunogenicity to normal RhD and can safely transfuse RhD-positive blood in Caucasians [30]. Weak D 1, 2, 3, 4.0, 4.1, and 5 can be considered Rh-positive and transfuse with Rh-positive RBCs. In contrast, weak D 4.2–11 and 15 are considered Rh-negative and transfuse with Rh-negative RBCs [31–34]. The DEL phenotype of D variants, which is particularly prevalent in East Asia, expresses extremely low levels of the D antigen and has generally been regarded as non-immunogenic. However, anti-D has recently been detected against Asian-type DEL [23]. The *RHD* (c.1227G > A) allele variant, an Asian-type DEL, has not previously been reported to induce anti-D immunity [35,36]. HLA typing revealed alleles that were previously associated with anti-D immunity (HLA-DRB1*15:01 and HLA-DQB1*02:02). Therefore, unlike weak D types 2 and 3, weak D type 1 may also exhibit a capacity for anti-D production under certain immunogenic conditions.

### Predicted core amino acids (hotspot regions) of Rh antigens that bind to HLA class II peptides

The locations of predicted core amino acids (hotspot regions) in the Rh antigens with HLA class II are shown in Table 1. Based on the comparison of these five RHD alleles, we investigated whether the position of each hotspot was an intracellular, transmembrane, or exofacial region. The sequences of the exofacial region of the Rh blood group comprise amino acids 32–53, 94–107, 158–167, 230–231, 282–290, and 347–358 [33,34,37,38]. Stott et al. mapped alloreactive T cell epitopes on the RhD protein by stimulating peripheral blood mononuclear cells from RhD-negative individuals with a panel of 68 overlapping synthetic 15-mer peptides spanning the complete RhD protein sequence [39]. Peptides 6 (amino acid 52–66, transmembrane), 13 (amino acid 97–111, exofacial), 17 (amino acid 117–131, transmembrane), and 28 (amino acid 177–191, transmembrane and intracellular) were recognized by T cells from more than 50% of the alloimmune donors [39].

**Table 1:** Rh blood group systems and HLA class II hotspot regions.

	HLA-DRB			HLA-DQA-DPB			HLA-DPA-DPB		
RH types	Amino acid start position	Core amino acid	Region	Amino acid start position	Core amino acid	Region	Amino acid start position	Core amino acid	Region
RHD*01 RHD*01.01 RHD*01W.2 RHD*01W.3	38	LEDQKGLVA	exofacial	31	FTHYDASLE	transmembrane	64	FLTSSFRRH	transmembrane
	54	LTVMAAIGL	transmembrane	35	DASLEDQKG	exofacial	69	FRRHSWSSV	transmembrane
	98	LSQFPSGKV	exofacial	45	VASYQVGQD	exofacial	74	WSSVAFNLF	intracellular
	125	ISVDAVLGK	transmembrane	200	QTATIPSLS	transmembrane	114	RLATMSALS	transmembrane
	153	LRMVISNIF	transmembrane	239	FNTYYAVAV	transmembrane	156	VISNIFNTD	transmembrane
	161	FNTDYHMNM	exofacial	253	ISGSSLAHP	transmembrane	264	KISKTYVHS	intracellular
	165	YHMNMMHIY	exofacial	265	ISKTYVHSA	intracellular	267	KTYVHSAVL	transmembrane
	239	FNTYYAVAV	transmembrane	269	YVHSAVLAG	transmembrane	269	YVHSAVLAG	transmembrane
	250	VTAISGSSL	transmembrane	270	VHSAVLAGG	transmembrane	391	KIWKAPHEA	intracellular
	269	YVHSAVLAG	transmembrane	275	LAGGVAVGT	transmembrane	402	FDDQVFWKF	intracellular
	303	LISVGGAKY	transmembrane	358	MIGFQVLLS	exofacial	406	VFWKFPHLA	intracellular
	347	LVLDTVGAG	exofacial	390	LKIWKAPHE	transmembrane	407	FWKFPHLAV	intracellular
	358	MIGFQVLLS	exofacial	399	AKYFDDQVF	intracellular			
	390	LKIWKAPHE	intracellular						
	407	FWKFPHLAV	intracellular						
RHD*01W.1	38	LEDQKGLVA	exofacial	31	FTHYDASLE	transmembrane	64	FLTSSFRRH	transmembrane
	54	LTVMAAIGL	transmembrane	35	DASLEDQKG	exofacial	69	FRRHSWSSV	transmembrane
	98	LSQFPSGKV	exofacial	45	VASYQVGQD	exofacial	74	WSSVAFNLF	intracellular
	125	ISVDAVLGK	transmembrane	200	QTATIPSLS	transmembrane	114	RLATMSALS	transmembrane
	153	LRMVISNIF	transmembrane	239	FNTYYAVAV	transmembrane	156	VISNIFNTD	transmembrane
	161	FNTDYHMNM	exofacial	253	ISGSSLAHP	transmembrane	391	KIWKAPHEA	intracellular
	165	YHMNMMHIY	exofacial	265	ISKTYVHSA	intracellular	402	FDDQVFWKF	intracellular
	239	FNTYYAVAV	transmembrane	269	YVHSAVLAG	transmembrane	406	VFWKFPHLA	intracellular
	250	VTAISGSSL	transmembrane	270	VHSAVLAGG	transmembrane	407	FWKFPHLAV	intracellular
	269	YGHSAVLAG	transmembrane	275	LAGGVAVGT	transmembrane			
	303	LISVGGAKY	transmembrane	358	MIGFQVLLS	exofacial			
	347	LVLDTVGAG	exofacial	390	LKIWKAPHE	transmembrane			
	358	MIGFQVLLS	exofacial	399	AKYFDDQVF	intracellular			
	390	LKIWKAPHE	intracellular						
	407	FWKFPHLAV	intracellular						
RHCE*01	38	LEDQKGLVA	exofacial	31	FTHYDASLE	transmembrane	64	FLTSSFRRH	transmembrane
	54	LTVMAALGL	transmembrane	35	DASLEDQKG	exofacial	69	FRRHSWSSV	transmembrane
	62	LGFLTSNFR	transmembrane	45	VASYQVGQD	exofacial	74	WSSVAFNLF	intracellular
	124	LISAGAVLG	transmembrane	200	QRATIPSLS	transmembrane	156	VISNIFNTD	transmembrane
	130	VLGKVNLAQ	transmembrane	239	FNTYYALAV	transmembrane	160	IFNTDYHMN	exofacial
	161	FNTDYHMNL	exofacial	253	ISGSSLAHP	transmembrane	201	RATIPSLSA	transmembrane
	165	YHMNLRHFY	exofacial	265	ISMTYVHSA	intracellular	263	RKISMTYVH	transmembrane
	239	FNTYYALAV	transmembrane	269	YVHSAVLAG	transmembrane	391	KIWKAPHVA	intracellular
	250	VTAISGSSL	transmembrane	270	VHSAVLAGG	transmembrane	402	FDDQVFWKF	intracellular
	267	MTYVHSAVL	transmembrane	275	LAGGVAVGT	transmembrane	406	VFWKFPHLA	intracellular
	269	YVHSAVLAG	transmembrane	358	MIGFQVLLS	exofacial	407	FWKFPHLAV	intracellular
	303	LISIGGAKC	transmembrane	390	LKIWKAPHE	transmembrane			
	358	MIGFQVLLS	exofacial	391	KIWKAPHVA	intracellular			
	390	LKIWKAPHV	transmembrane	398	VAKYFDDQV	intracellular			
	407	FWKFPHLAV	intracellular	399	AKYFDDQVF	intracellular			

*Note*: “Core amino acid” refers to the list of peptides that have been interrogated against the selected MHC molecules. Red letters indicate important amino acids. These represent a substitution in the protein at position 270, where the amino acid valine (Val) is replaced by glycine (Gly).

In our *in silico* analysis, hotspots were located in the intracellular, transmembrane, and exofacial regions depending on HLA-DRB, -DQA-DQB, and -DPA-DPB. In HLA-DRB, each RhD antigen had the same core amino acids (epitope) as RHD*01, RHD*01.01, RHD*01W.2, and RHD*01W.2, except for RHD*01W.1. Furthermore, RHD*01W.1 had a substitution in p.Val270Gly, located in the transmembrane region, unlike RHD*01W.2 and RHD*01W.3.

Among the RhD antigens, the start positions of the hotspots, number, and location of membrane regions differed between HLA-DRB, -DQA-DQB, and -DPA-DPB. The different amino acid start positions unique to the RhCE antigen were the 62^nd^, 124^th^, 130^th^, and 267^th^, which were not present in RhD antigens. RhD and RhCE antigens showed 15 hotspots in HLA-DRB, whereas RhD antigens showed 13 in HLA-DQA-DPB. RhCE in HLA-DQA-DPB had similar hotspots to the RhD antigens. Additionally, the hotspots of HLA-DPA-DPB and RhD antigens were not all on the exofacial region. Most hotspots in HLA-DPA-DPB were located in the transmembrane and intracellular area. Moreover, the number of hotspots and core amino acids were different for each HLA locus, and the regions (exofacial, transmembrane, and intracellular region) of the amino acids in the hotspots differed.

### Analysis of HLA-DRB1 hotspot regions among the four ethnic groups

We investigated the frequency and hotspots of HLA-DRB1 in four ethnic groups (Caucasian, African American, Hispanic, and Asian) using the population frequencies of the HLA alleles from the Allele Frequency Net Database [29]. The amino acid position and sequence of hotspots in HLA-DRB1 and Rh antigens (RHD*01.01, RHD*01W.1, RHD*01W.2, RHD*01W3, and RHCE*01) of the four ethnic groups are listed in Table 2. RHD*01.01, RHD*01W.2, and RHD*01W3 had different hotspots with DRB1*04:01, DRB1*04:14, DRB1*04:17, DRB1*08:02, DRB1*08:09, DRB1*09:01, and DRB1*10:01 compared to RHD*01W.1. RhD and RhCE antigens showed significant differences in hotspots across the four ethnic groups.

**Table 2:** HLA-DRB1 hotspot (SB) positions and the frequency of HLA-DRB1 in the four ethnic groups.

HLA-DRB1 Allele	Caucasian (n = 1070)	African American (n = 2411)	Hispanic (n = 1999)	Asian (n = 1772)	RHD*01.01/ RHD*01W.2/ RHD*01W.3		RHD*01W.1		RHCE*01	
					Amino acid start position of the hotspot	Core amino acids	Amino acid start position of the hotspot	Core amino acids	Amino acid start position of the hotspot	Core amino acids
01:02	0.0202	0.0399	0.033	0.0003	250	VTAISGSSL	250	VTAISGSSL	250	VTAISGSSL
01:03	0.0192	0.0023	0.0063	0.0003	–	–	–	–	390	LKIWKAPHV
**03:01**	**0.1039**	0.0707	0.0733	0.0537	125	ISVDAVLGK	125	ISVDAVLGK	–	–
03:05	0.001	0.0002	0.0003	0	–	–	–	–	161	FNTDYHMNL
03:06		0.0002	0	0	125	ISVDAVLGK	125	ISVDAVLGK	–	–
**04:01**	**0.1039**	0.0229	0.0145	0.0091	269	YVHSAVLAG	–	–	269	YVHSAVLAG
04:02	0.0039	0.0004	0.0195	0.0037	390	LKIWKAPHE	390	LKIWKAPHE	130	VLGKVNLAQ
04:07	0.0154	0.004	0.0643	0.0014	269	YVHSAVLAG	269	YGHSAVLAG	269	YVHSAVLAG
04:08	0.0058	0.0006	0.0035	0.0014	390	YVHSAVLAG	269	YVHSAVLAG	269	YVHSAVLAG
04:14		0	0.0003	0	269	YVHSAVLAG	–	–	–	–
04:17		0	0.0005	0	269	YVHSAVLAG	–	–	–	–
04:18		0	0.0003	0	358	MIGFQVLLS	358	MIGFQVLLS	358	MIGFQVLLS
**07:01**	**0.126**	0.0977	**0.1046**	0.082	–	–	–	–	267	MTYVHSAVL
07:03		0	0	0.0003	–	–	–	–	267	MTYVHSAVL
08:02	0.0029	0.001	0.0733	0.013	269	YVHSAVLAG	–	–	–	–
08:09		0	0	0.0023	269	YVHSAVLAG	–	–	–	–
**09:01**	0.0106	0.0316	0.0095	**0.1018**	269	YVHSAVLAG	–	–	–	–
10:01	0.0077	0.0185	0.0145	0.0311	269	YVHSAVLAG	–	–	–	–
12:01	0.0173	0.0395	0.012	0.0289	303	LISVGGAKY	303	LISVGGAKY	–	–
12:02		0.0027	0.0008	0.0741	303	LISVGGAKY	303	LISVGGAKY	–	–
12:08		0	0	0.0003	303	LISVGGAKY	303	LISVGGAKY	–	–
13:02	0.0519	0.0645	0.0388	0.0362	–	–	–	–	165	YHMNLRHFY
13:16		0.0004	0	0	–	–	–	–	165	YHMNLRHFY
13:31		0.0006	0	0	–	–	–	–	165	YHMNLRHFY
13:36		0.0002	0	0	–	–	–	–	165	YHMNLRHFY
**15:01**	**0.1394**	0.0293	0.0668	0.0792	98	LSQFPSGKV	98	LSQFPSGKV	390	LKIWKAPHV
15:02	0.0077	0.0017	0.0133	0.0809	98, 239	LSQFPSGKV, FNTYYAVAV	98, 239	LSQFPSGKV, FNTYYAVAV	239, 390	FNTYYALAV, LKIWKAPHV
**15:03**	0.001	**0.1175**	0.0113	0.0006	98	LSQFPSGKV	98	LSQFPSGKV	390	LKIWKAPHV
15:04		0	0.0003	0.0006	98, 390	LSQFPSGKV, LKIWKAPHE	98, 390	LSQFPSGKV, LKIWKAPHE	390	LKIWKAPHV
15:06		0	0	0.004	98	LSQFPSGKV	98	LSQFPSGKV	390	LKIWKAPHV
15:07		0	0	0.0003	98	LSQFPSGKV	98	LSQFPSGKV	390	LKIWKAPHV
15:10	0.001				98, 390	LSQFPSGKV, LKIWKAPHE	98, 390	LSQFPSGKV, LKIWKAPHE	390	LKIWKAPHV
15:11	0.001				239	FNTYYAVAV	239	FNTYYAVAV	390	LKIWKAPHV
15:14		0	0	0.0003	98, 239	LSQFPSGKV, FNTYYAVAV	98, 239	LSQFPSGKV, FNTYYAVAV	239, 390	FNTYYALAV, LKIWKAPHV

*Note*: Allele Frequency: Total number of copies of the allele in the population sample (Alleles/2n) in decimal format. HLA alleles with frequency ≥10% among the four ethnic groups are shown in bold.

HLA-DRB1*03:01, 04:01, 07:01, and 15:01 had HLA allele frequencies >10% in Caucasians. In Caucasians, HLA-DRB1*15:01 had the highest frequency and showed a hotspot at the 98^th^ position (LSQFPSGKV) in the RhD antigen and a hotspot at the 390^th^ position (LKIWKAPHV) in the RhCE antigen. HLA-DRB1*15:01 had hotspots at the exofacial region in RhD antigens and intracellular region in RhCE. Furthermore, all alleles except HLA-DRB1*15:01 had hotspots at the transmembrane region in RhD or RhCE peptides. HLA-DRB1*15:03 had HLA allele frequencies >10% in African Americans. Additionally, hotspots were observed at the exofacial and intracellular areas in RhD and RhCE peptides, respectively. HLA-DRB1*09:01 had HLA allele frequencies >10% in Asians, and its hotspots were located in the transmembrane region in RhD antigens, except for RHD*01W.1. Here, we only presented 34 HLA-DRB1 segments with hotspots in the four ethnic groups. The total data of HLA-DRB1 hotspot (SB) position and the frequency of HLA-DRB1 in the four ethnic groups are provided in S2 Table.

### HLA-DRB1 hotspot regions among Korean and Japanese populations

We investigated the frequency and hotspots of HLA-DRB1 in Korean and Japanese populations. Here, RHD*01W.1 had different hotspot positions of the HLA-DRB1 allele: DRB1*04:01, DRB1*08:02, DRB1*09:01, and DRB1*10:01. The amino acids in the hotspot of RhD antigens were as follows: 125^th^ ISVDAVLGK, 269^th^ YVHSAVLAG, 303rd LISVGGAKY, 98^th^ LSQFPSGKV, 239^th^ FNTYYAVAV, and 390^th^ LKIWKAPHE. The HLA-DRB1 allele frequency and hotspot position in Korean and Japanese populations are shown in S3 Table.

## Discussion

**Fig 5:**
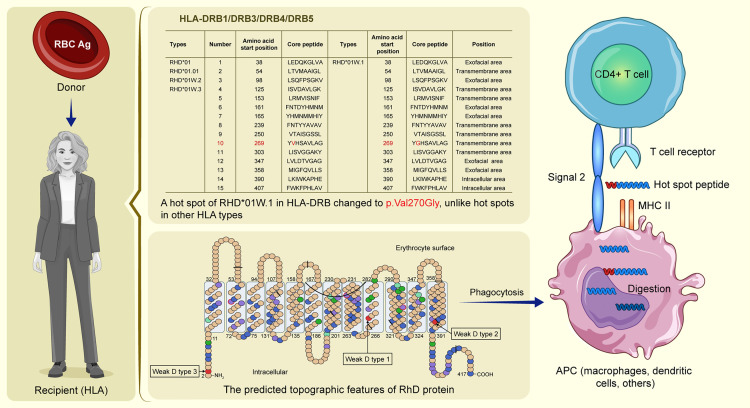
Mechanism of red blood cell (RBC) alloimmunization. The first step in RBC alloimmunization involves antigen-presenting cells (APCs) that phagocytose transfused RBCs, process their antigens, and present these antigens as peptides on major histocompatibility complex (MHC) II molecules to CD4^+^ T cells. In the second step, these activated CD4^+^ T cells provide the necessary help to B cells, which ultimately leads to the production of antibodies against the alloantigens on RBCs. During these two processes, we focus on peptides on MHC II molecules presented to CD4^+^ T cells. The left panel shows a blood transfusion scenario where the donor’s RBCs express RhD antigens introduced into the recipient with specific HLA types. The central table details the different HLA-DRB alleles (HLA-DRB1/DRB3/DRB4/DRB5) and their amino acid sequences, with a specific mutation at p.Val270Gly in RHD*01W.1, which distinguishes it from other RhD variants (RHD*01 to RHD*01W.3). It shows the amino acid start positions, core peptides, and locations (exofacial, transmembrane, or intracellular), highlighting immunogenic hotspots. The central figure below shows the predicted topographic features of the RhD protein on the erythrocyte surface. Molecularly defined weak D types are highlighted: weak D types 1, 2, and 3 (in red) as originally described by Wagner et al. [34,35]. Following phagocytosis by APCs such as macrophages and dendritic cells, RBCs are digested, and antigenic peptides are presented on MHC class II molecules on the surface of the APCs. The interaction between the antigen-MHC II complex and T cell receptor on CD4^+^ T cells provides Signal 1 for T cell activation. Additional co-stimulatory signals (Signal 2) are required for the complete activation of CD4^+^ T cells. Activated T cells initiate an immune response against foreign RBC antigens, leading to alloimmunization.

In this study, we performed *in silico* binding analysis of major Rh antigens and HLA loci. Our findings are summarized in Fig 5, which illustrates the process of alloimmunization against RBC antigens in a recipient. When donor RBC antigens are introduced into the recipient’s body, the recipient’s HLA recognizes foreign antigens. Antigen processing and presentation by APCs are as follows: APCs engulf, digest, and present RhD peptides on MHC class II molecules to CD4^+^ T cells. The interaction between APCs and CD4^+^ T cells is crucial for immune response initiation, highlighted by the recognition of the “hotspot” peptide, leading to potential alloimmunization if the recipient recognizes donor RhD antigens as foreign.

RhD and RhCE antigens had several strong binding regions against the HLA class II peptides. Additionally, we found that RHD*01W.1 in HLA-DRB had a p.Val270Gly substitution, unlike RHD*01W.2, RHD*01W.3, and RHD*01.01.

The analysis of the Rh blood group system revealed evenly distributed hotspots across the exofacial, transmembrane, and intracellular regions. Moreover, RhD and RhCE antigens showed different binding profiles against HLA-DRB. The unique positions in RhCE antigens indicate variations that may impact the alloimmunization of RhCE antigens, compared to the RhD antigens.

The hotspot distribution differed across each HLA locus-based ethnic group. In Caucasians, HLA-DRB1*15:01 had hotspots at the exofacial region in RhD antigens or intracellular regions in RhCE peptides. HLA-DRB1*04:01 had hotspots at the transmembrane region in RhD or RhCE peptides and a hotspot on p.Val270Gly, which altered the 270^th^ amino acid in RHD*01W.1. HLA-DRB1*04, HLA-DRB1*10, and HLA-DRB1*15 have been investigated in patients with post-transfusion alloimmunization [40], and so have HLA-DRB1*04 and DRB1*15 in Fy^a^-immunized patients following blood transfusion [41]. HLA-DRB1*15:01 is a major factor that regulates the response of human helper T cells to RhD proteins [6,41,42]. Additionally, HLA-DRB1*01:01, DRB1*01:02, and DRB1*10:01 are overrepresented in Jk^a^-immunized patients [43]. Although we only analyzed RhD and RhCE antigens in this study, we identified a hotspot at DRB1*15:01 (98^th^ LSQFPSGKV; exofacial region, 390^th^ LKIWKAPHV; intracellular region). RhD sequences that stimulate T cells from allogeneic immune donors reside in the exofacial loop as well as in the transmembrane and intracellular region [39]. HLA-DRB1*15:01 had hotspots at the exofacial region in RhD antigens. In our study, this hotspot (98th LSQFPSGKV) was a peptide of the RhD protein that stimulates T cells (amino acids 97–111). Therefore, we wondered if the absence of hotspots in HLA class II would decrease the likelihood of alloimmunization after transfusion.

Bioinformatics methods have been used to predict immunogenicity, focusing predominantly on the immuno-oncological relevance of HLA class I. Our study focused on the importance of immunogenicity between the Rh antigens and HLA-DR of HLA class II. This process is based on humoral immunity, and it may be relevant to analyze the binding affinity of HLA class II to RBC peptides (epitopes) in APCs. Thus, programs that predict HLA peptide binding can be used when novel variants of blood groups appear.

A major limitation of this study is the absence of wet-lab experiments to validate the *in silico* findings. However, our results may be clinically significant because in silico analysis showed hotspot (SB) regions between HLA loci and Rh antigens. Future studies are required to confirm whether HLA class II and Rh antigens bind to hotspots. *In silico* binding analysis between major blood group antigens and HLA peptides may facilitate the assessment of the risks for alloimmunogenic reaction and predicting its prevention by prioritizing the corresponding antigen-positive blood in RhD and other blood groups during a blood transfusion. By predicting how different Rh alleles interact with HLA class II molecules, this approach can significantly enhance transfusion compatibility, inform personalized medicine strategies.

To the best of our knowledge, this *in silico* analysis of binding to the HLA peptide is the first study to use bioinformatics to predict the immunogenicity of Rh blood group antigens with HLA class II, expanding the scope beyond the traditional focus on HLA class I.

## Supporting information

S1 Fig Schematic diagram of the binding hotspots between the Rh antigens and HLA class I. The x-axis represents the amino acid position of each HLA class I. The y-axis represents the number of antigen peptides of the six blood types (RHD*01, RHD*01.01 (normal RhD), RHD*01W.1, RHD*01W.2, RHD*01W.3, and RHCE*01) with strong bonds at that position.
(DOCX)

S2 Fig *In silico* binding analysis between the Rh blood group antigens and HLA class II peptides. The x-axis represents the amino acid position of each HLA class II. The y-axis represents the number of antigen peptides of the six blood types (RHD*01, RHD*01.01 (normal RhD), RHD*01W.1, RHD*01W.2, RHD*01W.3, and RHCE*01) with strong bonds at that position.
(DOCX)

S1 Table HLA class II alleles (660 HLA-DRB, 2048 HLA-DPA-DPB, and 2912 HLA-DQA-DQB alleles) used in the *in silico* analysis. (DOCX)

S2 Table HLA-DRB1 hotspot (SB) regions and frequencies in the four ethnic groups. The serology of HLA-DRB1 in RhD antigens showed that DR1 had VTAISGSSL at the 250^th^ position of the hotspot. In contrast, DR17 (DR3) had ISVDAVLGK at the 125^th^ position of the hotspot. DR4, DR7, DR8, and DR10 had a high frequency of YVHSAVLAG core amino acids at the 269^th^ amino acid start position of the hotspot. Furthermore, DR12 and DR15 had high frequencies of LISVGGAKY and LSQFPSGKV core amino acids at the 303^rd^ and 98^th^ positions of the hotspot, respectively. RhCE in HLA-DRB1 showed a different hotspot start position in the RhD antigens.
(DOCX)

S3 Table HLA-DRB1 gene frequencies and Rh hotspot positions in Korean and Japanese populations. (DOCX)
