# Supplementary material for: Prediction of immunogenicity of Rh antigens using in silico analysis of binding to human leukocyte antigen peptide, Basic/Translational Research
Source: PLoS One. 2025 Oct 27;20(10):e0334851. doi: 10.1371/journal.pone.0334851 (PMC12558515; doi:10.1371/journal.pone.0334851)
Supplement: S2 Fig — The x-axis represents the amino acid position of each HLA class II. The y-axis represents the number of antigen peptides of the six blood types (RHD*01, RHD*01.01 (normal RhD), RHD*01W.1, RHD*01W.2, RHD*01W.3, and RHCE*01) with strong bonds at that position. (DOCX) [file pone.0334851.s002.docx]

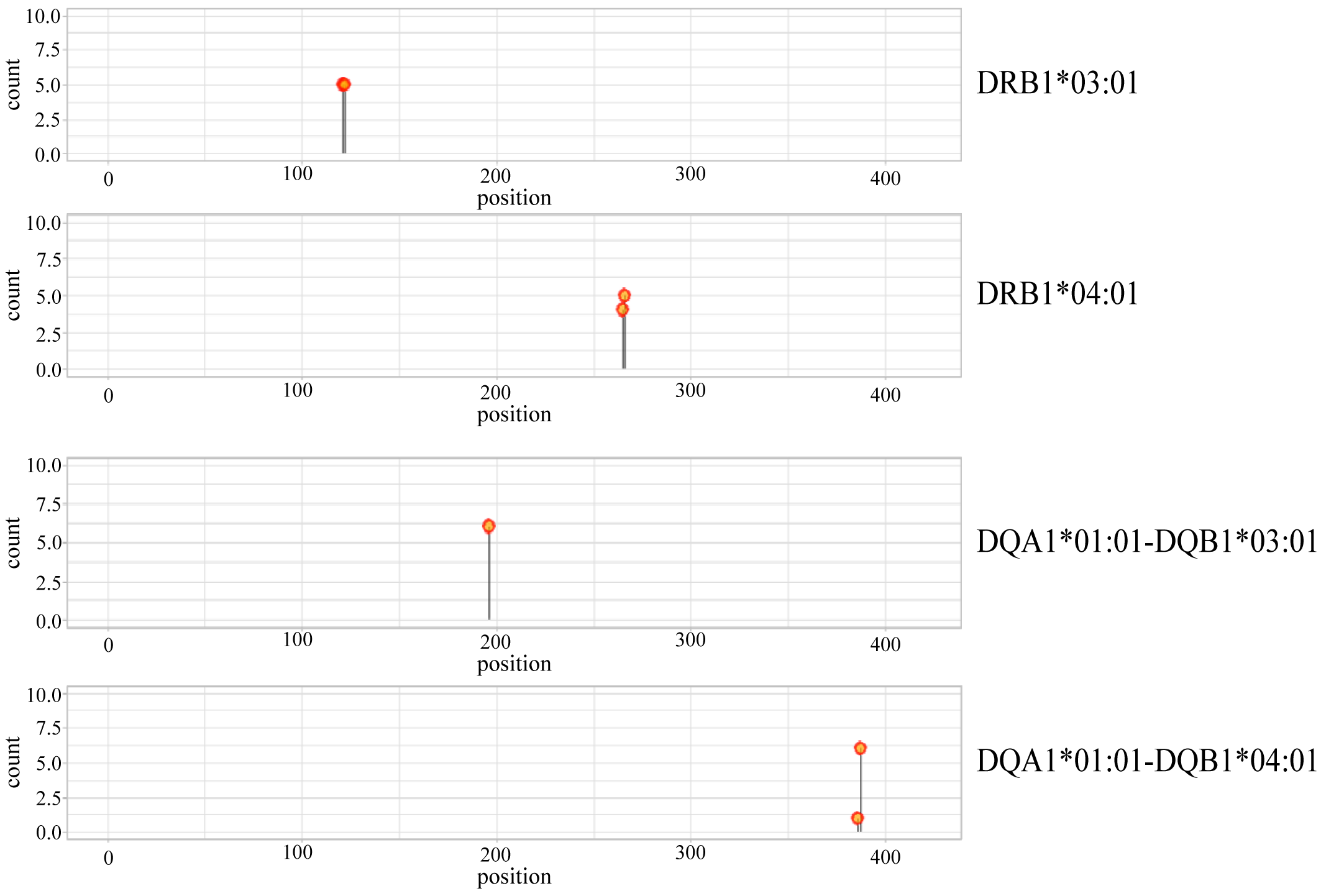


**S2 Fig. *In silico* binding analysis between the Rh blood group antigens and HLA class II peptides.** The x-axis represents the amino acid position of each HLA class II. The y-axis represents the number of antigen peptides of the six blood types (*RHD*01*, *RHD*01.01* (normal RhD), *RHD*01W.1*, *RHD*01W.2*, *RHD*01W.3*, and *RHCE*01*) with strong bonds at that position.
