# Supplementary material for: Prediction of immunogenicity of Rh antigens using in silico analysis of binding to human leukocyte antigen peptide, Basic/Translational Research
Source: PLoS One. 2025 Oct 27;20(10):e0334851. doi: 10.1371/journal.pone.0334851 (PMC12558515; doi:10.1371/journal.pone.0334851)
Supplement: S1 Table — (DOCX) [file pone.0334851.s003.docx]

**S1 Table. HLA class II alleles (660 HLA-DRB, 2048 HLA-DPA-DPB, and 2912 HLA-DQA-DQB alleles) used in the *in silico* analysis.**

| No. | HLA-DRB | HLA-DP | HLA-DQ |
| --- | --- | --- | --- |
| 1 | DRB1*0101 | HLA-DPA1*0103-DPB1*0101 | HLA-DQA1*0101-DQB1*0201 |
| 2 | DRB1*0102 | HLA-DPA1*0103-DPB1*0201 | HLA-DQA1*0101-DQB1*0202 |
| 3 | DRB1*0103 | HLA-DPA1*0103-DPB1*0202 | HLA-DQA1*0101-DQB1*0203 |
| 4 | DRB1*0104 | HLA-DPA1*0103-DPB1*0301 | HLA-DQA1*0101-DQB1*0204 |
| 5 | DRB1*0105 | HLA-DPA1*0103-DPB1*0401 | HLA-DQA1*0101-DQB1*0205 |
| 6 | DRB1*0106 | HLA-DPA1*0103-DPB1*0402 | HLA-DQA1*0101-DQB1*0206 |
| 7 | DRB1*0107 | HLA-DPA1*0103-DPB1*0501 | HLA-DQA1*0101-DQB1*0301 |
| 8 | DRB1*0108 | HLA-DPA1*0103-DPB1*0601 | HLA-DQA1*0101-DQB1*0302 |
| 9 | DRB1*0109 | HLA-DPA1*0103-DPB1*0801 | HLA-DQA1*0101-DQB1*0303 |
| 10 | DRB1*0110 | HLA-DPA1*0103-DPB1*0901 | HLA-DQA1*0101-DQB1*0304 |
| 11 | DRB1*0111 | HLA-DPA1*0103-DPB1*0001 | HLA-DQA1*0101-DQB1*0305 |
| 12 | DRB1*0112 | HLA-DPA1*0103-DPB1*1001 | HLA-DQA1*0101-DQB1*0306 |
| 13 | DRB1*0113 | HLA-DPA1*0103-DPB1*0101 | HLA-DQA1*0101-DQB1*0307 |
| 14 | DRB1*0114 | HLA-DPA1*0103-DPB1*0201 | HLA-DQA1*0101-DQB1*0308 |
| 15 | DRB1*0115 | HLA-DPA1*0103-DPB1*0301 | HLA-DQA1*0101-DQB1*0309 |
| 16 | DRB1*0116 | HLA-DPA1*0103-DPB1*0401 | HLA-DQA1*0101-DQB1*0310 |
| 17 | DRB1*0117 | HLA-DPA1*0103-DPB1*0501 | HLA-DQA1*0101-DQB1*0311 |
| 18 | DRB1*0118 | HLA-DPA1*0103-DPB1*0601 | HLA-DQA1*0101-DQB1*0312 |
| 19 | DRB1*0119 | HLA-DPA1*0103-DPB1*0701 | HLA-DQA1*0101-DQB1*0313 |
| 20 | DRB1*0120 | HLA-DPA1*0103-DPB1*0801 | HLA-DQA1*0101-DQB1*0314 |
| 21 | DRB1*0121 | HLA-DPA1*0103-DPB1*0901 | HLA-DQA1*0101-DQB1*0315 |
| 22 | DRB1*0122 | HLA-DPA1*0103-DPB1*1001 | HLA-DQA1*0101-DQB1*0316 |
| 23 | DRB1*0123 | HLA-DPA1*0103-DPB1*1101 | HLA-DQA1*0101-DQB1*0317 |
| 24 | DRB1*0124 | HLA-DPA1*0103-DPB1*1101 | HLA-DQA1*0101-DQB1*0318 |
| 25 | DRB1*0125 | HLA-DPA1*0103-DPB1*1201 | HLA-DQA1*0101-DQB1*0319 |
| 26 | DRB1*0126 | HLA-DPA1*0103-DPB1*1301 | HLA-DQA1*0101-DQB1*0320 |
| 27 | DRB1*0127 | HLA-DPA1*0103-DPB1*1401 | HLA-DQA1*0101-DQB1*0321 |
| 28 | DRB1*0128 | HLA-DPA1*0103-DPB1*1501 | HLA-DQA1*0101-DQB1*0322 |
| 29 | DRB1*0129 | HLA-DPA1*0103-DPB1*1601 | HLA-DQA1*0101-DQB1*0323 |
| 30 | DRB1*0130 | HLA-DPA1*0103-DPB1*1701 | HLA-DQA1*0101-DQB1*0324 |
| 31 | DRB1*0131 | HLA-DPA1*0103-DPB1*1801 | HLA-DQA1*0101-DQB1*0325 |
| 32 | DRB1*0132 | HLA-DPA1*0103-DPB1*1901 | HLA-DQA1*0101-DQB1*0326 |
| 33 | DRB1*0301 | HLA-DPA1*0103-DPB1*2101 | HLA-DQA1*0101-DQB1*0327 |
| 34 | DRB1*0302 | HLA-DPA1*0103-DPB1*2201 | HLA-DQA1*0101-DQB1*0328 |
| 35 | DRB1*0303 | HLA-DPA1*0103-DPB1*2301 | HLA-DQA1*0101-DQB1*0329 |
| 36 | DRB1*0304 | HLA-DPA1*0103-DPB1*2401 | HLA-DQA1*0101-DQB1*0330 |
| 37 | DRB1*0305 | HLA-DPA1*0103-DPB1*2501 | HLA-DQA1*0101-DQB1*0331 |
| 38 | DRB1*0306 | HLA-DPA1*0103-DPB1*2601 | HLA-DQA1*0101-DQB1*0332 |
| 39 | DRB1*0307 | HLA-DPA1*0103-DPB1*2701 | HLA-DQA1*0101-DQB1*0333 |
| 40 | DRB1*0308 | HLA-DPA1*0103-DPB1*2801 | HLA-DQA1*0101-DQB1*0334 |
| 41 | DRB1*0310 | HLA-DPA1*0103-DPB1*2901 | HLA-DQA1*0101-DQB1*0335 |
| 42 | DRB1*0311 | HLA-DPA1*0103-DPB1*3001 | HLA-DQA1*0101-DQB1*0336 |
| 43 | DRB1*0313 | HLA-DPA1*0103-DPB1*1301 | HLA-DQA1*0101-DQB1*0337 |
| 44 | DRB1*0314 | HLA-DPA1*0103-DPB1*3101 | HLA-DQA1*0101-DQB1*0338 |
| 45 | DRB1*0315 | HLA-DPA1*0103-DPB1*3201 | HLA-DQA1*0101-DQB1*0401 |
| 46 | DRB1*0317 | HLA-DPA1*0103-DPB1*3301 | HLA-DQA1*0101-DQB1*0402 |
| 47 | DRB1*0318 | HLA-DPA1*0103-DPB1*3401 | HLA-DQA1*0101-DQB1*0403 |
| 48 | DRB1*0319 | HLA-DPA1*0103-DPB1*1401 | HLA-DQA1*0101-DQB1*0404 |
| 49 | DRB1*0320 | HLA-DPA1*0103-DPB1*1501 | HLA-DQA1*0101-DQB1*0405 |
| 50 | DRB1*0321 | HLA-DPA1*0103-DPB1*1601 | HLA-DQA1*0101-DQB1*0406 |
| 51 | DRB1*0322 | HLA-DPA1*0103-DPB1*1701 | HLA-DQA1*0101-DQB1*0407 |
| 52 | DRB1*0323 | HLA-DPA1*0103-DPB1*1801 | HLA-DQA1*0101-DQB1*0408 |
| 53 | DRB1*0324 | HLA-DPA1*0103-DPB1*1901 | HLA-DQA1*0101-DQB1*0501 |
| 54 | DRB1*0325 | HLA-DPA1*0103-DPB1*2001 | HLA-DQA1*0101-DQB1*0502 |
| 55 | DRB1*0326 | HLA-DPA1*0103-DPB1*2101 | HLA-DQA1*0101-DQB1*0503 |
| 56 | DRB1*0327 | HLA-DPA1*0103-DPB1*2201 | HLA-DQA1*0101-DQB1*0505 |
| 57 | DRB1*0328 | HLA-DPA1*0103-DPB1*2301 | HLA-DQA1*0101-DQB1*0506 |
| 58 | DRB1*0329 | HLA-DPA1*0103-DPB1*2401 | HLA-DQA1*0101-DQB1*0507 |
| 59 | DRB1*0330 | HLA-DPA1*0103-DPB1*2501 | HLA-DQA1*0101-DQB1*0508 |
| 60 | DRB1*0331 | HLA-DPA1*0103-DPB1*2601 | HLA-DQA1*0101-DQB1*0509 |
| 61 | DRB1*0332 | HLA-DPA1*0103-DPB1*2701 | HLA-DQA1*0101-DQB1*0510 |
| 62 | DRB1*0333 | HLA-DPA1*0103-DPB1*2801 | HLA-DQA1*0101-DQB1*0511 |
| 63 | DRB1*0334 | HLA-DPA1*0103-DPB1*2901 | HLA-DQA1*0101-DQB1*0512 |
| 64 | DRB1*0335 | HLA-DPA1*0103-DPB1*3001 | HLA-DQA1*0101-DQB1*0513 |
| 65 | DRB1*0336 | HLA-DPA1*0103-DPB1*3101 | HLA-DQA1*0101-DQB1*0514 |
| 66 | DRB1*0337 | HLA-DPA1*0103-DPB1*3201 | HLA-DQA1*0101-DQB1*0601 |
| 67 | DRB1*0338 | HLA-DPA1*0103-DPB1*3301 | HLA-DQA1*0101-DQB1*0602 |
| 68 | DRB1*0339 | HLA-DPA1*0103-DPB1*3401 | HLA-DQA1*0101-DQB1*0603 |
| 69 | DRB1*0340 | HLA-DPA1*0103-DPB1*3501 | HLA-DQA1*0101-DQB1*0604 |
| 70 | DRB1*0341 | HLA-DPA1*0103-DPB1*3601 | HLA-DQA1*0101-DQB1*0607 |
| 71 | DRB1*0342 | HLA-DPA1*0103-DPB1*3701 | HLA-DQA1*0101-DQB1*0608 |
| 72 | DRB1*0343 | HLA-DPA1*0103-DPB1*3801 | HLA-DQA1*0101-DQB1*0609 |
| 73 | DRB1*0344 | HLA-DPA1*0103-DPB1*3901 | HLA-DQA1*0101-DQB1*0610 |
| 74 | DRB1*0345 | HLA-DPA1*0103-DPB1*4001 | HLA-DQA1*0101-DQB1*0611 |
| 75 | DRB1*0346 | HLA-DPA1*0103-DPB1*4101 | HLA-DQA1*0101-DQB1*0612 |
| 76 | DRB1*0347 | HLA-DPA1*0103-DPB1*4401 | HLA-DQA1*0101-DQB1*0614 |
| 77 | DRB1*0348 | HLA-DPA1*0103-DPB1*4501 | HLA-DQA1*0101-DQB1*0615 |
| 78 | DRB1*0349 | HLA-DPA1*0103-DPB1*4601 | HLA-DQA1*0101-DQB1*0616 |
| 79 | DRB1*0350 | HLA-DPA1*0103-DPB1*4701 | HLA-DQA1*0101-DQB1*0617 |
| 80 | DRB1*0351 | HLA-DPA1*0103-DPB1*4801 | HLA-DQA1*0101-DQB1*0618 |
| 81 | DRB1*0352 | HLA-DPA1*0103-DPB1*4901 | HLA-DQA1*0101-DQB1*0619 |
| 82 | DRB1*0353 | HLA-DPA1*0103-DPB1*5001 | HLA-DQA1*0101-DQB1*0621 |
| 83 | DRB1*0354 | HLA-DPA1*0103-DPB1*5101 | HLA-DQA1*0101-DQB1*0622 |
| 84 | DRB1*0355 | HLA-DPA1*0103-DPB1*5201 | HLA-DQA1*0101-DQB1*0623 |
| 85 | DRB1*0401 | HLA-DPA1*0103-DPB1*5301 | HLA-DQA1*0101-DQB1*0624 |
| 86 | DRB1*0402 | HLA-DPA1*0103-DPB1*5401 | HLA-DQA1*0101-DQB1*0625 |
| 87 | DRB1*0403 | HLA-DPA1*0103-DPB1*5501 | HLA-DQA1*0101-DQB1*0627 |
| 88 | DRB1*0404 | HLA-DPA1*0103-DPB1*5601 | HLA-DQA1*0101-DQB1*0628 |
| 89 | DRB1*0405 | HLA-DPA1*0103-DPB1*5801 | HLA-DQA1*0101-DQB1*0629 |
| 90 | DRB1*0406 | HLA-DPA1*0103-DPB1*5901 | HLA-DQA1*0101-DQB1*0630 |
| 91 | DRB1*0407 | HLA-DPA1*0103-DPB1*6001 | HLA-DQA1*0101-DQB1*0631 |
| 92 | DRB1*0408 | HLA-DPA1*0103-DPB1*6201 | HLA-DQA1*0101-DQB1*0632 |
| 93 | DRB1*0409 | HLA-DPA1*0103-DPB1*6301 | HLA-DQA1*0101-DQB1*0633 |
| 94 | DRB1*0410 | HLA-DPA1*0103-DPB1*6501 | HLA-DQA1*0101-DQB1*0634 |
| 95 | DRB1*0411 | HLA-DPA1*0103-DPB1*6601 | HLA-DQA1*0101-DQB1*0635 |
| 96 | DRB1*0412 | HLA-DPA1*0103-DPB1*6701 | HLA-DQA1*0101-DQB1*0636 |
| 97 | DRB1*0413 | HLA-DPA1*0103-DPB1*6801 | HLA-DQA1*0101-DQB1*0637 |
| 98 | DRB1*0414 | HLA-DPA1*0103-DPB1*6901 | HLA-DQA1*0101-DQB1*0638 |
| 99 | DRB1*0415 | HLA-DPA1*0103-DPB1*7001 | HLA-DQA1*0101-DQB1*0639 |
| 100 | DRB1*0416 | HLA-DPA1*0103-DPB1*7101 | HLA-DQA1*0101-DQB1*0640 |
| 101 | DRB1*0417 | HLA-DPA1*0103-DPB1*7201 | HLA-DQA1*0101-DQB1*0641 |
| 102 | DRB1*0418 | HLA-DPA1*0103-DPB1*7301 | HLA-DQA1*0101-DQB1*0642 |
| 103 | DRB1*0419 | HLA-DPA1*0103-DPB1*7401 | HLA-DQA1*0101-DQB1*0643 |
| 104 | DRB1*0421 | HLA-DPA1*0103-DPB1*7501 | HLA-DQA1*0101-DQB1*0644 |
| 105 | DRB1*0422 | HLA-DPA1*0103-DPB1*7601 | HLA-DQA1*0102-DQB1*0201 |
| 106 | DRB1*0423 | HLA-DPA1*0103-DPB1*7701 | HLA-DQA1*0102-DQB1*0202 |
| 107 | DRB1*0424 | HLA-DPA1*0103-DPB1*7801 | HLA-DQA1*0102-DQB1*0203 |
| 108 | DRB1*0426 | HLA-DPA1*0103-DPB1*7901 | HLA-DQA1*0102-DQB1*0204 |
| 109 | DRB1*0427 | HLA-DPA1*0103-DPB1*8001 | HLA-DQA1*0102-DQB1*0205 |
| 110 | DRB1*0428 | HLA-DPA1*0103-DPB1*8101 | HLA-DQA1*0102-DQB1*0206 |
| 111 | DRB1*0429 | HLA-DPA1*0103-DPB1*8201 | HLA-DQA1*0102-DQB1*0301 |
| 112 | DRB1*0430 | HLA-DPA1*0103-DPB1*8301 | HLA-DQA1*0102-DQB1*0302 |
| 113 | DRB1*0431 | HLA-DPA1*0103-DPB1*8401 | HLA-DQA1*0102-DQB1*0303 |
| 114 | DRB1*0433 | HLA-DPA1*0103-DPB1*8501 | HLA-DQA1*0102-DQB1*0304 |
| 115 | DRB1*0434 | HLA-DPA1*0103-DPB1*8601 | HLA-DQA1*0102-DQB1*0305 |
| 116 | DRB1*0435 | HLA-DPA1*0103-DPB1*8701 | HLA-DQA1*0102-DQB1*0306 |
| 117 | DRB1*0436 | HLA-DPA1*0103-DPB1*8801 | HLA-DQA1*0102-DQB1*0307 |
| 118 | DRB1*0437 | HLA-DPA1*0103-DPB1*8901 | HLA-DQA1*0102-DQB1*0308 |
| 119 | DRB1*0438 | HLA-DPA1*0103-DPB1*9001 | HLA-DQA1*0102-DQB1*0309 |
| 120 | DRB1*0439 | HLA-DPA1*0103-DPB1*9101 | HLA-DQA1*0102-DQB1*0310 |
| 121 | DRB1*0440 | HLA-DPA1*0103-DPB1*9201 | HLA-DQA1*0102-DQB1*0311 |
| 122 | DRB1*0441 | HLA-DPA1*0103-DPB1*9301 | HLA-DQA1*0102-DQB1*0312 |
| 123 | DRB1*0442 | HLA-DPA1*0103-DPB1*9401 | HLA-DQA1*0102-DQB1*0313 |
| 124 | DRB1*0443 | HLA-DPA1*0103-DPB1*9501 | HLA-DQA1*0102-DQB1*0314 |
| 125 | DRB1*0444 | HLA-DPA1*0103-DPB1*9601 | HLA-DQA1*0102-DQB1*0315 |
| 126 | DRB1*0445 | HLA-DPA1*0103-DPB1*9701 | HLA-DQA1*0102-DQB1*0316 |
| 127 | DRB1*0446 | HLA-DPA1*0103-DPB1*9801 | HLA-DQA1*0102-DQB1*0317 |
| 128 | DRB1*0447 | HLA-DPA1*0103-DPB1*9901 | HLA-DQA1*0102-DQB1*0318 |
| 129 | DRB1*0448 | HLA-DPA1*0104-DPB1*0101 | HLA-DQA1*0102-DQB1*0319 |
| 130 | DRB1*0449 | HLA-DPA1*0104-DPB1*0201 | HLA-DQA1*0102-DQB1*0320 |
| 131 | DRB1*0450 | HLA-DPA1*0104-DPB1*0202 | HLA-DQA1*0102-DQB1*0321 |
| 132 | DRB1*0451 | HLA-DPA1*0104-DPB1*0301 | HLA-DQA1*0102-DQB1*0322 |
| 133 | DRB1*0452 | HLA-DPA1*0104-DPB1*0401 | HLA-DQA1*0102-DQB1*0323 |
| 134 | DRB1*0453 | HLA-DPA1*0104-DPB1*0402 | HLA-DQA1*0102-DQB1*0324 |
| 135 | DRB1*0454 | HLA-DPA1*0104-DPB1*0501 | HLA-DQA1*0102-DQB1*0325 |
| 136 | DRB1*0455 | HLA-DPA1*0104-DPB1*0601 | HLA-DQA1*0102-DQB1*0326 |
| 137 | DRB1*0456 | HLA-DPA1*0104-DPB1*0801 | HLA-DQA1*0102-DQB1*0327 |
| 138 | DRB1*0457 | HLA-DPA1*0104-DPB1*0901 | HLA-DQA1*0102-DQB1*0328 |
| 139 | DRB1*0458 | HLA-DPA1*0104-DPB1*0001 | HLA-DQA1*0102-DQB1*0329 |
| 140 | DRB1*0459 | HLA-DPA1*0104-DPB1*1001 | HLA-DQA1*0102-DQB1*0330 |
| 141 | DRB1*0460 | HLA-DPA1*0104-DPB1*0101 | HLA-DQA1*0102-DQB1*0331 |
| 142 | DRB1*0461 | HLA-DPA1*0104-DPB1*0201 | HLA-DQA1*0102-DQB1*0332 |
| 143 | DRB1*0462 | HLA-DPA1*0104-DPB1*0301 | HLA-DQA1*0102-DQB1*0333 |
| 144 | DRB1*0463 | HLA-DPA1*0104-DPB1*0401 | HLA-DQA1*0102-DQB1*0334 |
| 145 | DRB1*0464 | HLA-DPA1*0104-DPB1*0501 | HLA-DQA1*0102-DQB1*0335 |
| 146 | DRB1*0465 | HLA-DPA1*0104-DPB1*0601 | HLA-DQA1*0102-DQB1*0336 |
| 147 | DRB1*0466 | HLA-DPA1*0104-DPB1*0701 | HLA-DQA1*0102-DQB1*0337 |
| 148 | DRB1*0467 | HLA-DPA1*0104-DPB1*0801 | HLA-DQA1*0102-DQB1*0338 |
| 149 | DRB1*0468 | HLA-DPA1*0104-DPB1*0901 | HLA-DQA1*0102-DQB1*0401 |
| 150 | DRB1*0469 | HLA-DPA1*0104-DPB1*1001 | HLA-DQA1*0102-DQB1*0402 |
| 151 | DRB1*0470 | HLA-DPA1*0104-DPB1*1101 | HLA-DQA1*0102-DQB1*0403 |
| 152 | DRB1*0471 | HLA-DPA1*0104-DPB1*1101 | HLA-DQA1*0102-DQB1*0404 |
| 153 | DRB1*0472 | HLA-DPA1*0104-DPB1*1201 | HLA-DQA1*0102-DQB1*0405 |
| 154 | DRB1*0473 | HLA-DPA1*0104-DPB1*1301 | HLA-DQA1*0102-DQB1*0406 |
| 155 | DRB1*0474 | HLA-DPA1*0104-DPB1*1401 | HLA-DQA1*0102-DQB1*0407 |
| 156 | DRB1*0475 | HLA-DPA1*0104-DPB1*1501 | HLA-DQA1*0102-DQB1*0408 |
| 157 | DRB1*0476 | HLA-DPA1*0104-DPB1*1601 | HLA-DQA1*0102-DQB1*0501 |
| 158 | DRB1*0477 | HLA-DPA1*0104-DPB1*1701 | HLA-DQA1*0102-DQB1*0502 |
| 159 | DRB1*0478 | HLA-DPA1*0104-DPB1*1801 | HLA-DQA1*0102-DQB1*0503 |
| 160 | DRB1*0479 | HLA-DPA1*0104-DPB1*1901 | HLA-DQA1*0102-DQB1*0505 |
| 161 | DRB1*0480 | HLA-DPA1*0104-DPB1*2101 | HLA-DQA1*0102-DQB1*0506 |
| 162 | DRB1*0482 | HLA-DPA1*0104-DPB1*2201 | HLA-DQA1*0102-DQB1*0507 |
| 163 | DRB1*0483 | HLA-DPA1*0104-DPB1*2301 | HLA-DQA1*0102-DQB1*0508 |
| 164 | DRB1*0484 | HLA-DPA1*0104-DPB1*2401 | HLA-DQA1*0102-DQB1*0509 |
| 165 | DRB1*0485 | HLA-DPA1*0104-DPB1*2501 | HLA-DQA1*0102-DQB1*0510 |
| 166 | DRB1*0486 | HLA-DPA1*0104-DPB1*2601 | HLA-DQA1*0102-DQB1*0511 |
| 167 | DRB1*0487 | HLA-DPA1*0104-DPB1*2701 | HLA-DQA1*0102-DQB1*0512 |
| 168 | DRB1*0488 | HLA-DPA1*0104-DPB1*2801 | HLA-DQA1*0102-DQB1*0513 |
| 169 | DRB1*0489 | HLA-DPA1*0104-DPB1*2901 | HLA-DQA1*0102-DQB1*0514 |
| 170 | DRB1*0491 | HLA-DPA1*0104-DPB1*3001 | HLA-DQA1*0102-DQB1*0601 |
| 171 | DRB1*0701 | HLA-DPA1*0104-DPB1*1301 | HLA-DQA1*0102-DQB1*0602 |
| 172 | DRB1*0703 | HLA-DPA1*0104-DPB1*3101 | HLA-DQA1*0102-DQB1*0603 |
| 173 | DRB1*0704 | HLA-DPA1*0104-DPB1*3201 | HLA-DQA1*0102-DQB1*0604 |
| 174 | DRB1*0705 | HLA-DPA1*0104-DPB1*3301 | HLA-DQA1*0102-DQB1*0607 |
| 175 | DRB1*0706 | HLA-DPA1*0104-DPB1*3401 | HLA-DQA1*0102-DQB1*0608 |
| 176 | DRB1*0707 | HLA-DPA1*0104-DPB1*1401 | HLA-DQA1*0102-DQB1*0609 |
| 177 | DRB1*0708 | HLA-DPA1*0104-DPB1*1501 | HLA-DQA1*0102-DQB1*0610 |
| 178 | DRB1*0709 | HLA-DPA1*0104-DPB1*1601 | HLA-DQA1*0102-DQB1*0611 |
| 179 | DRB1*0711 | HLA-DPA1*0104-DPB1*1701 | HLA-DQA1*0102-DQB1*0612 |
| 180 | DRB1*0712 | HLA-DPA1*0104-DPB1*1801 | HLA-DQA1*0102-DQB1*0614 |
| 181 | DRB1*0713 | HLA-DPA1*0104-DPB1*1901 | HLA-DQA1*0102-DQB1*0615 |
| 182 | DRB1*0714 | HLA-DPA1*0104-DPB1*2001 | HLA-DQA1*0102-DQB1*0616 |
| 183 | DRB1*0715 | HLA-DPA1*0104-DPB1*2101 | HLA-DQA1*0102-DQB1*0617 |
| 184 | DRB1*0716 | HLA-DPA1*0104-DPB1*2201 | HLA-DQA1*0102-DQB1*0618 |
| 185 | DRB1*0717 | HLA-DPA1*0104-DPB1*2301 | HLA-DQA1*0102-DQB1*0619 |
| 186 | DRB1*0719 | HLA-DPA1*0104-DPB1*2401 | HLA-DQA1*0102-DQB1*0621 |
| 187 | DRB1*0801 | HLA-DPA1*0104-DPB1*2501 | HLA-DQA1*0102-DQB1*0622 |
| 188 | DRB1*0802 | HLA-DPA1*0104-DPB1*2601 | HLA-DQA1*0102-DQB1*0623 |
| 189 | DRB1*0803 | HLA-DPA1*0104-DPB1*2701 | HLA-DQA1*0102-DQB1*0624 |
| 190 | DRB1*0804 | HLA-DPA1*0104-DPB1*2801 | HLA-DQA1*0102-DQB1*0625 |
| 191 | DRB1*0805 | HLA-DPA1*0104-DPB1*2901 | HLA-DQA1*0102-DQB1*0627 |
| 192 | DRB1*0806 | HLA-DPA1*0104-DPB1*3001 | HLA-DQA1*0102-DQB1*0628 |
| 193 | DRB1*0807 | HLA-DPA1*0104-DPB1*3101 | HLA-DQA1*0102-DQB1*0629 |
| 194 | DRB1*0808 | HLA-DPA1*0104-DPB1*3201 | HLA-DQA1*0102-DQB1*0630 |
| 195 | DRB1*0809 | HLA-DPA1*0104-DPB1*3301 | HLA-DQA1*0102-DQB1*0631 |
| 196 | DRB1*0810 | HLA-DPA1*0104-DPB1*3401 | HLA-DQA1*0102-DQB1*0632 |
| 197 | DRB1*0811 | HLA-DPA1*0104-DPB1*3501 | HLA-DQA1*0102-DQB1*0633 |
| 198 | DRB1*0812 | HLA-DPA1*0104-DPB1*3601 | HLA-DQA1*0102-DQB1*0634 |
| 199 | DRB1*0813 | HLA-DPA1*0104-DPB1*3701 | HLA-DQA1*0102-DQB1*0635 |
| 200 | DRB1*0814 | HLA-DPA1*0104-DPB1*3801 | HLA-DQA1*0102-DQB1*0636 |
| 201 | DRB1*0815 | HLA-DPA1*0104-DPB1*3901 | HLA-DQA1*0102-DQB1*0637 |
| 202 | DRB1*0816 | HLA-DPA1*0104-DPB1*4001 | HLA-DQA1*0102-DQB1*0638 |
| 203 | DRB1*0818 | HLA-DPA1*0104-DPB1*4101 | HLA-DQA1*0102-DQB1*0639 |
| 204 | DRB1*0819 | HLA-DPA1*0104-DPB1*4401 | HLA-DQA1*0102-DQB1*0640 |
| 205 | DRB1*0820 | HLA-DPA1*0104-DPB1*4501 | HLA-DQA1*0102-DQB1*0641 |
| 206 | DRB1*0821 | HLA-DPA1*0104-DPB1*4601 | HLA-DQA1*0102-DQB1*0642 |
| 207 | DRB1*0822 | HLA-DPA1*0104-DPB1*4701 | HLA-DQA1*0102-DQB1*0643 |
| 208 | DRB1*0823 | HLA-DPA1*0104-DPB1*4801 | HLA-DQA1*0102-DQB1*0644 |
| 209 | DRB1*0824 | HLA-DPA1*0104-DPB1*4901 | HLA-DQA1*0103-DQB1*0201 |
| 210 | DRB1*0825 | HLA-DPA1*0104-DPB1*5001 | HLA-DQA1*0103-DQB1*0202 |
| 211 | DRB1*0826 | HLA-DPA1*0104-DPB1*5101 | HLA-DQA1*0103-DQB1*0203 |
| 212 | DRB1*0827 | HLA-DPA1*0104-DPB1*5201 | HLA-DQA1*0103-DQB1*0204 |
| 213 | DRB1*0828 | HLA-DPA1*0104-DPB1*5301 | HLA-DQA1*0103-DQB1*0205 |
| 214 | DRB1*0829 | HLA-DPA1*0104-DPB1*5401 | HLA-DQA1*0103-DQB1*0206 |
| 215 | DRB1*0830 | HLA-DPA1*0104-DPB1*5501 | HLA-DQA1*0103-DQB1*0301 |
| 216 | DRB1*0831 | HLA-DPA1*0104-DPB1*5601 | HLA-DQA1*0103-DQB1*0302 |
| 217 | DRB1*0832 | HLA-DPA1*0104-DPB1*5801 | HLA-DQA1*0103-DQB1*0303 |
| 218 | DRB1*0833 | HLA-DPA1*0104-DPB1*5901 | HLA-DQA1*0103-DQB1*0304 |
| 219 | DRB1*0834 | HLA-DPA1*0104-DPB1*6001 | HLA-DQA1*0103-DQB1*0305 |
| 220 | DRB1*0835 | HLA-DPA1*0104-DPB1*6201 | HLA-DQA1*0103-DQB1*0306 |
| 221 | DRB1*0836 | HLA-DPA1*0104-DPB1*6301 | HLA-DQA1*0103-DQB1*0307 |
| 222 | DRB1*0837 | HLA-DPA1*0104-DPB1*6501 | HLA-DQA1*0103-DQB1*0308 |
| 223 | DRB1*0838 | HLA-DPA1*0104-DPB1*6601 | HLA-DQA1*0103-DQB1*0309 |
| 224 | DRB1*0839 | HLA-DPA1*0104-DPB1*6701 | HLA-DQA1*0103-DQB1*0310 |
| 225 | DRB1*0840 | HLA-DPA1*0104-DPB1*6801 | HLA-DQA1*0103-DQB1*0311 |
| 226 | DRB1*0901 | HLA-DPA1*0104-DPB1*6901 | HLA-DQA1*0103-DQB1*0312 |
| 227 | DRB1*0902 | HLA-DPA1*0104-DPB1*7001 | HLA-DQA1*0103-DQB1*0313 |
| 228 | DRB1*0903 | HLA-DPA1*0104-DPB1*7101 | HLA-DQA1*0103-DQB1*0314 |
| 229 | DRB1*0904 | HLA-DPA1*0104-DPB1*7201 | HLA-DQA1*0103-DQB1*0315 |
| 230 | DRB1*0905 | HLA-DPA1*0104-DPB1*7301 | HLA-DQA1*0103-DQB1*0316 |
| 231 | DRB1*0906 | HLA-DPA1*0104-DPB1*7401 | HLA-DQA1*0103-DQB1*0317 |
| 232 | DRB1*0907 | HLA-DPA1*0104-DPB1*7501 | HLA-DQA1*0103-DQB1*0318 |
| 233 | DRB1*0908 | HLA-DPA1*0104-DPB1*7601 | HLA-DQA1*0103-DQB1*0319 |
| 234 | DRB1*0909 | HLA-DPA1*0104-DPB1*7701 | HLA-DQA1*0103-DQB1*0320 |
| 235 | DRB1*1001 | HLA-DPA1*0104-DPB1*7801 | HLA-DQA1*0103-DQB1*0321 |
| 236 | DRB1*1002 | HLA-DPA1*0104-DPB1*7901 | HLA-DQA1*0103-DQB1*0322 |
| 237 | DRB1*1003 | HLA-DPA1*0104-DPB1*8001 | HLA-DQA1*0103-DQB1*0323 |
| 238 | DRB1*1101 | HLA-DPA1*0104-DPB1*8101 | HLA-DQA1*0103-DQB1*0324 |
| 239 | DRB1*1102 | HLA-DPA1*0104-DPB1*8201 | HLA-DQA1*0103-DQB1*0325 |
| 240 | DRB1*1103 | HLA-DPA1*0104-DPB1*8301 | HLA-DQA1*0103-DQB1*0326 |
| 241 | DRB1*1104 | HLA-DPA1*0104-DPB1*8401 | HLA-DQA1*0103-DQB1*0327 |
| 242 | DRB1*1105 | HLA-DPA1*0104-DPB1*8501 | HLA-DQA1*0103-DQB1*0328 |
| 243 | DRB1*1106 | HLA-DPA1*0104-DPB1*8601 | HLA-DQA1*0103-DQB1*0329 |
| 244 | DRB1*1107 | HLA-DPA1*0104-DPB1*8701 | HLA-DQA1*0103-DQB1*0330 |
| 245 | DRB1*1108 | HLA-DPA1*0104-DPB1*8801 | HLA-DQA1*0103-DQB1*0331 |
| 246 | DRB1*1109 | HLA-DPA1*0104-DPB1*8901 | HLA-DQA1*0103-DQB1*0332 |
| 247 | DRB1*1110 | HLA-DPA1*0104-DPB1*9001 | HLA-DQA1*0103-DQB1*0333 |
| 248 | DRB1*1111 | HLA-DPA1*0104-DPB1*9101 | HLA-DQA1*0103-DQB1*0334 |
| 249 | DRB1*1112 | HLA-DPA1*0104-DPB1*9201 | HLA-DQA1*0103-DQB1*0335 |
| 250 | DRB1*1113 | HLA-DPA1*0104-DPB1*9301 | HLA-DQA1*0103-DQB1*0336 |
| 251 | DRB1*1114 | HLA-DPA1*0104-DPB1*9401 | HLA-DQA1*0103-DQB1*0337 |
| 252 | DRB1*1115 | HLA-DPA1*0104-DPB1*9501 | HLA-DQA1*0103-DQB1*0338 |
| 253 | DRB1*1116 | HLA-DPA1*0104-DPB1*9601 | HLA-DQA1*0103-DQB1*0401 |
| 254 | DRB1*1117 | HLA-DPA1*0104-DPB1*9701 | HLA-DQA1*0103-DQB1*0402 |
| 255 | DRB1*1118 | HLA-DPA1*0104-DPB1*9801 | HLA-DQA1*0103-DQB1*0403 |
| 256 | DRB1*1119 | HLA-DPA1*0104-DPB1*9901 | HLA-DQA1*0103-DQB1*0404 |
| 257 | DRB1*1120 | HLA-DPA1*0105-DPB1*0101 | HLA-DQA1*0103-DQB1*0405 |
| 258 | DRB1*1121 | HLA-DPA1*0105-DPB1*0201 | HLA-DQA1*0103-DQB1*0406 |
| 259 | DRB1*1124 | HLA-DPA1*0105-DPB1*0202 | HLA-DQA1*0103-DQB1*0407 |
| 260 | DRB1*1125 | HLA-DPA1*0105-DPB1*0301 | HLA-DQA1*0103-DQB1*0408 |
| 261 | DRB1*1127 | HLA-DPA1*0105-DPB1*0401 | HLA-DQA1*0103-DQB1*0501 |
| 262 | DRB1*1128 | HLA-DPA1*0105-DPB1*0402 | HLA-DQA1*0103-DQB1*0502 |
| 263 | DRB1*1129 | HLA-DPA1*0105-DPB1*0501 | HLA-DQA1*0103-DQB1*0503 |
| 264 | DRB1*1130 | HLA-DPA1*0105-DPB1*0601 | HLA-DQA1*0103-DQB1*0505 |
| 265 | DRB1*1131 | HLA-DPA1*0105-DPB1*0801 | HLA-DQA1*0103-DQB1*0506 |
| 266 | DRB1*1132 | HLA-DPA1*0105-DPB1*0901 | HLA-DQA1*0103-DQB1*0507 |
| 267 | DRB1*1133 | HLA-DPA1*0105-DPB1*0001 | HLA-DQA1*0103-DQB1*0508 |
| 268 | DRB1*1134 | HLA-DPA1*0105-DPB1*1001 | HLA-DQA1*0103-DQB1*0509 |
| 269 | DRB1*1135 | HLA-DPA1*0105-DPB1*0101 | HLA-DQA1*0103-DQB1*0510 |
| 270 | DRB1*1136 | HLA-DPA1*0105-DPB1*0201 | HLA-DQA1*0103-DQB1*0511 |
| 271 | DRB1*1137 | HLA-DPA1*0105-DPB1*0301 | HLA-DQA1*0103-DQB1*0512 |
| 272 | DRB1*1138 | HLA-DPA1*0105-DPB1*0401 | HLA-DQA1*0103-DQB1*0513 |
| 273 | DRB1*1139 | HLA-DPA1*0105-DPB1*0501 | HLA-DQA1*0103-DQB1*0514 |
| 274 | DRB1*1141 | HLA-DPA1*0105-DPB1*0601 | HLA-DQA1*0103-DQB1*0601 |
| 275 | DRB1*1142 | HLA-DPA1*0105-DPB1*0701 | HLA-DQA1*0103-DQB1*0602 |
| 276 | DRB1*1143 | HLA-DPA1*0105-DPB1*0801 | HLA-DQA1*0103-DQB1*0603 |
| 277 | DRB1*1144 | HLA-DPA1*0105-DPB1*0901 | HLA-DQA1*0103-DQB1*0604 |
| 278 | DRB1*1145 | HLA-DPA1*0105-DPB1*1001 | HLA-DQA1*0103-DQB1*0607 |
| 279 | DRB1*1146 | HLA-DPA1*0105-DPB1*1101 | HLA-DQA1*0103-DQB1*0608 |
| 280 | DRB1*1147 | HLA-DPA1*0105-DPB1*1101 | HLA-DQA1*0103-DQB1*0609 |
| 281 | DRB1*1148 | HLA-DPA1*0105-DPB1*1201 | HLA-DQA1*0103-DQB1*0610 |
| 282 | DRB1*1149 | HLA-DPA1*0105-DPB1*1301 | HLA-DQA1*0103-DQB1*0611 |
| 283 | DRB1*1150 | HLA-DPA1*0105-DPB1*1401 | HLA-DQA1*0103-DQB1*0612 |
| 284 | DRB1*1151 | HLA-DPA1*0105-DPB1*1501 | HLA-DQA1*0103-DQB1*0614 |
| 285 | DRB1*1152 | HLA-DPA1*0105-DPB1*1601 | HLA-DQA1*0103-DQB1*0615 |
| 286 | DRB1*1153 | HLA-DPA1*0105-DPB1*1701 | HLA-DQA1*0103-DQB1*0616 |
| 287 | DRB1*1154 | HLA-DPA1*0105-DPB1*1801 | HLA-DQA1*0103-DQB1*0617 |
| 288 | DRB1*1155 | HLA-DPA1*0105-DPB1*1901 | HLA-DQA1*0103-DQB1*0618 |
| 289 | DRB1*1156 | HLA-DPA1*0105-DPB1*2101 | HLA-DQA1*0103-DQB1*0619 |
| 290 | DRB1*1157 | HLA-DPA1*0105-DPB1*2201 | HLA-DQA1*0103-DQB1*0621 |
| 291 | DRB1*1158 | HLA-DPA1*0105-DPB1*2301 | HLA-DQA1*0103-DQB1*0622 |
| 292 | DRB1*1159 | HLA-DPA1*0105-DPB1*2401 | HLA-DQA1*0103-DQB1*0623 |
| 293 | DRB1*1160 | HLA-DPA1*0105-DPB1*2501 | HLA-DQA1*0103-DQB1*0624 |
| 294 | DRB1*1161 | HLA-DPA1*0105-DPB1*2601 | HLA-DQA1*0103-DQB1*0625 |
| 295 | DRB1*1162 | HLA-DPA1*0105-DPB1*2701 | HLA-DQA1*0103-DQB1*0627 |
| 296 | DRB1*1163 | HLA-DPA1*0105-DPB1*2801 | HLA-DQA1*0103-DQB1*0628 |
| 297 | DRB1*1164 | HLA-DPA1*0105-DPB1*2901 | HLA-DQA1*0103-DQB1*0629 |
| 298 | DRB1*1165 | HLA-DPA1*0105-DPB1*3001 | HLA-DQA1*0103-DQB1*0630 |
| 299 | DRB1*1166 | HLA-DPA1*0105-DPB1*1301 | HLA-DQA1*0103-DQB1*0631 |
| 300 | DRB1*1167 | HLA-DPA1*0105-DPB1*3101 | HLA-DQA1*0103-DQB1*0632 |
| 301 | DRB1*1168 | HLA-DPA1*0105-DPB1*3201 | HLA-DQA1*0103-DQB1*0633 |
| 302 | DRB1*1169 | HLA-DPA1*0105-DPB1*3301 | HLA-DQA1*0103-DQB1*0634 |
| 303 | DRB1*1170 | HLA-DPA1*0105-DPB1*3401 | HLA-DQA1*0103-DQB1*0635 |
| 304 | DRB1*1172 | HLA-DPA1*0105-DPB1*1401 | HLA-DQA1*0103-DQB1*0636 |
| 305 | DRB1*1173 | HLA-DPA1*0105-DPB1*1501 | HLA-DQA1*0103-DQB1*0637 |
| 306 | DRB1*1174 | HLA-DPA1*0105-DPB1*1601 | HLA-DQA1*0103-DQB1*0638 |
| 307 | DRB1*1175 | HLA-DPA1*0105-DPB1*1701 | HLA-DQA1*0103-DQB1*0639 |
| 308 | DRB1*1176 | HLA-DPA1*0105-DPB1*1801 | HLA-DQA1*0103-DQB1*0640 |
| 309 | DRB1*1177 | HLA-DPA1*0105-DPB1*1901 | HLA-DQA1*0103-DQB1*0641 |
| 310 | DRB1*1178 | HLA-DPA1*0105-DPB1*2001 | HLA-DQA1*0103-DQB1*0642 |
| 311 | DRB1*1179 | HLA-DPA1*0105-DPB1*2101 | HLA-DQA1*0103-DQB1*0643 |
| 312 | DRB1*1180 | HLA-DPA1*0105-DPB1*2201 | HLA-DQA1*0103-DQB1*0644 |
| 313 | DRB1*1181 | HLA-DPA1*0105-DPB1*2301 | HLA-DQA1*0104-DQB1*0201 |
| 314 | DRB1*1182 | HLA-DPA1*0105-DPB1*2401 | HLA-DQA1*0104-DQB1*0202 |
| 315 | DRB1*1183 | HLA-DPA1*0105-DPB1*2501 | HLA-DQA1*0104-DQB1*0203 |
| 316 | DRB1*1184 | HLA-DPA1*0105-DPB1*2601 | HLA-DQA1*0104-DQB1*0204 |
| 317 | DRB1*1185 | HLA-DPA1*0105-DPB1*2701 | HLA-DQA1*0104-DQB1*0205 |
| 318 | DRB1*1186 | HLA-DPA1*0105-DPB1*2801 | HLA-DQA1*0104-DQB1*0206 |
| 319 | DRB1*1187 | HLA-DPA1*0105-DPB1*2901 | HLA-DQA1*0104-DQB1*0301 |
| 320 | DRB1*1188 | HLA-DPA1*0105-DPB1*3001 | HLA-DQA1*0104-DQB1*0302 |
| 321 | DRB1*1189 | HLA-DPA1*0105-DPB1*3101 | HLA-DQA1*0104-DQB1*0303 |
| 322 | DRB1*1190 | HLA-DPA1*0105-DPB1*3201 | HLA-DQA1*0104-DQB1*0304 |
| 323 | DRB1*1191 | HLA-DPA1*0105-DPB1*3301 | HLA-DQA1*0104-DQB1*0305 |
| 324 | DRB1*1192 | HLA-DPA1*0105-DPB1*3401 | HLA-DQA1*0104-DQB1*0306 |
| 325 | DRB1*1193 | HLA-DPA1*0105-DPB1*3501 | HLA-DQA1*0104-DQB1*0307 |
| 326 | DRB1*1194 | HLA-DPA1*0105-DPB1*3601 | HLA-DQA1*0104-DQB1*0308 |
| 327 | DRB1*1195 | HLA-DPA1*0105-DPB1*3701 | HLA-DQA1*0104-DQB1*0309 |
| 328 | DRB1*1196 | HLA-DPA1*0105-DPB1*3801 | HLA-DQA1*0104-DQB1*0310 |
| 329 | DRB1*1201 | HLA-DPA1*0105-DPB1*3901 | HLA-DQA1*0104-DQB1*0311 |
| 330 | DRB1*1202 | HLA-DPA1*0105-DPB1*4001 | HLA-DQA1*0104-DQB1*0312 |
| 331 | DRB1*1203 | HLA-DPA1*0105-DPB1*4101 | HLA-DQA1*0104-DQB1*0313 |
| 332 | DRB1*1204 | HLA-DPA1*0105-DPB1*4401 | HLA-DQA1*0104-DQB1*0314 |
| 333 | DRB1*1205 | HLA-DPA1*0105-DPB1*4501 | HLA-DQA1*0104-DQB1*0315 |
| 334 | DRB1*1206 | HLA-DPA1*0105-DPB1*4601 | HLA-DQA1*0104-DQB1*0316 |
| 335 | DRB1*1207 | HLA-DPA1*0105-DPB1*4701 | HLA-DQA1*0104-DQB1*0317 |
| 336 | DRB1*1208 | HLA-DPA1*0105-DPB1*4801 | HLA-DQA1*0104-DQB1*0318 |
| 337 | DRB1*1209 | HLA-DPA1*0105-DPB1*4901 | HLA-DQA1*0104-DQB1*0319 |
| 338 | DRB1*1210 | HLA-DPA1*0105-DPB1*5001 | HLA-DQA1*0104-DQB1*0320 |
| 339 | DRB1*1211 | HLA-DPA1*0105-DPB1*5101 | HLA-DQA1*0104-DQB1*0321 |
| 340 | DRB1*1212 | HLA-DPA1*0105-DPB1*5201 | HLA-DQA1*0104-DQB1*0322 |
| 341 | DRB1*1213 | HLA-DPA1*0105-DPB1*5301 | HLA-DQA1*0104-DQB1*0323 |
| 342 | DRB1*1214 | HLA-DPA1*0105-DPB1*5401 | HLA-DQA1*0104-DQB1*0324 |
| 343 | DRB1*1215 | HLA-DPA1*0105-DPB1*5501 | HLA-DQA1*0104-DQB1*0325 |
| 344 | DRB1*1216 | HLA-DPA1*0105-DPB1*5601 | HLA-DQA1*0104-DQB1*0326 |
| 345 | DRB1*1217 | HLA-DPA1*0105-DPB1*5801 | HLA-DQA1*0104-DQB1*0327 |
| 346 | DRB1*1218 | HLA-DPA1*0105-DPB1*5901 | HLA-DQA1*0104-DQB1*0328 |
| 347 | DRB1*1219 | HLA-DPA1*0105-DPB1*6001 | HLA-DQA1*0104-DQB1*0329 |
| 348 | DRB1*1220 | HLA-DPA1*0105-DPB1*6201 | HLA-DQA1*0104-DQB1*0330 |
| 349 | DRB1*1221 | HLA-DPA1*0105-DPB1*6301 | HLA-DQA1*0104-DQB1*0331 |
| 350 | DRB1*1222 | HLA-DPA1*0105-DPB1*6501 | HLA-DQA1*0104-DQB1*0332 |
| 351 | DRB1*1223 | HLA-DPA1*0105-DPB1*6601 | HLA-DQA1*0104-DQB1*0333 |
| 352 | DRB1*1301 | HLA-DPA1*0105-DPB1*6701 | HLA-DQA1*0104-DQB1*0334 |
| 353 | DRB1*1302 | HLA-DPA1*0105-DPB1*6801 | HLA-DQA1*0104-DQB1*0335 |
| 354 | DRB1*1303 | HLA-DPA1*0105-DPB1*6901 | HLA-DQA1*0104-DQB1*0336 |
| 355 | DRB1*1304 | HLA-DPA1*0105-DPB1*7001 | HLA-DQA1*0104-DQB1*0337 |
| 356 | DRB1*1305 | HLA-DPA1*0105-DPB1*7101 | HLA-DQA1*0104-DQB1*0338 |
| 357 | DRB1*1306 | HLA-DPA1*0105-DPB1*7201 | HLA-DQA1*0104-DQB1*0401 |
| 358 | DRB1*1307 | HLA-DPA1*0105-DPB1*7301 | HLA-DQA1*0104-DQB1*0402 |
| 359 | DRB1*1308 | HLA-DPA1*0105-DPB1*7401 | HLA-DQA1*0104-DQB1*0403 |
| 360 | DRB1*1309 | HLA-DPA1*0105-DPB1*7501 | HLA-DQA1*0104-DQB1*0404 |
| 361 | DRB1*1310 | HLA-DPA1*0105-DPB1*7601 | HLA-DQA1*0104-DQB1*0405 |
| 362 | DRB1*13100 | HLA-DPA1*0105-DPB1*7701 | HLA-DQA1*0104-DQB1*0406 |
| 363 | DRB1*13101 | HLA-DPA1*0105-DPB1*7801 | HLA-DQA1*0104-DQB1*0407 |
| 364 | DRB1*1311 | HLA-DPA1*0105-DPB1*7901 | HLA-DQA1*0104-DQB1*0408 |
| 365 | DRB1*1312 | HLA-DPA1*0105-DPB1*8001 | HLA-DQA1*0104-DQB1*0501 |
| 366 | DRB1*1313 | HLA-DPA1*0105-DPB1*8101 | HLA-DQA1*0104-DQB1*0502 |
| 367 | DRB1*1314 | HLA-DPA1*0105-DPB1*8201 | HLA-DQA1*0104-DQB1*0503 |
| 368 | DRB1*1315 | HLA-DPA1*0105-DPB1*8301 | HLA-DQA1*0104-DQB1*0505 |
| 369 | DRB1*1316 | HLA-DPA1*0105-DPB1*8401 | HLA-DQA1*0104-DQB1*0506 |
| 370 | DRB1*1317 | HLA-DPA1*0105-DPB1*8501 | HLA-DQA1*0104-DQB1*0507 |
| 371 | DRB1*1318 | HLA-DPA1*0105-DPB1*8601 | HLA-DQA1*0104-DQB1*0508 |
| 372 | DRB1*1319 | HLA-DPA1*0105-DPB1*8701 | HLA-DQA1*0104-DQB1*0509 |
| 373 | DRB1*1320 | HLA-DPA1*0105-DPB1*8801 | HLA-DQA1*0104-DQB1*0510 |
| 374 | DRB1*1321 | HLA-DPA1*0105-DPB1*8901 | HLA-DQA1*0104-DQB1*0511 |
| 375 | DRB1*1322 | HLA-DPA1*0105-DPB1*9001 | HLA-DQA1*0104-DQB1*0512 |
| 376 | DRB1*1323 | HLA-DPA1*0105-DPB1*9101 | HLA-DQA1*0104-DQB1*0513 |
| 377 | DRB1*1324 | HLA-DPA1*0105-DPB1*9201 | HLA-DQA1*0104-DQB1*0514 |
| 378 | DRB1*1326 | HLA-DPA1*0105-DPB1*9301 | HLA-DQA1*0104-DQB1*0601 |
| 379 | DRB1*1327 | HLA-DPA1*0105-DPB1*9401 | HLA-DQA1*0104-DQB1*0602 |
| 380 | DRB1*1329 | HLA-DPA1*0105-DPB1*9501 | HLA-DQA1*0104-DQB1*0603 |
| 381 | DRB1*1330 | HLA-DPA1*0105-DPB1*9601 | HLA-DQA1*0104-DQB1*0604 |
| 382 | DRB1*1331 | HLA-DPA1*0105-DPB1*9701 | HLA-DQA1*0104-DQB1*0607 |
| 383 | DRB1*1332 | HLA-DPA1*0105-DPB1*9801 | HLA-DQA1*0104-DQB1*0608 |
| 384 | DRB1*1333 | HLA-DPA1*0105-DPB1*9901 | HLA-DQA1*0104-DQB1*0609 |
| 385 | DRB1*1334 | HLA-DPA1*0106-DPB1*0101 | HLA-DQA1*0104-DQB1*0610 |
| 386 | DRB1*1335 | HLA-DPA1*0106-DPB1*0201 | HLA-DQA1*0104-DQB1*0611 |
| 387 | DRB1*1336 | HLA-DPA1*0106-DPB1*0202 | HLA-DQA1*0104-DQB1*0612 |
| 388 | DRB1*1337 | HLA-DPA1*0106-DPB1*0301 | HLA-DQA1*0104-DQB1*0614 |
| 389 | DRB1*1338 | HLA-DPA1*0106-DPB1*0401 | HLA-DQA1*0104-DQB1*0615 |
| 390 | DRB1*1339 | HLA-DPA1*0106-DPB1*0402 | HLA-DQA1*0104-DQB1*0616 |
| 391 | DRB1*1341 | HLA-DPA1*0106-DPB1*0501 | HLA-DQA1*0104-DQB1*0617 |
| 392 | DRB1*1342 | HLA-DPA1*0106-DPB1*0601 | HLA-DQA1*0104-DQB1*0618 |
| 393 | DRB1*1343 | HLA-DPA1*0106-DPB1*0801 | HLA-DQA1*0104-DQB1*0619 |
| 394 | DRB1*1344 | HLA-DPA1*0106-DPB1*0901 | HLA-DQA1*0104-DQB1*0621 |
| 395 | DRB1*1346 | HLA-DPA1*0106-DPB1*0001 | HLA-DQA1*0104-DQB1*0622 |
| 396 | DRB1*1347 | HLA-DPA1*0106-DPB1*1001 | HLA-DQA1*0104-DQB1*0623 |
| 397 | DRB1*1348 | HLA-DPA1*0106-DPB1*0101 | HLA-DQA1*0104-DQB1*0624 |
| 398 | DRB1*1349 | HLA-DPA1*0106-DPB1*0201 | HLA-DQA1*0104-DQB1*0625 |
| 399 | DRB1*1350 | HLA-DPA1*0106-DPB1*0301 | HLA-DQA1*0104-DQB1*0627 |
| 400 | DRB1*1351 | HLA-DPA1*0106-DPB1*0401 | HLA-DQA1*0104-DQB1*0628 |
| 401 | DRB1*1352 | HLA-DPA1*0106-DPB1*0501 | HLA-DQA1*0104-DQB1*0629 |
| 402 | DRB1*1353 | HLA-DPA1*0106-DPB1*0601 | HLA-DQA1*0104-DQB1*0630 |
| 403 | DRB1*1354 | HLA-DPA1*0106-DPB1*0701 | HLA-DQA1*0104-DQB1*0631 |
| 404 | DRB1*1355 | HLA-DPA1*0106-DPB1*0801 | HLA-DQA1*0104-DQB1*0632 |
| 405 | DRB1*1356 | HLA-DPA1*0106-DPB1*0901 | HLA-DQA1*0104-DQB1*0633 |
| 406 | DRB1*1357 | HLA-DPA1*0106-DPB1*1001 | HLA-DQA1*0104-DQB1*0634 |
| 407 | DRB1*1358 | HLA-DPA1*0106-DPB1*1101 | HLA-DQA1*0104-DQB1*0635 |
| 408 | DRB1*1359 | HLA-DPA1*0106-DPB1*1101 | HLA-DQA1*0104-DQB1*0636 |
| 409 | DRB1*1360 | HLA-DPA1*0106-DPB1*1201 | HLA-DQA1*0104-DQB1*0637 |
| 410 | DRB1*1361 | HLA-DPA1*0106-DPB1*1301 | HLA-DQA1*0104-DQB1*0638 |
| 411 | DRB1*1362 | HLA-DPA1*0106-DPB1*1401 | HLA-DQA1*0104-DQB1*0639 |
| 412 | DRB1*1363 | HLA-DPA1*0106-DPB1*1501 | HLA-DQA1*0104-DQB1*0640 |
| 413 | DRB1*1364 | HLA-DPA1*0106-DPB1*1601 | HLA-DQA1*0104-DQB1*0641 |
| 414 | DRB1*1365 | HLA-DPA1*0106-DPB1*1701 | HLA-DQA1*0104-DQB1*0642 |
| 415 | DRB1*1366 | HLA-DPA1*0106-DPB1*1801 | HLA-DQA1*0104-DQB1*0643 |
| 416 | DRB1*1367 | HLA-DPA1*0106-DPB1*1901 | HLA-DQA1*0104-DQB1*0644 |
| 417 | DRB1*1368 | HLA-DPA1*0106-DPB1*2101 | HLA-DQA1*0105-DQB1*0201 |
| 418 | DRB1*1369 | HLA-DPA1*0106-DPB1*2201 | HLA-DQA1*0105-DQB1*0202 |
| 419 | DRB1*1370 | HLA-DPA1*0106-DPB1*2301 | HLA-DQA1*0105-DQB1*0203 |
| 420 | DRB1*1371 | HLA-DPA1*0106-DPB1*2401 | HLA-DQA1*0105-DQB1*0204 |
| 421 | DRB1*1372 | HLA-DPA1*0106-DPB1*2501 | HLA-DQA1*0105-DQB1*0205 |
| 422 | DRB1*1373 | HLA-DPA1*0106-DPB1*2601 | HLA-DQA1*0105-DQB1*0206 |
| 423 | DRB1*1374 | HLA-DPA1*0106-DPB1*2701 | HLA-DQA1*0105-DQB1*0301 |
| 424 | DRB1*1375 | HLA-DPA1*0106-DPB1*2801 | HLA-DQA1*0105-DQB1*0302 |
| 425 | DRB1*1376 | HLA-DPA1*0106-DPB1*2901 | HLA-DQA1*0105-DQB1*0303 |
| 426 | DRB1*1377 | HLA-DPA1*0106-DPB1*3001 | HLA-DQA1*0105-DQB1*0304 |
| 427 | DRB1*1378 | HLA-DPA1*0106-DPB1*1301 | HLA-DQA1*0105-DQB1*0305 |
| 428 | DRB1*1379 | HLA-DPA1*0106-DPB1*3101 | HLA-DQA1*0105-DQB1*0306 |
| 429 | DRB1*1380 | HLA-DPA1*0106-DPB1*3201 | HLA-DQA1*0105-DQB1*0307 |
| 430 | DRB1*1381 | HLA-DPA1*0106-DPB1*3301 | HLA-DQA1*0105-DQB1*0308 |
| 431 | DRB1*1382 | HLA-DPA1*0106-DPB1*3401 | HLA-DQA1*0105-DQB1*0309 |
| 432 | DRB1*1383 | HLA-DPA1*0106-DPB1*1401 | HLA-DQA1*0105-DQB1*0310 |
| 433 | DRB1*1384 | HLA-DPA1*0106-DPB1*1501 | HLA-DQA1*0105-DQB1*0311 |
| 434 | DRB1*1385 | HLA-DPA1*0106-DPB1*1601 | HLA-DQA1*0105-DQB1*0312 |
| 435 | DRB1*1386 | HLA-DPA1*0106-DPB1*1701 | HLA-DQA1*0105-DQB1*0313 |
| 436 | DRB1*1387 | HLA-DPA1*0106-DPB1*1801 | HLA-DQA1*0105-DQB1*0314 |
| 437 | DRB1*1388 | HLA-DPA1*0106-DPB1*1901 | HLA-DQA1*0105-DQB1*0315 |
| 438 | DRB1*1389 | HLA-DPA1*0106-DPB1*2001 | HLA-DQA1*0105-DQB1*0316 |
| 439 | DRB1*1390 | HLA-DPA1*0106-DPB1*2101 | HLA-DQA1*0105-DQB1*0317 |
| 440 | DRB1*1391 | HLA-DPA1*0106-DPB1*2201 | HLA-DQA1*0105-DQB1*0318 |
| 441 | DRB1*1392 | HLA-DPA1*0106-DPB1*2301 | HLA-DQA1*0105-DQB1*0319 |
| 442 | DRB1*1393 | HLA-DPA1*0106-DPB1*2401 | HLA-DQA1*0105-DQB1*0320 |
| 443 | DRB1*1394 | HLA-DPA1*0106-DPB1*2501 | HLA-DQA1*0105-DQB1*0321 |
| 444 | DRB1*1395 | HLA-DPA1*0106-DPB1*2601 | HLA-DQA1*0105-DQB1*0322 |
| 445 | DRB1*1396 | HLA-DPA1*0106-DPB1*2701 | HLA-DQA1*0105-DQB1*0323 |
| 446 | DRB1*1397 | HLA-DPA1*0106-DPB1*2801 | HLA-DQA1*0105-DQB1*0324 |
| 447 | DRB1*1398 | HLA-DPA1*0106-DPB1*2901 | HLA-DQA1*0105-DQB1*0325 |
| 448 | DRB1*1399 | HLA-DPA1*0106-DPB1*3001 | HLA-DQA1*0105-DQB1*0326 |
| 449 | DRB1*1401 | HLA-DPA1*0106-DPB1*3101 | HLA-DQA1*0105-DQB1*0327 |
| 450 | DRB1*1402 | HLA-DPA1*0106-DPB1*3201 | HLA-DQA1*0105-DQB1*0328 |
| 451 | DRB1*1403 | HLA-DPA1*0106-DPB1*3301 | HLA-DQA1*0105-DQB1*0329 |
| 452 | DRB1*1404 | HLA-DPA1*0106-DPB1*3401 | HLA-DQA1*0105-DQB1*0330 |
| 453 | DRB1*1405 | HLA-DPA1*0106-DPB1*3501 | HLA-DQA1*0105-DQB1*0331 |
| 454 | DRB1*1406 | HLA-DPA1*0106-DPB1*3601 | HLA-DQA1*0105-DQB1*0332 |
| 455 | DRB1*1407 | HLA-DPA1*0106-DPB1*3701 | HLA-DQA1*0105-DQB1*0333 |
| 456 | DRB1*1408 | HLA-DPA1*0106-DPB1*3801 | HLA-DQA1*0105-DQB1*0334 |
| 457 | DRB1*1409 | HLA-DPA1*0106-DPB1*3901 | HLA-DQA1*0105-DQB1*0335 |
| 458 | DRB1*1410 | HLA-DPA1*0106-DPB1*4001 | HLA-DQA1*0105-DQB1*0336 |
| 459 | DRB1*1411 | HLA-DPA1*0106-DPB1*4101 | HLA-DQA1*0105-DQB1*0337 |
| 460 | DRB1*1412 | HLA-DPA1*0106-DPB1*4401 | HLA-DQA1*0105-DQB1*0338 |
| 461 | DRB1*1413 | HLA-DPA1*0106-DPB1*4501 | HLA-DQA1*0105-DQB1*0401 |
| 462 | DRB1*1414 | HLA-DPA1*0106-DPB1*4601 | HLA-DQA1*0105-DQB1*0402 |
| 463 | DRB1*1415 | HLA-DPA1*0106-DPB1*4701 | HLA-DQA1*0105-DQB1*0403 |
| 464 | DRB1*1416 | HLA-DPA1*0106-DPB1*4801 | HLA-DQA1*0105-DQB1*0404 |
| 465 | DRB1*1417 | HLA-DPA1*0106-DPB1*4901 | HLA-DQA1*0105-DQB1*0405 |
| 466 | DRB1*1418 | HLA-DPA1*0106-DPB1*5001 | HLA-DQA1*0105-DQB1*0406 |
| 467 | DRB1*1419 | HLA-DPA1*0106-DPB1*5101 | HLA-DQA1*0105-DQB1*0407 |
| 468 | DRB1*1420 | HLA-DPA1*0106-DPB1*5201 | HLA-DQA1*0105-DQB1*0408 |
| 469 | DRB1*1421 | HLA-DPA1*0106-DPB1*5301 | HLA-DQA1*0105-DQB1*0501 |
| 470 | DRB1*1422 | HLA-DPA1*0106-DPB1*5401 | HLA-DQA1*0105-DQB1*0502 |
| 471 | DRB1*1423 | HLA-DPA1*0106-DPB1*5501 | HLA-DQA1*0105-DQB1*0503 |
| 472 | DRB1*1424 | HLA-DPA1*0106-DPB1*5601 | HLA-DQA1*0105-DQB1*0505 |
| 473 | DRB1*1425 | HLA-DPA1*0106-DPB1*5801 | HLA-DQA1*0105-DQB1*0506 |
| 474 | DRB1*1426 | HLA-DPA1*0106-DPB1*5901 | HLA-DQA1*0105-DQB1*0507 |
| 475 | DRB1*1427 | HLA-DPA1*0106-DPB1*6001 | HLA-DQA1*0105-DQB1*0508 |
| 476 | DRB1*1428 | HLA-DPA1*0106-DPB1*6201 | HLA-DQA1*0105-DQB1*0509 |
| 477 | DRB1*1429 | HLA-DPA1*0106-DPB1*6301 | HLA-DQA1*0105-DQB1*0510 |
| 478 | DRB1*1430 | HLA-DPA1*0106-DPB1*6501 | HLA-DQA1*0105-DQB1*0511 |
| 479 | DRB1*1431 | HLA-DPA1*0106-DPB1*6601 | HLA-DQA1*0105-DQB1*0512 |
| 480 | DRB1*1432 | HLA-DPA1*0106-DPB1*6701 | HLA-DQA1*0105-DQB1*0513 |
| 481 | DRB1*1433 | HLA-DPA1*0106-DPB1*6801 | HLA-DQA1*0105-DQB1*0514 |
| 482 | DRB1*1434 | HLA-DPA1*0106-DPB1*6901 | HLA-DQA1*0105-DQB1*0601 |
| 483 | DRB1*1435 | HLA-DPA1*0106-DPB1*7001 | HLA-DQA1*0105-DQB1*0602 |
| 484 | DRB1*1436 | HLA-DPA1*0106-DPB1*7101 | HLA-DQA1*0105-DQB1*0603 |
| 485 | DRB1*1437 | HLA-DPA1*0106-DPB1*7201 | HLA-DQA1*0105-DQB1*0604 |
| 486 | DRB1*1438 | HLA-DPA1*0106-DPB1*7301 | HLA-DQA1*0105-DQB1*0607 |
| 487 | DRB1*1439 | HLA-DPA1*0106-DPB1*7401 | HLA-DQA1*0105-DQB1*0608 |
| 488 | DRB1*1440 | HLA-DPA1*0106-DPB1*7501 | HLA-DQA1*0105-DQB1*0609 |
| 489 | DRB1*1441 | HLA-DPA1*0106-DPB1*7601 | HLA-DQA1*0105-DQB1*0610 |
| 490 | DRB1*1442 | HLA-DPA1*0106-DPB1*7701 | HLA-DQA1*0105-DQB1*0611 |
| 491 | DRB1*1443 | HLA-DPA1*0106-DPB1*7801 | HLA-DQA1*0105-DQB1*0612 |
| 492 | DRB1*1444 | HLA-DPA1*0106-DPB1*7901 | HLA-DQA1*0105-DQB1*0614 |
| 493 | DRB1*1445 | HLA-DPA1*0106-DPB1*8001 | HLA-DQA1*0105-DQB1*0615 |
| 494 | DRB1*1446 | HLA-DPA1*0106-DPB1*8101 | HLA-DQA1*0105-DQB1*0616 |
| 495 | DRB1*1447 | HLA-DPA1*0106-DPB1*8201 | HLA-DQA1*0105-DQB1*0617 |
| 496 | DRB1*1448 | HLA-DPA1*0106-DPB1*8301 | HLA-DQA1*0105-DQB1*0618 |
| 497 | DRB1*1449 | HLA-DPA1*0106-DPB1*8401 | HLA-DQA1*0105-DQB1*0619 |
| 498 | DRB1*1450 | HLA-DPA1*0106-DPB1*8501 | HLA-DQA1*0105-DQB1*0621 |
| 499 | DRB1*1451 | HLA-DPA1*0106-DPB1*8601 | HLA-DQA1*0105-DQB1*0622 |
| 500 | DRB1*1452 | HLA-DPA1*0106-DPB1*8701 | HLA-DQA1*0105-DQB1*0623 |
| 501 | DRB1*1453 | HLA-DPA1*0106-DPB1*8801 | HLA-DQA1*0105-DQB1*0624 |
| 502 | DRB1*1454 | HLA-DPA1*0106-DPB1*8901 | HLA-DQA1*0105-DQB1*0625 |
| 503 | DRB1*1455 | HLA-DPA1*0106-DPB1*9001 | HLA-DQA1*0105-DQB1*0627 |
| 504 | DRB1*1456 | HLA-DPA1*0106-DPB1*9101 | HLA-DQA1*0105-DQB1*0628 |
| 505 | DRB1*1457 | HLA-DPA1*0106-DPB1*9201 | HLA-DQA1*0105-DQB1*0629 |
| 506 | DRB1*1458 | HLA-DPA1*0106-DPB1*9301 | HLA-DQA1*0105-DQB1*0630 |
| 507 | DRB1*1459 | HLA-DPA1*0106-DPB1*9401 | HLA-DQA1*0105-DQB1*0631 |
| 508 | DRB1*1460 | HLA-DPA1*0106-DPB1*9501 | HLA-DQA1*0105-DQB1*0632 |
| 509 | DRB1*1461 | HLA-DPA1*0106-DPB1*9601 | HLA-DQA1*0105-DQB1*0633 |
| 510 | DRB1*1462 | HLA-DPA1*0106-DPB1*9701 | HLA-DQA1*0105-DQB1*0634 |
| 511 | DRB1*1463 | HLA-DPA1*0106-DPB1*9801 | HLA-DQA1*0105-DQB1*0635 |
| 512 | DRB1*1464 | HLA-DPA1*0106-DPB1*9901 | HLA-DQA1*0105-DQB1*0636 |
| 513 | DRB1*1465 | HLA-DPA1*0107-DPB1*0101 | HLA-DQA1*0105-DQB1*0637 |
| 514 | DRB1*1467 | HLA-DPA1*0107-DPB1*0201 | HLA-DQA1*0105-DQB1*0638 |
| 515 | DRB1*1468 | HLA-DPA1*0107-DPB1*0202 | HLA-DQA1*0105-DQB1*0639 |
| 516 | DRB1*1469 | HLA-DPA1*0107-DPB1*0301 | HLA-DQA1*0105-DQB1*0640 |
| 517 | DRB1*1470 | HLA-DPA1*0107-DPB1*0401 | HLA-DQA1*0105-DQB1*0641 |
| 518 | DRB1*1471 | HLA-DPA1*0107-DPB1*0402 | HLA-DQA1*0105-DQB1*0642 |
| 519 | DRB1*1472 | HLA-DPA1*0107-DPB1*0501 | HLA-DQA1*0105-DQB1*0643 |
| 520 | DRB1*1473 | HLA-DPA1*0107-DPB1*0601 | HLA-DQA1*0105-DQB1*0644 |
| 521 | DRB1*1474 | HLA-DPA1*0107-DPB1*0801 | HLA-DQA1*0106-DQB1*0201 |
| 522 | DRB1*1475 | HLA-DPA1*0107-DPB1*0901 | HLA-DQA1*0106-DQB1*0202 |
| 523 | DRB1*1476 | HLA-DPA1*0107-DPB1*0001 | HLA-DQA1*0106-DQB1*0203 |
| 524 | DRB1*1477 | HLA-DPA1*0107-DPB1*1001 | HLA-DQA1*0106-DQB1*0204 |
| 525 | DRB1*1478 | HLA-DPA1*0107-DPB1*0101 | HLA-DQA1*0106-DQB1*0205 |
| 526 | DRB1*1479 | HLA-DPA1*0107-DPB1*0201 | HLA-DQA1*0106-DQB1*0206 |
| 527 | DRB1*1480 | HLA-DPA1*0107-DPB1*0301 | HLA-DQA1*0106-DQB1*0301 |
| 528 | DRB1*1481 | HLA-DPA1*0107-DPB1*0401 | HLA-DQA1*0106-DQB1*0302 |
| 529 | DRB1*1482 | HLA-DPA1*0107-DPB1*0501 | HLA-DQA1*0106-DQB1*0303 |
| 530 | DRB1*1483 | HLA-DPA1*0107-DPB1*0601 | HLA-DQA1*0106-DQB1*0304 |
| 531 | DRB1*1484 | HLA-DPA1*0107-DPB1*0701 | HLA-DQA1*0106-DQB1*0305 |
| 532 | DRB1*1485 | HLA-DPA1*0107-DPB1*0801 | HLA-DQA1*0106-DQB1*0306 |
| 533 | DRB1*1486 | HLA-DPA1*0107-DPB1*0901 | HLA-DQA1*0106-DQB1*0307 |
| 534 | DRB1*1487 | HLA-DPA1*0107-DPB1*1001 | HLA-DQA1*0106-DQB1*0308 |
| 535 | DRB1*1488 | HLA-DPA1*0107-DPB1*1101 | HLA-DQA1*0106-DQB1*0309 |
| 536 | DRB1*1489 | HLA-DPA1*0107-DPB1*1101 | HLA-DQA1*0106-DQB1*0310 |
| 537 | DRB1*1490 | HLA-DPA1*0107-DPB1*1201 | HLA-DQA1*0106-DQB1*0311 |
| 538 | DRB1*1491 | HLA-DPA1*0107-DPB1*1301 | HLA-DQA1*0106-DQB1*0312 |
| 539 | DRB1*1493 | HLA-DPA1*0107-DPB1*1401 | HLA-DQA1*0106-DQB1*0313 |
| 540 | DRB1*1494 | HLA-DPA1*0107-DPB1*1501 | HLA-DQA1*0106-DQB1*0314 |
| 541 | DRB1*1495 | HLA-DPA1*0107-DPB1*1601 | HLA-DQA1*0106-DQB1*0315 |
| 542 | DRB1*1496 | HLA-DPA1*0107-DPB1*1701 | HLA-DQA1*0106-DQB1*0316 |
| 543 | DRB1*1497 | HLA-DPA1*0107-DPB1*1801 | HLA-DQA1*0106-DQB1*0317 |
| 544 | DRB1*1498 | HLA-DPA1*0107-DPB1*1901 | HLA-DQA1*0106-DQB1*0318 |
| 545 | DRB1*1499 | HLA-DPA1*0107-DPB1*2101 | HLA-DQA1*0106-DQB1*0319 |
| 546 | DRB1*1501 | HLA-DPA1*0107-DPB1*2201 | HLA-DQA1*0106-DQB1*0320 |
| 547 | DRB1*1502 | HLA-DPA1*0107-DPB1*2301 | HLA-DQA1*0106-DQB1*0321 |
| 548 | DRB1*1503 | HLA-DPA1*0107-DPB1*2401 | HLA-DQA1*0106-DQB1*0322 |
| 549 | DRB1*1504 | HLA-DPA1*0107-DPB1*2501 | HLA-DQA1*0106-DQB1*0323 |
| 550 | DRB1*1505 | HLA-DPA1*0107-DPB1*2601 | HLA-DQA1*0106-DQB1*0324 |
| 551 | DRB1*1506 | HLA-DPA1*0107-DPB1*2701 | HLA-DQA1*0106-DQB1*0325 |
| 552 | DRB1*1507 | HLA-DPA1*0107-DPB1*2801 | HLA-DQA1*0106-DQB1*0326 |
| 553 | DRB1*1508 | HLA-DPA1*0107-DPB1*2901 | HLA-DQA1*0106-DQB1*0327 |
| 554 | DRB1*1509 | HLA-DPA1*0107-DPB1*3001 | HLA-DQA1*0106-DQB1*0328 |
| 555 | DRB1*1510 | HLA-DPA1*0107-DPB1*1301 | HLA-DQA1*0106-DQB1*0329 |
| 556 | DRB1*1511 | HLA-DPA1*0107-DPB1*3101 | HLA-DQA1*0106-DQB1*0330 |
| 557 | DRB1*1512 | HLA-DPA1*0107-DPB1*3201 | HLA-DQA1*0106-DQB1*0331 |
| 558 | DRB1*1513 | HLA-DPA1*0107-DPB1*3301 | HLA-DQA1*0106-DQB1*0332 |
| 559 | DRB1*1514 | HLA-DPA1*0107-DPB1*3401 | HLA-DQA1*0106-DQB1*0333 |
| 560 | DRB1*1515 | HLA-DPA1*0107-DPB1*1401 | HLA-DQA1*0106-DQB1*0334 |
| 561 | DRB1*1516 | HLA-DPA1*0107-DPB1*1501 | HLA-DQA1*0106-DQB1*0335 |
| 562 | DRB1*1518 | HLA-DPA1*0107-DPB1*1601 | HLA-DQA1*0106-DQB1*0336 |
| 563 | DRB1*1519 | HLA-DPA1*0107-DPB1*1701 | HLA-DQA1*0106-DQB1*0337 |
| 564 | DRB1*1520 | HLA-DPA1*0107-DPB1*1801 | HLA-DQA1*0106-DQB1*0338 |
| 565 | DRB1*1521 | HLA-DPA1*0107-DPB1*1901 | HLA-DQA1*0106-DQB1*0401 |
| 566 | DRB1*1522 | HLA-DPA1*0107-DPB1*2001 | HLA-DQA1*0106-DQB1*0402 |
| 567 | DRB1*1523 | HLA-DPA1*0107-DPB1*2101 | HLA-DQA1*0106-DQB1*0403 |
| 568 | DRB1*1524 | HLA-DPA1*0107-DPB1*2201 | HLA-DQA1*0106-DQB1*0404 |
| 569 | DRB1*1525 | HLA-DPA1*0107-DPB1*2301 | HLA-DQA1*0106-DQB1*0405 |
| 570 | DRB1*1526 | HLA-DPA1*0107-DPB1*2401 | HLA-DQA1*0106-DQB1*0406 |
| 571 | DRB1*1527 | HLA-DPA1*0107-DPB1*2501 | HLA-DQA1*0106-DQB1*0407 |
| 572 | DRB1*1528 | HLA-DPA1*0107-DPB1*2601 | HLA-DQA1*0106-DQB1*0408 |
| 573 | DRB1*1529 | HLA-DPA1*0107-DPB1*2701 | HLA-DQA1*0106-DQB1*0501 |
| 574 | DRB1*1530 | HLA-DPA1*0107-DPB1*2801 | HLA-DQA1*0106-DQB1*0502 |
| 575 | DRB1*1531 | HLA-DPA1*0107-DPB1*2901 | HLA-DQA1*0106-DQB1*0503 |
| 576 | DRB1*1532 | HLA-DPA1*0107-DPB1*3001 | HLA-DQA1*0106-DQB1*0505 |
| 577 | DRB1*1533 | HLA-DPA1*0107-DPB1*3101 | HLA-DQA1*0106-DQB1*0506 |
| 578 | DRB1*1534 | HLA-DPA1*0107-DPB1*3201 | HLA-DQA1*0106-DQB1*0507 |
| 579 | DRB1*1535 | HLA-DPA1*0107-DPB1*3301 | HLA-DQA1*0106-DQB1*0508 |
| 580 | DRB1*1536 | HLA-DPA1*0107-DPB1*3401 | HLA-DQA1*0106-DQB1*0509 |
| 581 | DRB1*1537 | HLA-DPA1*0107-DPB1*3501 | HLA-DQA1*0106-DQB1*0510 |
| 582 | DRB1*1538 | HLA-DPA1*0107-DPB1*3601 | HLA-DQA1*0106-DQB1*0511 |
| 583 | DRB1*1539 | HLA-DPA1*0107-DPB1*3701 | HLA-DQA1*0106-DQB1*0512 |
| 584 | DRB1*1540 | HLA-DPA1*0107-DPB1*3801 | HLA-DQA1*0106-DQB1*0513 |
| 585 | DRB1*1541 | HLA-DPA1*0107-DPB1*3901 | HLA-DQA1*0106-DQB1*0514 |
| 586 | DRB1*1542 | HLA-DPA1*0107-DPB1*4001 | HLA-DQA1*0106-DQB1*0601 |
| 587 | DRB1*1543 | HLA-DPA1*0107-DPB1*4101 | HLA-DQA1*0106-DQB1*0602 |
| 588 | DRB1*1544 | HLA-DPA1*0107-DPB1*4401 | HLA-DQA1*0106-DQB1*0603 |
| 589 | DRB1*1545 | HLA-DPA1*0107-DPB1*4501 | HLA-DQA1*0106-DQB1*0604 |
| 590 | DRB1*1546 | HLA-DPA1*0107-DPB1*4601 | HLA-DQA1*0106-DQB1*0607 |
| 591 | DRB1*1547 | HLA-DPA1*0107-DPB1*4701 | HLA-DQA1*0106-DQB1*0608 |
| 592 | DRB1*1548 | HLA-DPA1*0107-DPB1*4801 | HLA-DQA1*0106-DQB1*0609 |
| 593 | DRB1*1549 | HLA-DPA1*0107-DPB1*4901 | HLA-DQA1*0106-DQB1*0610 |
| 594 | DRB1*1601 | HLA-DPA1*0107-DPB1*5001 | HLA-DQA1*0106-DQB1*0611 |
| 595 | DRB1*1602 | HLA-DPA1*0107-DPB1*5101 | HLA-DQA1*0106-DQB1*0612 |
| 596 | DRB1*1603 | HLA-DPA1*0107-DPB1*5201 | HLA-DQA1*0106-DQB1*0614 |
| 597 | DRB1*1604 | HLA-DPA1*0107-DPB1*5301 | HLA-DQA1*0106-DQB1*0615 |
| 598 | DRB1*1605 | HLA-DPA1*0107-DPB1*5401 | HLA-DQA1*0106-DQB1*0616 |
| 599 | DRB1*1607 | HLA-DPA1*0107-DPB1*5501 | HLA-DQA1*0106-DQB1*0617 |
| 600 | DRB1*1608 | HLA-DPA1*0107-DPB1*5601 | HLA-DQA1*0106-DQB1*0618 |
| 601 | DRB1*1609 | HLA-DPA1*0107-DPB1*5801 | HLA-DQA1*0106-DQB1*0619 |
| 602 | DRB1*1610 | HLA-DPA1*0107-DPB1*5901 | HLA-DQA1*0106-DQB1*0621 |
| 603 | DRB1*1611 | HLA-DPA1*0107-DPB1*6001 | HLA-DQA1*0106-DQB1*0622 |
| 604 | DRB1*1612 | HLA-DPA1*0107-DPB1*6201 | HLA-DQA1*0106-DQB1*0623 |
| 605 | DRB1*1614 | HLA-DPA1*0107-DPB1*6301 | HLA-DQA1*0106-DQB1*0624 |
| 606 | DRB1*1615 | HLA-DPA1*0107-DPB1*6501 | HLA-DQA1*0106-DQB1*0625 |
| 607 | DRB1*1616 | HLA-DPA1*0107-DPB1*6601 | HLA-DQA1*0106-DQB1*0627 |
| 608 | DRB3*0101 | HLA-DPA1*0107-DPB1*6701 | HLA-DQA1*0106-DQB1*0628 |
| 609 | DRB3*0104 | HLA-DPA1*0107-DPB1*6801 | HLA-DQA1*0106-DQB1*0629 |
| 610 | DRB3*0105 | HLA-DPA1*0107-DPB1*6901 | HLA-DQA1*0106-DQB1*0630 |
| 611 | DRB3*0108 | HLA-DPA1*0107-DPB1*7001 | HLA-DQA1*0106-DQB1*0631 |
| 612 | DRB3*0109 | HLA-DPA1*0107-DPB1*7101 | HLA-DQA1*0106-DQB1*0632 |
| 613 | DRB3*0111 | HLA-DPA1*0107-DPB1*7201 | HLA-DQA1*0106-DQB1*0633 |
| 614 | DRB3*0112 | HLA-DPA1*0107-DPB1*7301 | HLA-DQA1*0106-DQB1*0634 |
| 615 | DRB3*0113 | HLA-DPA1*0107-DPB1*7401 | HLA-DQA1*0106-DQB1*0635 |
| 616 | DRB3*0114 | HLA-DPA1*0107-DPB1*7501 | HLA-DQA1*0106-DQB1*0636 |
| 617 | DRB3*0201 | HLA-DPA1*0107-DPB1*7601 | HLA-DQA1*0106-DQB1*0637 |
| 618 | DRB3*0202 | HLA-DPA1*0107-DPB1*7701 | HLA-DQA1*0106-DQB1*0638 |
| 619 | DRB3*0204 | HLA-DPA1*0107-DPB1*7801 | HLA-DQA1*0106-DQB1*0639 |
| 620 | DRB3*0205 | HLA-DPA1*0107-DPB1*7901 | HLA-DQA1*0106-DQB1*0640 |
| 621 | DRB3*0209 | HLA-DPA1*0107-DPB1*8001 | HLA-DQA1*0106-DQB1*0641 |
| 622 | DRB3*0210 | HLA-DPA1*0107-DPB1*8101 | HLA-DQA1*0106-DQB1*0642 |
| 623 | DRB3*0211 | HLA-DPA1*0107-DPB1*8201 | HLA-DQA1*0106-DQB1*0643 |
| 624 | DRB3*0212 | HLA-DPA1*0107-DPB1*8301 | HLA-DQA1*0106-DQB1*0644 |
| 625 | DRB3*0213 | HLA-DPA1*0107-DPB1*8401 | HLA-DQA1*0107-DQB1*0201 |
| 626 | DRB3*0214 | HLA-DPA1*0107-DPB1*8501 | HLA-DQA1*0107-DQB1*0202 |
| 627 | DRB3*0215 | HLA-DPA1*0107-DPB1*8601 | HLA-DQA1*0107-DQB1*0203 |
| 628 | DRB3*0216 | HLA-DPA1*0107-DPB1*8701 | HLA-DQA1*0107-DQB1*0204 |
| 629 | DRB3*0217 | HLA-DPA1*0107-DPB1*8801 | HLA-DQA1*0107-DQB1*0205 |
| 630 | DRB3*0218 | HLA-DPA1*0107-DPB1*8901 | HLA-DQA1*0107-DQB1*0206 |
| 631 | DRB3*0219 | HLA-DPA1*0107-DPB1*9001 | HLA-DQA1*0107-DQB1*0301 |
| 632 | DRB3*0220 | HLA-DPA1*0107-DPB1*9101 | HLA-DQA1*0107-DQB1*0302 |
| 633 | DRB3*0221 | HLA-DPA1*0107-DPB1*9201 | HLA-DQA1*0107-DQB1*0303 |
| 634 | DRB3*0222 | HLA-DPA1*0107-DPB1*9301 | HLA-DQA1*0107-DQB1*0304 |
| 635 | DRB3*0223 | HLA-DPA1*0107-DPB1*9401 | HLA-DQA1*0107-DQB1*0305 |
| 636 | DRB3*0224 | HLA-DPA1*0107-DPB1*9501 | HLA-DQA1*0107-DQB1*0306 |
| 637 | DRB3*0225 | HLA-DPA1*0107-DPB1*9601 | HLA-DQA1*0107-DQB1*0307 |
| 638 | DRB3*0301 | HLA-DPA1*0107-DPB1*9701 | HLA-DQA1*0107-DQB1*0308 |
| 639 | DRB3*0303 | HLA-DPA1*0107-DPB1*9801 | HLA-DQA1*0107-DQB1*0309 |
| 640 | DRB4*0101 | HLA-DPA1*0107-DPB1*9901 | HLA-DQA1*0107-DQB1*0310 |
| 641 | DRB4*0103 | HLA-DPA1*0108-DPB1*0101 | HLA-DQA1*0107-DQB1*0311 |
| 642 | DRB4*0104 | HLA-DPA1*0108-DPB1*0201 | HLA-DQA1*0107-DQB1*0312 |
| 643 | DRB4*0106 | HLA-DPA1*0108-DPB1*0202 | HLA-DQA1*0107-DQB1*0313 |
| 644 | DRB4*0107 | HLA-DPA1*0108-DPB1*0301 | HLA-DQA1*0107-DQB1*0314 |
| 645 | DRB4*0108 | HLA-DPA1*0108-DPB1*0401 | HLA-DQA1*0107-DQB1*0315 |
| 646 | DRB5*0101 | HLA-DPA1*0108-DPB1*0402 | HLA-DQA1*0107-DQB1*0316 |
| 647 | DRB5*0102 | HLA-DPA1*0108-DPB1*0501 | HLA-DQA1*0107-DQB1*0317 |
| 648 | DRB5*0103 | HLA-DPA1*0108-DPB1*0601 | HLA-DQA1*0107-DQB1*0318 |
| 649 | DRB5*0104 | HLA-DPA1*0108-DPB1*0801 | HLA-DQA1*0107-DQB1*0319 |
| 650 | DRB5*0105 | HLA-DPA1*0108-DPB1*0901 | HLA-DQA1*0107-DQB1*0320 |
| 651 | DRB5*0106 | HLA-DPA1*0108-DPB1*0001 | HLA-DQA1*0107-DQB1*0321 |
| 652 | DRB5*0108N | HLA-DPA1*0108-DPB1*1001 | HLA-DQA1*0107-DQB1*0322 |
| 653 | DRB5*0111 | HLA-DPA1*0108-DPB1*0101 | HLA-DQA1*0107-DQB1*0323 |
| 654 | DRB5*0112 | HLA-DPA1*0108-DPB1*0201 | HLA-DQA1*0107-DQB1*0324 |
| 655 | DRB5*0113 | HLA-DPA1*0108-DPB1*0301 | HLA-DQA1*0107-DQB1*0325 |
| 656 | DRB5*0114 | HLA-DPA1*0108-DPB1*0401 | HLA-DQA1*0107-DQB1*0326 |
| 657 | DRB5*0202 | HLA-DPA1*0108-DPB1*0501 | HLA-DQA1*0107-DQB1*0327 |
| 658 | DRB5*0203 | HLA-DPA1*0108-DPB1*0601 | HLA-DQA1*0107-DQB1*0328 |
| 659 | DRB5*0204 | HLA-DPA1*0108-DPB1*0701 | HLA-DQA1*0107-DQB1*0329 |
| 660 | DRB5*0205 | HLA-DPA1*0108-DPB1*0801 | HLA-DQA1*0107-DQB1*0330 |
| 661 |  | HLA-DPA1*0108-DPB1*0901 | HLA-DQA1*0107-DQB1*0331 |
| 662 |  | HLA-DPA1*0108-DPB1*1001 | HLA-DQA1*0107-DQB1*0332 |
| 663 |  | HLA-DPA1*0108-DPB1*1101 | HLA-DQA1*0107-DQB1*0333 |
| 664 |  | HLA-DPA1*0108-DPB1*1101 | HLA-DQA1*0107-DQB1*0334 |
| 665 |  | HLA-DPA1*0108-DPB1*1201 | HLA-DQA1*0107-DQB1*0335 |
| 666 |  | HLA-DPA1*0108-DPB1*1301 | HLA-DQA1*0107-DQB1*0336 |
| 667 |  | HLA-DPA1*0108-DPB1*1401 | HLA-DQA1*0107-DQB1*0337 |
| 668 |  | HLA-DPA1*0108-DPB1*1501 | HLA-DQA1*0107-DQB1*0338 |
| 669 |  | HLA-DPA1*0108-DPB1*1601 | HLA-DQA1*0107-DQB1*0401 |
| 670 |  | HLA-DPA1*0108-DPB1*1701 | HLA-DQA1*0107-DQB1*0402 |
| 671 |  | HLA-DPA1*0108-DPB1*1801 | HLA-DQA1*0107-DQB1*0403 |
| 672 |  | HLA-DPA1*0108-DPB1*1901 | HLA-DQA1*0107-DQB1*0404 |
| 673 |  | HLA-DPA1*0108-DPB1*2101 | HLA-DQA1*0107-DQB1*0405 |
| 674 |  | HLA-DPA1*0108-DPB1*2201 | HLA-DQA1*0107-DQB1*0406 |
| 675 |  | HLA-DPA1*0108-DPB1*2301 | HLA-DQA1*0107-DQB1*0407 |
| 676 |  | HLA-DPA1*0108-DPB1*2401 | HLA-DQA1*0107-DQB1*0408 |
| 677 |  | HLA-DPA1*0108-DPB1*2501 | HLA-DQA1*0107-DQB1*0501 |
| 678 |  | HLA-DPA1*0108-DPB1*2601 | HLA-DQA1*0107-DQB1*0502 |
| 679 |  | HLA-DPA1*0108-DPB1*2701 | HLA-DQA1*0107-DQB1*0503 |
| 680 |  | HLA-DPA1*0108-DPB1*2801 | HLA-DQA1*0107-DQB1*0505 |
| 681 |  | HLA-DPA1*0108-DPB1*2901 | HLA-DQA1*0107-DQB1*0506 |
| 682 |  | HLA-DPA1*0108-DPB1*3001 | HLA-DQA1*0107-DQB1*0507 |
| 683 |  | HLA-DPA1*0108-DPB1*1301 | HLA-DQA1*0107-DQB1*0508 |
| 684 |  | HLA-DPA1*0108-DPB1*3101 | HLA-DQA1*0107-DQB1*0509 |
| 685 |  | HLA-DPA1*0108-DPB1*3201 | HLA-DQA1*0107-DQB1*0510 |
| 686 |  | HLA-DPA1*0108-DPB1*3301 | HLA-DQA1*0107-DQB1*0511 |
| 687 |  | HLA-DPA1*0108-DPB1*3401 | HLA-DQA1*0107-DQB1*0512 |
| 688 |  | HLA-DPA1*0108-DPB1*1401 | HLA-DQA1*0107-DQB1*0513 |
| 689 |  | HLA-DPA1*0108-DPB1*1501 | HLA-DQA1*0107-DQB1*0514 |
| 690 |  | HLA-DPA1*0108-DPB1*1601 | HLA-DQA1*0107-DQB1*0601 |
| 691 |  | HLA-DPA1*0108-DPB1*1701 | HLA-DQA1*0107-DQB1*0602 |
| 692 |  | HLA-DPA1*0108-DPB1*1801 | HLA-DQA1*0107-DQB1*0603 |
| 693 |  | HLA-DPA1*0108-DPB1*1901 | HLA-DQA1*0107-DQB1*0604 |
| 694 |  | HLA-DPA1*0108-DPB1*2001 | HLA-DQA1*0107-DQB1*0607 |
| 695 |  | HLA-DPA1*0108-DPB1*2101 | HLA-DQA1*0107-DQB1*0608 |
| 696 |  | HLA-DPA1*0108-DPB1*2201 | HLA-DQA1*0107-DQB1*0609 |
| 697 |  | HLA-DPA1*0108-DPB1*2301 | HLA-DQA1*0107-DQB1*0610 |
| 698 |  | HLA-DPA1*0108-DPB1*2401 | HLA-DQA1*0107-DQB1*0611 |
| 699 |  | HLA-DPA1*0108-DPB1*2501 | HLA-DQA1*0107-DQB1*0612 |
| 700 |  | HLA-DPA1*0108-DPB1*2601 | HLA-DQA1*0107-DQB1*0614 |
| 701 |  | HLA-DPA1*0108-DPB1*2701 | HLA-DQA1*0107-DQB1*0615 |
| 702 |  | HLA-DPA1*0108-DPB1*2801 | HLA-DQA1*0107-DQB1*0616 |
| 703 |  | HLA-DPA1*0108-DPB1*2901 | HLA-DQA1*0107-DQB1*0617 |
| 704 |  | HLA-DPA1*0108-DPB1*3001 | HLA-DQA1*0107-DQB1*0618 |
| 705 |  | HLA-DPA1*0108-DPB1*3101 | HLA-DQA1*0107-DQB1*0619 |
| 706 |  | HLA-DPA1*0108-DPB1*3201 | HLA-DQA1*0107-DQB1*0621 |
| 707 |  | HLA-DPA1*0108-DPB1*3301 | HLA-DQA1*0107-DQB1*0622 |
| 708 |  | HLA-DPA1*0108-DPB1*3401 | HLA-DQA1*0107-DQB1*0623 |
| 709 |  | HLA-DPA1*0108-DPB1*3501 | HLA-DQA1*0107-DQB1*0624 |
| 710 |  | HLA-DPA1*0108-DPB1*3601 | HLA-DQA1*0107-DQB1*0625 |
| 711 |  | HLA-DPA1*0108-DPB1*3701 | HLA-DQA1*0107-DQB1*0627 |
| 712 |  | HLA-DPA1*0108-DPB1*3801 | HLA-DQA1*0107-DQB1*0628 |
| 713 |  | HLA-DPA1*0108-DPB1*3901 | HLA-DQA1*0107-DQB1*0629 |
| 714 |  | HLA-DPA1*0108-DPB1*4001 | HLA-DQA1*0107-DQB1*0630 |
| 715 |  | HLA-DPA1*0108-DPB1*4101 | HLA-DQA1*0107-DQB1*0631 |
| 716 |  | HLA-DPA1*0108-DPB1*4401 | HLA-DQA1*0107-DQB1*0632 |
| 717 |  | HLA-DPA1*0108-DPB1*4501 | HLA-DQA1*0107-DQB1*0633 |
| 718 |  | HLA-DPA1*0108-DPB1*4601 | HLA-DQA1*0107-DQB1*0634 |
| 719 |  | HLA-DPA1*0108-DPB1*4701 | HLA-DQA1*0107-DQB1*0635 |
| 720 |  | HLA-DPA1*0108-DPB1*4801 | HLA-DQA1*0107-DQB1*0636 |
| 721 |  | HLA-DPA1*0108-DPB1*4901 | HLA-DQA1*0107-DQB1*0637 |
| 722 |  | HLA-DPA1*0108-DPB1*5001 | HLA-DQA1*0107-DQB1*0638 |
| 723 |  | HLA-DPA1*0108-DPB1*5101 | HLA-DQA1*0107-DQB1*0639 |
| 724 |  | HLA-DPA1*0108-DPB1*5201 | HLA-DQA1*0107-DQB1*0640 |
| 725 |  | HLA-DPA1*0108-DPB1*5301 | HLA-DQA1*0107-DQB1*0641 |
| 726 |  | HLA-DPA1*0108-DPB1*5401 | HLA-DQA1*0107-DQB1*0642 |
| 727 |  | HLA-DPA1*0108-DPB1*5501 | HLA-DQA1*0107-DQB1*0643 |
| 728 |  | HLA-DPA1*0108-DPB1*5601 | HLA-DQA1*0107-DQB1*0644 |
| 729 |  | HLA-DPA1*0108-DPB1*5801 | HLA-DQA1*0108-DQB1*0201 |
| 730 |  | HLA-DPA1*0108-DPB1*5901 | HLA-DQA1*0108-DQB1*0202 |
| 731 |  | HLA-DPA1*0108-DPB1*6001 | HLA-DQA1*0108-DQB1*0203 |
| 732 |  | HLA-DPA1*0108-DPB1*6201 | HLA-DQA1*0108-DQB1*0204 |
| 733 |  | HLA-DPA1*0108-DPB1*6301 | HLA-DQA1*0108-DQB1*0205 |
| 734 |  | HLA-DPA1*0108-DPB1*6501 | HLA-DQA1*0108-DQB1*0206 |
| 735 |  | HLA-DPA1*0108-DPB1*6601 | HLA-DQA1*0108-DQB1*0301 |
| 736 |  | HLA-DPA1*0108-DPB1*6701 | HLA-DQA1*0108-DQB1*0302 |
| 737 |  | HLA-DPA1*0108-DPB1*6801 | HLA-DQA1*0108-DQB1*0303 |
| 738 |  | HLA-DPA1*0108-DPB1*6901 | HLA-DQA1*0108-DQB1*0304 |
| 739 |  | HLA-DPA1*0108-DPB1*7001 | HLA-DQA1*0108-DQB1*0305 |
| 740 |  | HLA-DPA1*0108-DPB1*7101 | HLA-DQA1*0108-DQB1*0306 |
| 741 |  | HLA-DPA1*0108-DPB1*7201 | HLA-DQA1*0108-DQB1*0307 |
| 742 |  | HLA-DPA1*0108-DPB1*7301 | HLA-DQA1*0108-DQB1*0308 |
| 743 |  | HLA-DPA1*0108-DPB1*7401 | HLA-DQA1*0108-DQB1*0309 |
| 744 |  | HLA-DPA1*0108-DPB1*7501 | HLA-DQA1*0108-DQB1*0310 |
| 745 |  | HLA-DPA1*0108-DPB1*7601 | HLA-DQA1*0108-DQB1*0311 |
| 746 |  | HLA-DPA1*0108-DPB1*7701 | HLA-DQA1*0108-DQB1*0312 |
| 747 |  | HLA-DPA1*0108-DPB1*7801 | HLA-DQA1*0108-DQB1*0313 |
| 748 |  | HLA-DPA1*0108-DPB1*7901 | HLA-DQA1*0108-DQB1*0314 |
| 749 |  | HLA-DPA1*0108-DPB1*8001 | HLA-DQA1*0108-DQB1*0315 |
| 750 |  | HLA-DPA1*0108-DPB1*8101 | HLA-DQA1*0108-DQB1*0316 |
| 751 |  | HLA-DPA1*0108-DPB1*8201 | HLA-DQA1*0108-DQB1*0317 |
| 752 |  | HLA-DPA1*0108-DPB1*8301 | HLA-DQA1*0108-DQB1*0318 |
| 753 |  | HLA-DPA1*0108-DPB1*8401 | HLA-DQA1*0108-DQB1*0319 |
| 754 |  | HLA-DPA1*0108-DPB1*8501 | HLA-DQA1*0108-DQB1*0320 |
| 755 |  | HLA-DPA1*0108-DPB1*8601 | HLA-DQA1*0108-DQB1*0321 |
| 756 |  | HLA-DPA1*0108-DPB1*8701 | HLA-DQA1*0108-DQB1*0322 |
| 757 |  | HLA-DPA1*0108-DPB1*8801 | HLA-DQA1*0108-DQB1*0323 |
| 758 |  | HLA-DPA1*0108-DPB1*8901 | HLA-DQA1*0108-DQB1*0324 |
| 759 |  | HLA-DPA1*0108-DPB1*9001 | HLA-DQA1*0108-DQB1*0325 |
| 760 |  | HLA-DPA1*0108-DPB1*9101 | HLA-DQA1*0108-DQB1*0326 |
| 761 |  | HLA-DPA1*0108-DPB1*9201 | HLA-DQA1*0108-DQB1*0327 |
| 762 |  | HLA-DPA1*0108-DPB1*9301 | HLA-DQA1*0108-DQB1*0328 |
| 763 |  | HLA-DPA1*0108-DPB1*9401 | HLA-DQA1*0108-DQB1*0329 |
| 764 |  | HLA-DPA1*0108-DPB1*9501 | HLA-DQA1*0108-DQB1*0330 |
| 765 |  | HLA-DPA1*0108-DPB1*9601 | HLA-DQA1*0108-DQB1*0331 |
| 766 |  | HLA-DPA1*0108-DPB1*9701 | HLA-DQA1*0108-DQB1*0332 |
| 767 |  | HLA-DPA1*0108-DPB1*9801 | HLA-DQA1*0108-DQB1*0333 |
| 768 |  | HLA-DPA1*0108-DPB1*9901 | HLA-DQA1*0108-DQB1*0334 |
| 769 |  | HLA-DPA1*0109-DPB1*0101 | HLA-DQA1*0108-DQB1*0335 |
| 770 |  | HLA-DPA1*0109-DPB1*0201 | HLA-DQA1*0108-DQB1*0336 |
| 771 |  | HLA-DPA1*0109-DPB1*0202 | HLA-DQA1*0108-DQB1*0337 |
| 772 |  | HLA-DPA1*0109-DPB1*0301 | HLA-DQA1*0108-DQB1*0338 |
| 773 |  | HLA-DPA1*0109-DPB1*0401 | HLA-DQA1*0108-DQB1*0401 |
| 774 |  | HLA-DPA1*0109-DPB1*0402 | HLA-DQA1*0108-DQB1*0402 |
| 775 |  | HLA-DPA1*0109-DPB1*0501 | HLA-DQA1*0108-DQB1*0403 |
| 776 |  | HLA-DPA1*0109-DPB1*0601 | HLA-DQA1*0108-DQB1*0404 |
| 777 |  | HLA-DPA1*0109-DPB1*0801 | HLA-DQA1*0108-DQB1*0405 |
| 778 |  | HLA-DPA1*0109-DPB1*0901 | HLA-DQA1*0108-DQB1*0406 |
| 779 |  | HLA-DPA1*0109-DPB1*0001 | HLA-DQA1*0108-DQB1*0407 |
| 780 |  | HLA-DPA1*0109-DPB1*1001 | HLA-DQA1*0108-DQB1*0408 |
| 781 |  | HLA-DPA1*0109-DPB1*0101 | HLA-DQA1*0108-DQB1*0501 |
| 782 |  | HLA-DPA1*0109-DPB1*0201 | HLA-DQA1*0108-DQB1*0502 |
| 783 |  | HLA-DPA1*0109-DPB1*0301 | HLA-DQA1*0108-DQB1*0503 |
| 784 |  | HLA-DPA1*0109-DPB1*0401 | HLA-DQA1*0108-DQB1*0505 |
| 785 |  | HLA-DPA1*0109-DPB1*0501 | HLA-DQA1*0108-DQB1*0506 |
| 786 |  | HLA-DPA1*0109-DPB1*0601 | HLA-DQA1*0108-DQB1*0507 |
| 787 |  | HLA-DPA1*0109-DPB1*0701 | HLA-DQA1*0108-DQB1*0508 |
| 788 |  | HLA-DPA1*0109-DPB1*0801 | HLA-DQA1*0108-DQB1*0509 |
| 789 |  | HLA-DPA1*0109-DPB1*0901 | HLA-DQA1*0108-DQB1*0510 |
| 790 |  | HLA-DPA1*0109-DPB1*1001 | HLA-DQA1*0108-DQB1*0511 |
| 791 |  | HLA-DPA1*0109-DPB1*1101 | HLA-DQA1*0108-DQB1*0512 |
| 792 |  | HLA-DPA1*0109-DPB1*1101 | HLA-DQA1*0108-DQB1*0513 |
| 793 |  | HLA-DPA1*0109-DPB1*1201 | HLA-DQA1*0108-DQB1*0514 |
| 794 |  | HLA-DPA1*0109-DPB1*1301 | HLA-DQA1*0108-DQB1*0601 |
| 795 |  | HLA-DPA1*0109-DPB1*1401 | HLA-DQA1*0108-DQB1*0602 |
| 796 |  | HLA-DPA1*0109-DPB1*1501 | HLA-DQA1*0108-DQB1*0603 |
| 797 |  | HLA-DPA1*0109-DPB1*1601 | HLA-DQA1*0108-DQB1*0604 |
| 798 |  | HLA-DPA1*0109-DPB1*1701 | HLA-DQA1*0108-DQB1*0607 |
| 799 |  | HLA-DPA1*0109-DPB1*1801 | HLA-DQA1*0108-DQB1*0608 |
| 800 |  | HLA-DPA1*0109-DPB1*1901 | HLA-DQA1*0108-DQB1*0609 |
| 801 |  | HLA-DPA1*0109-DPB1*2101 | HLA-DQA1*0108-DQB1*0610 |
| 802 |  | HLA-DPA1*0109-DPB1*2201 | HLA-DQA1*0108-DQB1*0611 |
| 803 |  | HLA-DPA1*0109-DPB1*2301 | HLA-DQA1*0108-DQB1*0612 |
| 804 |  | HLA-DPA1*0109-DPB1*2401 | HLA-DQA1*0108-DQB1*0614 |
| 805 |  | HLA-DPA1*0109-DPB1*2501 | HLA-DQA1*0108-DQB1*0615 |
| 806 |  | HLA-DPA1*0109-DPB1*2601 | HLA-DQA1*0108-DQB1*0616 |
| 807 |  | HLA-DPA1*0109-DPB1*2701 | HLA-DQA1*0108-DQB1*0617 |
| 808 |  | HLA-DPA1*0109-DPB1*2801 | HLA-DQA1*0108-DQB1*0618 |
| 809 |  | HLA-DPA1*0109-DPB1*2901 | HLA-DQA1*0108-DQB1*0619 |
| 810 |  | HLA-DPA1*0109-DPB1*3001 | HLA-DQA1*0108-DQB1*0621 |
| 811 |  | HLA-DPA1*0109-DPB1*1301 | HLA-DQA1*0108-DQB1*0622 |
| 812 |  | HLA-DPA1*0109-DPB1*3101 | HLA-DQA1*0108-DQB1*0623 |
| 813 |  | HLA-DPA1*0109-DPB1*3201 | HLA-DQA1*0108-DQB1*0624 |
| 814 |  | HLA-DPA1*0109-DPB1*3301 | HLA-DQA1*0108-DQB1*0625 |
| 815 |  | HLA-DPA1*0109-DPB1*3401 | HLA-DQA1*0108-DQB1*0627 |
| 816 |  | HLA-DPA1*0109-DPB1*1401 | HLA-DQA1*0108-DQB1*0628 |
| 817 |  | HLA-DPA1*0109-DPB1*1501 | HLA-DQA1*0108-DQB1*0629 |
| 818 |  | HLA-DPA1*0109-DPB1*1601 | HLA-DQA1*0108-DQB1*0630 |
| 819 |  | HLA-DPA1*0109-DPB1*1701 | HLA-DQA1*0108-DQB1*0631 |
| 820 |  | HLA-DPA1*0109-DPB1*1801 | HLA-DQA1*0108-DQB1*0632 |
| 821 |  | HLA-DPA1*0109-DPB1*1901 | HLA-DQA1*0108-DQB1*0633 |
| 822 |  | HLA-DPA1*0109-DPB1*2001 | HLA-DQA1*0108-DQB1*0634 |
| 823 |  | HLA-DPA1*0109-DPB1*2101 | HLA-DQA1*0108-DQB1*0635 |
| 824 |  | HLA-DPA1*0109-DPB1*2201 | HLA-DQA1*0108-DQB1*0636 |
| 825 |  | HLA-DPA1*0109-DPB1*2301 | HLA-DQA1*0108-DQB1*0637 |
| 826 |  | HLA-DPA1*0109-DPB1*2401 | HLA-DQA1*0108-DQB1*0638 |
| 827 |  | HLA-DPA1*0109-DPB1*2501 | HLA-DQA1*0108-DQB1*0639 |
| 828 |  | HLA-DPA1*0109-DPB1*2601 | HLA-DQA1*0108-DQB1*0640 |
| 829 |  | HLA-DPA1*0109-DPB1*2701 | HLA-DQA1*0108-DQB1*0641 |
| 830 |  | HLA-DPA1*0109-DPB1*2801 | HLA-DQA1*0108-DQB1*0642 |
| 831 |  | HLA-DPA1*0109-DPB1*2901 | HLA-DQA1*0108-DQB1*0643 |
| 832 |  | HLA-DPA1*0109-DPB1*3001 | HLA-DQA1*0108-DQB1*0644 |
| 833 |  | HLA-DPA1*0109-DPB1*3101 | HLA-DQA1*0109-DQB1*0201 |
| 834 |  | HLA-DPA1*0109-DPB1*3201 | HLA-DQA1*0109-DQB1*0202 |
| 835 |  | HLA-DPA1*0109-DPB1*3301 | HLA-DQA1*0109-DQB1*0203 |
| 836 |  | HLA-DPA1*0109-DPB1*3401 | HLA-DQA1*0109-DQB1*0204 |
| 837 |  | HLA-DPA1*0109-DPB1*3501 | HLA-DQA1*0109-DQB1*0205 |
| 838 |  | HLA-DPA1*0109-DPB1*3601 | HLA-DQA1*0109-DQB1*0206 |
| 839 |  | HLA-DPA1*0109-DPB1*3701 | HLA-DQA1*0109-DQB1*0301 |
| 840 |  | HLA-DPA1*0109-DPB1*3801 | HLA-DQA1*0109-DQB1*0302 |
| 841 |  | HLA-DPA1*0109-DPB1*3901 | HLA-DQA1*0109-DQB1*0303 |
| 842 |  | HLA-DPA1*0109-DPB1*4001 | HLA-DQA1*0109-DQB1*0304 |
| 843 |  | HLA-DPA1*0109-DPB1*4101 | HLA-DQA1*0109-DQB1*0305 |
| 844 |  | HLA-DPA1*0109-DPB1*4401 | HLA-DQA1*0109-DQB1*0306 |
| 845 |  | HLA-DPA1*0109-DPB1*4501 | HLA-DQA1*0109-DQB1*0307 |
| 846 |  | HLA-DPA1*0109-DPB1*4601 | HLA-DQA1*0109-DQB1*0308 |
| 847 |  | HLA-DPA1*0109-DPB1*4701 | HLA-DQA1*0109-DQB1*0309 |
| 848 |  | HLA-DPA1*0109-DPB1*4801 | HLA-DQA1*0109-DQB1*0310 |
| 849 |  | HLA-DPA1*0109-DPB1*4901 | HLA-DQA1*0109-DQB1*0311 |
| 850 |  | HLA-DPA1*0109-DPB1*5001 | HLA-DQA1*0109-DQB1*0312 |
| 851 |  | HLA-DPA1*0109-DPB1*5101 | HLA-DQA1*0109-DQB1*0313 |
| 852 |  | HLA-DPA1*0109-DPB1*5201 | HLA-DQA1*0109-DQB1*0314 |
| 853 |  | HLA-DPA1*0109-DPB1*5301 | HLA-DQA1*0109-DQB1*0315 |
| 854 |  | HLA-DPA1*0109-DPB1*5401 | HLA-DQA1*0109-DQB1*0316 |
| 855 |  | HLA-DPA1*0109-DPB1*5501 | HLA-DQA1*0109-DQB1*0317 |
| 856 |  | HLA-DPA1*0109-DPB1*5601 | HLA-DQA1*0109-DQB1*0318 |
| 857 |  | HLA-DPA1*0109-DPB1*5801 | HLA-DQA1*0109-DQB1*0319 |
| 858 |  | HLA-DPA1*0109-DPB1*5901 | HLA-DQA1*0109-DQB1*0320 |
| 859 |  | HLA-DPA1*0109-DPB1*6001 | HLA-DQA1*0109-DQB1*0321 |
| 860 |  | HLA-DPA1*0109-DPB1*6201 | HLA-DQA1*0109-DQB1*0322 |
| 861 |  | HLA-DPA1*0109-DPB1*6301 | HLA-DQA1*0109-DQB1*0323 |
| 862 |  | HLA-DPA1*0109-DPB1*6501 | HLA-DQA1*0109-DQB1*0324 |
| 863 |  | HLA-DPA1*0109-DPB1*6601 | HLA-DQA1*0109-DQB1*0325 |
| 864 |  | HLA-DPA1*0109-DPB1*6701 | HLA-DQA1*0109-DQB1*0326 |
| 865 |  | HLA-DPA1*0109-DPB1*6801 | HLA-DQA1*0109-DQB1*0327 |
| 866 |  | HLA-DPA1*0109-DPB1*6901 | HLA-DQA1*0109-DQB1*0328 |
| 867 |  | HLA-DPA1*0109-DPB1*7001 | HLA-DQA1*0109-DQB1*0329 |
| 868 |  | HLA-DPA1*0109-DPB1*7101 | HLA-DQA1*0109-DQB1*0330 |
| 869 |  | HLA-DPA1*0109-DPB1*7201 | HLA-DQA1*0109-DQB1*0331 |
| 870 |  | HLA-DPA1*0109-DPB1*7301 | HLA-DQA1*0109-DQB1*0332 |
| 871 |  | HLA-DPA1*0109-DPB1*7401 | HLA-DQA1*0109-DQB1*0333 |
| 872 |  | HLA-DPA1*0109-DPB1*7501 | HLA-DQA1*0109-DQB1*0334 |
| 873 |  | HLA-DPA1*0109-DPB1*7601 | HLA-DQA1*0109-DQB1*0335 |
| 874 |  | HLA-DPA1*0109-DPB1*7701 | HLA-DQA1*0109-DQB1*0336 |
| 875 |  | HLA-DPA1*0109-DPB1*7801 | HLA-DQA1*0109-DQB1*0337 |
| 876 |  | HLA-DPA1*0109-DPB1*7901 | HLA-DQA1*0109-DQB1*0338 |
| 877 |  | HLA-DPA1*0109-DPB1*8001 | HLA-DQA1*0109-DQB1*0401 |
| 878 |  | HLA-DPA1*0109-DPB1*8101 | HLA-DQA1*0109-DQB1*0402 |
| 879 |  | HLA-DPA1*0109-DPB1*8201 | HLA-DQA1*0109-DQB1*0403 |
| 880 |  | HLA-DPA1*0109-DPB1*8301 | HLA-DQA1*0109-DQB1*0404 |
| 881 |  | HLA-DPA1*0109-DPB1*8401 | HLA-DQA1*0109-DQB1*0405 |
| 882 |  | HLA-DPA1*0109-DPB1*8501 | HLA-DQA1*0109-DQB1*0406 |
| 883 |  | HLA-DPA1*0109-DPB1*8601 | HLA-DQA1*0109-DQB1*0407 |
| 884 |  | HLA-DPA1*0109-DPB1*8701 | HLA-DQA1*0109-DQB1*0408 |
| 885 |  | HLA-DPA1*0109-DPB1*8801 | HLA-DQA1*0109-DQB1*0501 |
| 886 |  | HLA-DPA1*0109-DPB1*8901 | HLA-DQA1*0109-DQB1*0502 |
| 887 |  | HLA-DPA1*0109-DPB1*9001 | HLA-DQA1*0109-DQB1*0503 |
| 888 |  | HLA-DPA1*0109-DPB1*9101 | HLA-DQA1*0109-DQB1*0505 |
| 889 |  | HLA-DPA1*0109-DPB1*9201 | HLA-DQA1*0109-DQB1*0506 |
| 890 |  | HLA-DPA1*0109-DPB1*9301 | HLA-DQA1*0109-DQB1*0507 |
| 891 |  | HLA-DPA1*0109-DPB1*9401 | HLA-DQA1*0109-DQB1*0508 |
| 892 |  | HLA-DPA1*0109-DPB1*9501 | HLA-DQA1*0109-DQB1*0509 |
| 893 |  | HLA-DPA1*0109-DPB1*9601 | HLA-DQA1*0109-DQB1*0510 |
| 894 |  | HLA-DPA1*0109-DPB1*9701 | HLA-DQA1*0109-DQB1*0511 |
| 895 |  | HLA-DPA1*0109-DPB1*9801 | HLA-DQA1*0109-DQB1*0512 |
| 896 |  | HLA-DPA1*0109-DPB1*9901 | HLA-DQA1*0109-DQB1*0513 |
| 897 |  | HLA-DPA1*0110-DPB1*0101 | HLA-DQA1*0109-DQB1*0514 |
| 898 |  | HLA-DPA1*0110-DPB1*0201 | HLA-DQA1*0109-DQB1*0601 |
| 899 |  | HLA-DPA1*0110-DPB1*0202 | HLA-DQA1*0109-DQB1*0602 |
| 900 |  | HLA-DPA1*0110-DPB1*0301 | HLA-DQA1*0109-DQB1*0603 |
| 901 |  | HLA-DPA1*0110-DPB1*0401 | HLA-DQA1*0109-DQB1*0604 |
| 902 |  | HLA-DPA1*0110-DPB1*0402 | HLA-DQA1*0109-DQB1*0607 |
| 903 |  | HLA-DPA1*0110-DPB1*0501 | HLA-DQA1*0109-DQB1*0608 |
| 904 |  | HLA-DPA1*0110-DPB1*0601 | HLA-DQA1*0109-DQB1*0609 |
| 905 |  | HLA-DPA1*0110-DPB1*0801 | HLA-DQA1*0109-DQB1*0610 |
| 906 |  | HLA-DPA1*0110-DPB1*0901 | HLA-DQA1*0109-DQB1*0611 |
| 907 |  | HLA-DPA1*0110-DPB1*0001 | HLA-DQA1*0109-DQB1*0612 |
| 908 |  | HLA-DPA1*0110-DPB1*1001 | HLA-DQA1*0109-DQB1*0614 |
| 909 |  | HLA-DPA1*0110-DPB1*0101 | HLA-DQA1*0109-DQB1*0615 |
| 910 |  | HLA-DPA1*0110-DPB1*0201 | HLA-DQA1*0109-DQB1*0616 |
| 911 |  | HLA-DPA1*0110-DPB1*0301 | HLA-DQA1*0109-DQB1*0617 |
| 912 |  | HLA-DPA1*0110-DPB1*0401 | HLA-DQA1*0109-DQB1*0618 |
| 913 |  | HLA-DPA1*0110-DPB1*0501 | HLA-DQA1*0109-DQB1*0619 |
| 914 |  | HLA-DPA1*0110-DPB1*0601 | HLA-DQA1*0109-DQB1*0621 |
| 915 |  | HLA-DPA1*0110-DPB1*0701 | HLA-DQA1*0109-DQB1*0622 |
| 916 |  | HLA-DPA1*0110-DPB1*0801 | HLA-DQA1*0109-DQB1*0623 |
| 917 |  | HLA-DPA1*0110-DPB1*0901 | HLA-DQA1*0109-DQB1*0624 |
| 918 |  | HLA-DPA1*0110-DPB1*1001 | HLA-DQA1*0109-DQB1*0625 |
| 919 |  | HLA-DPA1*0110-DPB1*1101 | HLA-DQA1*0109-DQB1*0627 |
| 920 |  | HLA-DPA1*0110-DPB1*1101 | HLA-DQA1*0109-DQB1*0628 |
| 921 |  | HLA-DPA1*0110-DPB1*1201 | HLA-DQA1*0109-DQB1*0629 |
| 922 |  | HLA-DPA1*0110-DPB1*1301 | HLA-DQA1*0109-DQB1*0630 |
| 923 |  | HLA-DPA1*0110-DPB1*1401 | HLA-DQA1*0109-DQB1*0631 |
| 924 |  | HLA-DPA1*0110-DPB1*1501 | HLA-DQA1*0109-DQB1*0632 |
| 925 |  | HLA-DPA1*0110-DPB1*1601 | HLA-DQA1*0109-DQB1*0633 |
| 926 |  | HLA-DPA1*0110-DPB1*1701 | HLA-DQA1*0109-DQB1*0634 |
| 927 |  | HLA-DPA1*0110-DPB1*1801 | HLA-DQA1*0109-DQB1*0635 |
| 928 |  | HLA-DPA1*0110-DPB1*1901 | HLA-DQA1*0109-DQB1*0636 |
| 929 |  | HLA-DPA1*0110-DPB1*2101 | HLA-DQA1*0109-DQB1*0637 |
| 930 |  | HLA-DPA1*0110-DPB1*2201 | HLA-DQA1*0109-DQB1*0638 |
| 931 |  | HLA-DPA1*0110-DPB1*2301 | HLA-DQA1*0109-DQB1*0639 |
| 932 |  | HLA-DPA1*0110-DPB1*2401 | HLA-DQA1*0109-DQB1*0640 |
| 933 |  | HLA-DPA1*0110-DPB1*2501 | HLA-DQA1*0109-DQB1*0641 |
| 934 |  | HLA-DPA1*0110-DPB1*2601 | HLA-DQA1*0109-DQB1*0642 |
| 935 |  | HLA-DPA1*0110-DPB1*2701 | HLA-DQA1*0109-DQB1*0643 |
| 936 |  | HLA-DPA1*0110-DPB1*2801 | HLA-DQA1*0109-DQB1*0644 |
| 937 |  | HLA-DPA1*0110-DPB1*2901 | HLA-DQA1*0201-DQB1*0201 |
| 938 |  | HLA-DPA1*0110-DPB1*3001 | HLA-DQA1*0201-DQB1*0202 |
| 939 |  | HLA-DPA1*0110-DPB1*1301 | HLA-DQA1*0201-DQB1*0203 |
| 940 |  | HLA-DPA1*0110-DPB1*3101 | HLA-DQA1*0201-DQB1*0204 |
| 941 |  | HLA-DPA1*0110-DPB1*3201 | HLA-DQA1*0201-DQB1*0205 |
| 942 |  | HLA-DPA1*0110-DPB1*3301 | HLA-DQA1*0201-DQB1*0206 |
| 943 |  | HLA-DPA1*0110-DPB1*3401 | HLA-DQA1*0201-DQB1*0301 |
| 944 |  | HLA-DPA1*0110-DPB1*1401 | HLA-DQA1*0201-DQB1*0302 |
| 945 |  | HLA-DPA1*0110-DPB1*1501 | HLA-DQA1*0201-DQB1*0303 |
| 946 |  | HLA-DPA1*0110-DPB1*1601 | HLA-DQA1*0201-DQB1*0304 |
| 947 |  | HLA-DPA1*0110-DPB1*1701 | HLA-DQA1*0201-DQB1*0305 |
| 948 |  | HLA-DPA1*0110-DPB1*1801 | HLA-DQA1*0201-DQB1*0306 |
| 949 |  | HLA-DPA1*0110-DPB1*1901 | HLA-DQA1*0201-DQB1*0307 |
| 950 |  | HLA-DPA1*0110-DPB1*2001 | HLA-DQA1*0201-DQB1*0308 |
| 951 |  | HLA-DPA1*0110-DPB1*2101 | HLA-DQA1*0201-DQB1*0309 |
| 952 |  | HLA-DPA1*0110-DPB1*2201 | HLA-DQA1*0201-DQB1*0310 |
| 953 |  | HLA-DPA1*0110-DPB1*2301 | HLA-DQA1*0201-DQB1*0311 |
| 954 |  | HLA-DPA1*0110-DPB1*2401 | HLA-DQA1*0201-DQB1*0312 |
| 955 |  | HLA-DPA1*0110-DPB1*2501 | HLA-DQA1*0201-DQB1*0313 |
| 956 |  | HLA-DPA1*0110-DPB1*2601 | HLA-DQA1*0201-DQB1*0314 |
| 957 |  | HLA-DPA1*0110-DPB1*2701 | HLA-DQA1*0201-DQB1*0315 |
| 958 |  | HLA-DPA1*0110-DPB1*2801 | HLA-DQA1*0201-DQB1*0316 |
| 959 |  | HLA-DPA1*0110-DPB1*2901 | HLA-DQA1*0201-DQB1*0317 |
| 960 |  | HLA-DPA1*0110-DPB1*3001 | HLA-DQA1*0201-DQB1*0318 |
| 961 |  | HLA-DPA1*0110-DPB1*3101 | HLA-DQA1*0201-DQB1*0319 |
| 962 |  | HLA-DPA1*0110-DPB1*3201 | HLA-DQA1*0201-DQB1*0320 |
| 963 |  | HLA-DPA1*0110-DPB1*3301 | HLA-DQA1*0201-DQB1*0321 |
| 964 |  | HLA-DPA1*0110-DPB1*3401 | HLA-DQA1*0201-DQB1*0322 |
| 965 |  | HLA-DPA1*0110-DPB1*3501 | HLA-DQA1*0201-DQB1*0323 |
| 966 |  | HLA-DPA1*0110-DPB1*3601 | HLA-DQA1*0201-DQB1*0324 |
| 967 |  | HLA-DPA1*0110-DPB1*3701 | HLA-DQA1*0201-DQB1*0325 |
| 968 |  | HLA-DPA1*0110-DPB1*3801 | HLA-DQA1*0201-DQB1*0326 |
| 969 |  | HLA-DPA1*0110-DPB1*3901 | HLA-DQA1*0201-DQB1*0327 |
| 970 |  | HLA-DPA1*0110-DPB1*4001 | HLA-DQA1*0201-DQB1*0328 |
| 971 |  | HLA-DPA1*0110-DPB1*4101 | HLA-DQA1*0201-DQB1*0329 |
| 972 |  | HLA-DPA1*0110-DPB1*4401 | HLA-DQA1*0201-DQB1*0330 |
| 973 |  | HLA-DPA1*0110-DPB1*4501 | HLA-DQA1*0201-DQB1*0331 |
| 974 |  | HLA-DPA1*0110-DPB1*4601 | HLA-DQA1*0201-DQB1*0332 |
| 975 |  | HLA-DPA1*0110-DPB1*4701 | HLA-DQA1*0201-DQB1*0333 |
| 976 |  | HLA-DPA1*0110-DPB1*4801 | HLA-DQA1*0201-DQB1*0334 |
| 977 |  | HLA-DPA1*0110-DPB1*4901 | HLA-DQA1*0201-DQB1*0335 |
| 978 |  | HLA-DPA1*0110-DPB1*5001 | HLA-DQA1*0201-DQB1*0336 |
| 979 |  | HLA-DPA1*0110-DPB1*5101 | HLA-DQA1*0201-DQB1*0337 |
| 980 |  | HLA-DPA1*0110-DPB1*5201 | HLA-DQA1*0201-DQB1*0338 |
| 981 |  | HLA-DPA1*0110-DPB1*5301 | HLA-DQA1*0201-DQB1*0401 |
| 982 |  | HLA-DPA1*0110-DPB1*5401 | HLA-DQA1*0201-DQB1*0402 |
| 983 |  | HLA-DPA1*0110-DPB1*5501 | HLA-DQA1*0201-DQB1*0403 |
| 984 |  | HLA-DPA1*0110-DPB1*5601 | HLA-DQA1*0201-DQB1*0404 |
| 985 |  | HLA-DPA1*0110-DPB1*5801 | HLA-DQA1*0201-DQB1*0405 |
| 986 |  | HLA-DPA1*0110-DPB1*5901 | HLA-DQA1*0201-DQB1*0406 |
| 987 |  | HLA-DPA1*0110-DPB1*6001 | HLA-DQA1*0201-DQB1*0407 |
| 988 |  | HLA-DPA1*0110-DPB1*6201 | HLA-DQA1*0201-DQB1*0408 |
| 989 |  | HLA-DPA1*0110-DPB1*6301 | HLA-DQA1*0201-DQB1*0501 |
| 990 |  | HLA-DPA1*0110-DPB1*6501 | HLA-DQA1*0201-DQB1*0502 |
| 991 |  | HLA-DPA1*0110-DPB1*6601 | HLA-DQA1*0201-DQB1*0503 |
| 992 |  | HLA-DPA1*0110-DPB1*6701 | HLA-DQA1*0201-DQB1*0505 |
| 993 |  | HLA-DPA1*0110-DPB1*6801 | HLA-DQA1*0201-DQB1*0506 |
| 994 |  | HLA-DPA1*0110-DPB1*6901 | HLA-DQA1*0201-DQB1*0507 |
| 995 |  | HLA-DPA1*0110-DPB1*7001 | HLA-DQA1*0201-DQB1*0508 |
| 996 |  | HLA-DPA1*0110-DPB1*7101 | HLA-DQA1*0201-DQB1*0509 |
| 997 |  | HLA-DPA1*0110-DPB1*7201 | HLA-DQA1*0201-DQB1*0510 |
| 998 |  | HLA-DPA1*0110-DPB1*7301 | HLA-DQA1*0201-DQB1*0511 |
| 999 |  | HLA-DPA1*0110-DPB1*7401 | HLA-DQA1*0201-DQB1*0512 |
| 1000 |  | HLA-DPA1*0110-DPB1*7501 | HLA-DQA1*0201-DQB1*0513 |
| 1001 |  | HLA-DPA1*0110-DPB1*7601 | HLA-DQA1*0201-DQB1*0514 |
| 1002 |  | HLA-DPA1*0110-DPB1*7701 | HLA-DQA1*0201-DQB1*0601 |
| 1003 |  | HLA-DPA1*0110-DPB1*7801 | HLA-DQA1*0201-DQB1*0602 |
| 1004 |  | HLA-DPA1*0110-DPB1*7901 | HLA-DQA1*0201-DQB1*0603 |
| 1005 |  | HLA-DPA1*0110-DPB1*8001 | HLA-DQA1*0201-DQB1*0604 |
| 1006 |  | HLA-DPA1*0110-DPB1*8101 | HLA-DQA1*0201-DQB1*0607 |
| 1007 |  | HLA-DPA1*0110-DPB1*8201 | HLA-DQA1*0201-DQB1*0608 |
| 1008 |  | HLA-DPA1*0110-DPB1*8301 | HLA-DQA1*0201-DQB1*0609 |
| 1009 |  | HLA-DPA1*0110-DPB1*8401 | HLA-DQA1*0201-DQB1*0610 |
| 1010 |  | HLA-DPA1*0110-DPB1*8501 | HLA-DQA1*0201-DQB1*0611 |
| 1011 |  | HLA-DPA1*0110-DPB1*8601 | HLA-DQA1*0201-DQB1*0612 |
| 1012 |  | HLA-DPA1*0110-DPB1*8701 | HLA-DQA1*0201-DQB1*0614 |
| 1013 |  | HLA-DPA1*0110-DPB1*8801 | HLA-DQA1*0201-DQB1*0615 |
| 1014 |  | HLA-DPA1*0110-DPB1*8901 | HLA-DQA1*0201-DQB1*0616 |
| 1015 |  | HLA-DPA1*0110-DPB1*9001 | HLA-DQA1*0201-DQB1*0617 |
| 1016 |  | HLA-DPA1*0110-DPB1*9101 | HLA-DQA1*0201-DQB1*0618 |
| 1017 |  | HLA-DPA1*0110-DPB1*9201 | HLA-DQA1*0201-DQB1*0619 |
| 1018 |  | HLA-DPA1*0110-DPB1*9301 | HLA-DQA1*0201-DQB1*0621 |
| 1019 |  | HLA-DPA1*0110-DPB1*9401 | HLA-DQA1*0201-DQB1*0622 |
| 1020 |  | HLA-DPA1*0110-DPB1*9501 | HLA-DQA1*0201-DQB1*0623 |
| 1021 |  | HLA-DPA1*0110-DPB1*9601 | HLA-DQA1*0201-DQB1*0624 |
| 1022 |  | HLA-DPA1*0110-DPB1*9701 | HLA-DQA1*0201-DQB1*0625 |
| 1023 |  | HLA-DPA1*0110-DPB1*9801 | HLA-DQA1*0201-DQB1*0627 |
| 1024 |  | HLA-DPA1*0110-DPB1*9901 | HLA-DQA1*0201-DQB1*0628 |
| 1025 |  | HLA-DPA1*0201-DPB1*0101 | HLA-DQA1*0201-DQB1*0629 |
| 1026 |  | HLA-DPA1*0201-DPB1*0201 | HLA-DQA1*0201-DQB1*0630 |
| 1027 |  | HLA-DPA1*0201-DPB1*0202 | HLA-DQA1*0201-DQB1*0631 |
| 1028 |  | HLA-DPA1*0201-DPB1*0301 | HLA-DQA1*0201-DQB1*0632 |
| 1029 |  | HLA-DPA1*0201-DPB1*0401 | HLA-DQA1*0201-DQB1*0633 |
| 1030 |  | HLA-DPA1*0201-DPB1*0402 | HLA-DQA1*0201-DQB1*0634 |
| 1031 |  | HLA-DPA1*0201-DPB1*0501 | HLA-DQA1*0201-DQB1*0635 |
| 1032 |  | HLA-DPA1*0201-DPB1*0601 | HLA-DQA1*0201-DQB1*0636 |
| 1033 |  | HLA-DPA1*0201-DPB1*0801 | HLA-DQA1*0201-DQB1*0637 |
| 1034 |  | HLA-DPA1*0201-DPB1*0901 | HLA-DQA1*0201-DQB1*0638 |
| 1035 |  | HLA-DPA1*0201-DPB1*0001 | HLA-DQA1*0201-DQB1*0639 |
| 1036 |  | HLA-DPA1*0201-DPB1*1001 | HLA-DQA1*0201-DQB1*0640 |
| 1037 |  | HLA-DPA1*0201-DPB1*0101 | HLA-DQA1*0201-DQB1*0641 |
| 1038 |  | HLA-DPA1*0201-DPB1*0201 | HLA-DQA1*0201-DQB1*0642 |
| 1039 |  | HLA-DPA1*0201-DPB1*0301 | HLA-DQA1*0201-DQB1*0643 |
| 1040 |  | HLA-DPA1*0201-DPB1*0401 | HLA-DQA1*0201-DQB1*0644 |
| 1041 |  | HLA-DPA1*0201-DPB1*0501 | HLA-DQA1*0301-DQB1*0201 |
| 1042 |  | HLA-DPA1*0201-DPB1*0601 | HLA-DQA1*0301-DQB1*0202 |
| 1043 |  | HLA-DPA1*0201-DPB1*0701 | HLA-DQA1*0301-DQB1*0203 |
| 1044 |  | HLA-DPA1*0201-DPB1*0801 | HLA-DQA1*0301-DQB1*0204 |
| 1045 |  | HLA-DPA1*0201-DPB1*0901 | HLA-DQA1*0301-DQB1*0205 |
| 1046 |  | HLA-DPA1*0201-DPB1*1001 | HLA-DQA1*0301-DQB1*0206 |
| 1047 |  | HLA-DPA1*0201-DPB1*1101 | HLA-DQA1*0301-DQB1*0301 |
| 1048 |  | HLA-DPA1*0201-DPB1*1101 | HLA-DQA1*0301-DQB1*0302 |
| 1049 |  | HLA-DPA1*0201-DPB1*1201 | HLA-DQA1*0301-DQB1*0303 |
| 1050 |  | HLA-DPA1*0201-DPB1*1301 | HLA-DQA1*0301-DQB1*0304 |
| 1051 |  | HLA-DPA1*0201-DPB1*1401 | HLA-DQA1*0301-DQB1*0305 |
| 1052 |  | HLA-DPA1*0201-DPB1*1501 | HLA-DQA1*0301-DQB1*0306 |
| 1053 |  | HLA-DPA1*0201-DPB1*1601 | HLA-DQA1*0301-DQB1*0307 |
| 1054 |  | HLA-DPA1*0201-DPB1*1701 | HLA-DQA1*0301-DQB1*0308 |
| 1055 |  | HLA-DPA1*0201-DPB1*1801 | HLA-DQA1*0301-DQB1*0309 |
| 1056 |  | HLA-DPA1*0201-DPB1*1901 | HLA-DQA1*0301-DQB1*0310 |
| 1057 |  | HLA-DPA1*0201-DPB1*2101 | HLA-DQA1*0301-DQB1*0311 |
| 1058 |  | HLA-DPA1*0201-DPB1*2201 | HLA-DQA1*0301-DQB1*0312 |
| 1059 |  | HLA-DPA1*0201-DPB1*2301 | HLA-DQA1*0301-DQB1*0313 |
| 1060 |  | HLA-DPA1*0201-DPB1*2401 | HLA-DQA1*0301-DQB1*0314 |
| 1061 |  | HLA-DPA1*0201-DPB1*2501 | HLA-DQA1*0301-DQB1*0315 |
| 1062 |  | HLA-DPA1*0201-DPB1*2601 | HLA-DQA1*0301-DQB1*0316 |
| 1063 |  | HLA-DPA1*0201-DPB1*2701 | HLA-DQA1*0301-DQB1*0317 |
| 1064 |  | HLA-DPA1*0201-DPB1*2801 | HLA-DQA1*0301-DQB1*0318 |
| 1065 |  | HLA-DPA1*0201-DPB1*2901 | HLA-DQA1*0301-DQB1*0319 |
| 1066 |  | HLA-DPA1*0201-DPB1*3001 | HLA-DQA1*0301-DQB1*0320 |
| 1067 |  | HLA-DPA1*0201-DPB1*1301 | HLA-DQA1*0301-DQB1*0321 |
| 1068 |  | HLA-DPA1*0201-DPB1*3101 | HLA-DQA1*0301-DQB1*0322 |
| 1069 |  | HLA-DPA1*0201-DPB1*3201 | HLA-DQA1*0301-DQB1*0323 |
| 1070 |  | HLA-DPA1*0201-DPB1*3301 | HLA-DQA1*0301-DQB1*0324 |
| 1071 |  | HLA-DPA1*0201-DPB1*3401 | HLA-DQA1*0301-DQB1*0325 |
| 1072 |  | HLA-DPA1*0201-DPB1*1401 | HLA-DQA1*0301-DQB1*0326 |
| 1073 |  | HLA-DPA1*0201-DPB1*1501 | HLA-DQA1*0301-DQB1*0327 |
| 1074 |  | HLA-DPA1*0201-DPB1*1601 | HLA-DQA1*0301-DQB1*0328 |
| 1075 |  | HLA-DPA1*0201-DPB1*1701 | HLA-DQA1*0301-DQB1*0329 |
| 1076 |  | HLA-DPA1*0201-DPB1*1801 | HLA-DQA1*0301-DQB1*0330 |
| 1077 |  | HLA-DPA1*0201-DPB1*1901 | HLA-DQA1*0301-DQB1*0331 |
| 1078 |  | HLA-DPA1*0201-DPB1*2001 | HLA-DQA1*0301-DQB1*0332 |
| 1079 |  | HLA-DPA1*0201-DPB1*2101 | HLA-DQA1*0301-DQB1*0333 |
| 1080 |  | HLA-DPA1*0201-DPB1*2201 | HLA-DQA1*0301-DQB1*0334 |
| 1081 |  | HLA-DPA1*0201-DPB1*2301 | HLA-DQA1*0301-DQB1*0335 |
| 1082 |  | HLA-DPA1*0201-DPB1*2401 | HLA-DQA1*0301-DQB1*0336 |
| 1083 |  | HLA-DPA1*0201-DPB1*2501 | HLA-DQA1*0301-DQB1*0337 |
| 1084 |  | HLA-DPA1*0201-DPB1*2601 | HLA-DQA1*0301-DQB1*0338 |
| 1085 |  | HLA-DPA1*0201-DPB1*2701 | HLA-DQA1*0301-DQB1*0401 |
| 1086 |  | HLA-DPA1*0201-DPB1*2801 | HLA-DQA1*0301-DQB1*0402 |
| 1087 |  | HLA-DPA1*0201-DPB1*2901 | HLA-DQA1*0301-DQB1*0403 |
| 1088 |  | HLA-DPA1*0201-DPB1*3001 | HLA-DQA1*0301-DQB1*0404 |
| 1089 |  | HLA-DPA1*0201-DPB1*3101 | HLA-DQA1*0301-DQB1*0405 |
| 1090 |  | HLA-DPA1*0201-DPB1*3201 | HLA-DQA1*0301-DQB1*0406 |
| 1091 |  | HLA-DPA1*0201-DPB1*3301 | HLA-DQA1*0301-DQB1*0407 |
| 1092 |  | HLA-DPA1*0201-DPB1*3401 | HLA-DQA1*0301-DQB1*0408 |
| 1093 |  | HLA-DPA1*0201-DPB1*3501 | HLA-DQA1*0301-DQB1*0501 |
| 1094 |  | HLA-DPA1*0201-DPB1*3601 | HLA-DQA1*0301-DQB1*0502 |
| 1095 |  | HLA-DPA1*0201-DPB1*3701 | HLA-DQA1*0301-DQB1*0503 |
| 1096 |  | HLA-DPA1*0201-DPB1*3801 | HLA-DQA1*0301-DQB1*0505 |
| 1097 |  | HLA-DPA1*0201-DPB1*3901 | HLA-DQA1*0301-DQB1*0506 |
| 1098 |  | HLA-DPA1*0201-DPB1*4001 | HLA-DQA1*0301-DQB1*0507 |
| 1099 |  | HLA-DPA1*0201-DPB1*4101 | HLA-DQA1*0301-DQB1*0508 |
| 1100 |  | HLA-DPA1*0201-DPB1*4401 | HLA-DQA1*0301-DQB1*0509 |
| 1101 |  | HLA-DPA1*0201-DPB1*4501 | HLA-DQA1*0301-DQB1*0510 |
| 1102 |  | HLA-DPA1*0201-DPB1*4601 | HLA-DQA1*0301-DQB1*0511 |
| 1103 |  | HLA-DPA1*0201-DPB1*4701 | HLA-DQA1*0301-DQB1*0512 |
| 1104 |  | HLA-DPA1*0201-DPB1*4801 | HLA-DQA1*0301-DQB1*0513 |
| 1105 |  | HLA-DPA1*0201-DPB1*4901 | HLA-DQA1*0301-DQB1*0514 |
| 1106 |  | HLA-DPA1*0201-DPB1*5001 | HLA-DQA1*0301-DQB1*0601 |
| 1107 |  | HLA-DPA1*0201-DPB1*5101 | HLA-DQA1*0301-DQB1*0602 |
| 1108 |  | HLA-DPA1*0201-DPB1*5201 | HLA-DQA1*0301-DQB1*0603 |
| 1109 |  | HLA-DPA1*0201-DPB1*5301 | HLA-DQA1*0301-DQB1*0604 |
| 1110 |  | HLA-DPA1*0201-DPB1*5401 | HLA-DQA1*0301-DQB1*0607 |
| 1111 |  | HLA-DPA1*0201-DPB1*5501 | HLA-DQA1*0301-DQB1*0608 |
| 1112 |  | HLA-DPA1*0201-DPB1*5601 | HLA-DQA1*0301-DQB1*0609 |
| 1113 |  | HLA-DPA1*0201-DPB1*5801 | HLA-DQA1*0301-DQB1*0610 |
| 1114 |  | HLA-DPA1*0201-DPB1*5901 | HLA-DQA1*0301-DQB1*0611 |
| 1115 |  | HLA-DPA1*0201-DPB1*6001 | HLA-DQA1*0301-DQB1*0612 |
| 1116 |  | HLA-DPA1*0201-DPB1*6201 | HLA-DQA1*0301-DQB1*0614 |
| 1117 |  | HLA-DPA1*0201-DPB1*6301 | HLA-DQA1*0301-DQB1*0615 |
| 1118 |  | HLA-DPA1*0201-DPB1*6501 | HLA-DQA1*0301-DQB1*0616 |
| 1119 |  | HLA-DPA1*0201-DPB1*6601 | HLA-DQA1*0301-DQB1*0617 |
| 1120 |  | HLA-DPA1*0201-DPB1*6701 | HLA-DQA1*0301-DQB1*0618 |
| 1121 |  | HLA-DPA1*0201-DPB1*6801 | HLA-DQA1*0301-DQB1*0619 |
| 1122 |  | HLA-DPA1*0201-DPB1*6901 | HLA-DQA1*0301-DQB1*0621 |
| 1123 |  | HLA-DPA1*0201-DPB1*7001 | HLA-DQA1*0301-DQB1*0622 |
| 1124 |  | HLA-DPA1*0201-DPB1*7101 | HLA-DQA1*0301-DQB1*0623 |
| 1125 |  | HLA-DPA1*0201-DPB1*7201 | HLA-DQA1*0301-DQB1*0624 |
| 1126 |  | HLA-DPA1*0201-DPB1*7301 | HLA-DQA1*0301-DQB1*0625 |
| 1127 |  | HLA-DPA1*0201-DPB1*7401 | HLA-DQA1*0301-DQB1*0627 |
| 1128 |  | HLA-DPA1*0201-DPB1*7501 | HLA-DQA1*0301-DQB1*0628 |
| 1129 |  | HLA-DPA1*0201-DPB1*7601 | HLA-DQA1*0301-DQB1*0629 |
| 1130 |  | HLA-DPA1*0201-DPB1*7701 | HLA-DQA1*0301-DQB1*0630 |
| 1131 |  | HLA-DPA1*0201-DPB1*7801 | HLA-DQA1*0301-DQB1*0631 |
| 1132 |  | HLA-DPA1*0201-DPB1*7901 | HLA-DQA1*0301-DQB1*0632 |
| 1133 |  | HLA-DPA1*0201-DPB1*8001 | HLA-DQA1*0301-DQB1*0633 |
| 1134 |  | HLA-DPA1*0201-DPB1*8101 | HLA-DQA1*0301-DQB1*0634 |
| 1135 |  | HLA-DPA1*0201-DPB1*8201 | HLA-DQA1*0301-DQB1*0635 |
| 1136 |  | HLA-DPA1*0201-DPB1*8301 | HLA-DQA1*0301-DQB1*0636 |
| 1137 |  | HLA-DPA1*0201-DPB1*8401 | HLA-DQA1*0301-DQB1*0637 |
| 1138 |  | HLA-DPA1*0201-DPB1*8501 | HLA-DQA1*0301-DQB1*0638 |
| 1139 |  | HLA-DPA1*0201-DPB1*8601 | HLA-DQA1*0301-DQB1*0639 |
| 1140 |  | HLA-DPA1*0201-DPB1*8701 | HLA-DQA1*0301-DQB1*0640 |
| 1141 |  | HLA-DPA1*0201-DPB1*8801 | HLA-DQA1*0301-DQB1*0641 |
| 1142 |  | HLA-DPA1*0201-DPB1*8901 | HLA-DQA1*0301-DQB1*0642 |
| 1143 |  | HLA-DPA1*0201-DPB1*9001 | HLA-DQA1*0301-DQB1*0643 |
| 1144 |  | HLA-DPA1*0201-DPB1*9101 | HLA-DQA1*0301-DQB1*0644 |
| 1145 |  | HLA-DPA1*0201-DPB1*9201 | HLA-DQA1*0302-DQB1*0201 |
| 1146 |  | HLA-DPA1*0201-DPB1*9301 | HLA-DQA1*0302-DQB1*0202 |
| 1147 |  | HLA-DPA1*0201-DPB1*9401 | HLA-DQA1*0302-DQB1*0203 |
| 1148 |  | HLA-DPA1*0201-DPB1*9501 | HLA-DQA1*0302-DQB1*0204 |
| 1149 |  | HLA-DPA1*0201-DPB1*9601 | HLA-DQA1*0302-DQB1*0205 |
| 1150 |  | HLA-DPA1*0201-DPB1*9701 | HLA-DQA1*0302-DQB1*0206 |
| 1151 |  | HLA-DPA1*0201-DPB1*9801 | HLA-DQA1*0302-DQB1*0301 |
| 1152 |  | HLA-DPA1*0201-DPB1*9901 | HLA-DQA1*0302-DQB1*0302 |
| 1153 |  | HLA-DPA1*0202-DPB1*0101 | HLA-DQA1*0302-DQB1*0303 |
| 1154 |  | HLA-DPA1*0202-DPB1*0201 | HLA-DQA1*0302-DQB1*0304 |
| 1155 |  | HLA-DPA1*0202-DPB1*0202 | HLA-DQA1*0302-DQB1*0305 |
| 1156 |  | HLA-DPA1*0202-DPB1*0301 | HLA-DQA1*0302-DQB1*0306 |
| 1157 |  | HLA-DPA1*0202-DPB1*0401 | HLA-DQA1*0302-DQB1*0307 |
| 1158 |  | HLA-DPA1*0202-DPB1*0402 | HLA-DQA1*0302-DQB1*0308 |
| 1159 |  | HLA-DPA1*0202-DPB1*0501 | HLA-DQA1*0302-DQB1*0309 |
| 1160 |  | HLA-DPA1*0202-DPB1*0601 | HLA-DQA1*0302-DQB1*0310 |
| 1161 |  | HLA-DPA1*0202-DPB1*0801 | HLA-DQA1*0302-DQB1*0311 |
| 1162 |  | HLA-DPA1*0202-DPB1*0901 | HLA-DQA1*0302-DQB1*0312 |
| 1163 |  | HLA-DPA1*0202-DPB1*0001 | HLA-DQA1*0302-DQB1*0313 |
| 1164 |  | HLA-DPA1*0202-DPB1*1001 | HLA-DQA1*0302-DQB1*0314 |
| 1165 |  | HLA-DPA1*0202-DPB1*0101 | HLA-DQA1*0302-DQB1*0315 |
| 1166 |  | HLA-DPA1*0202-DPB1*0201 | HLA-DQA1*0302-DQB1*0316 |
| 1167 |  | HLA-DPA1*0202-DPB1*0301 | HLA-DQA1*0302-DQB1*0317 |
| 1168 |  | HLA-DPA1*0202-DPB1*0401 | HLA-DQA1*0302-DQB1*0318 |
| 1169 |  | HLA-DPA1*0202-DPB1*0501 | HLA-DQA1*0302-DQB1*0319 |
| 1170 |  | HLA-DPA1*0202-DPB1*0601 | HLA-DQA1*0302-DQB1*0320 |
| 1171 |  | HLA-DPA1*0202-DPB1*0701 | HLA-DQA1*0302-DQB1*0321 |
| 1172 |  | HLA-DPA1*0202-DPB1*0801 | HLA-DQA1*0302-DQB1*0322 |
| 1173 |  | HLA-DPA1*0202-DPB1*0901 | HLA-DQA1*0302-DQB1*0323 |
| 1174 |  | HLA-DPA1*0202-DPB1*1001 | HLA-DQA1*0302-DQB1*0324 |
| 1175 |  | HLA-DPA1*0202-DPB1*1101 | HLA-DQA1*0302-DQB1*0325 |
| 1176 |  | HLA-DPA1*0202-DPB1*1101 | HLA-DQA1*0302-DQB1*0326 |
| 1177 |  | HLA-DPA1*0202-DPB1*1201 | HLA-DQA1*0302-DQB1*0327 |
| 1178 |  | HLA-DPA1*0202-DPB1*1301 | HLA-DQA1*0302-DQB1*0328 |
| 1179 |  | HLA-DPA1*0202-DPB1*1401 | HLA-DQA1*0302-DQB1*0329 |
| 1180 |  | HLA-DPA1*0202-DPB1*1501 | HLA-DQA1*0302-DQB1*0330 |
| 1181 |  | HLA-DPA1*0202-DPB1*1601 | HLA-DQA1*0302-DQB1*0331 |
| 1182 |  | HLA-DPA1*0202-DPB1*1701 | HLA-DQA1*0302-DQB1*0332 |
| 1183 |  | HLA-DPA1*0202-DPB1*1801 | HLA-DQA1*0302-DQB1*0333 |
| 1184 |  | HLA-DPA1*0202-DPB1*1901 | HLA-DQA1*0302-DQB1*0334 |
| 1185 |  | HLA-DPA1*0202-DPB1*2101 | HLA-DQA1*0302-DQB1*0335 |
| 1186 |  | HLA-DPA1*0202-DPB1*2201 | HLA-DQA1*0302-DQB1*0336 |
| 1187 |  | HLA-DPA1*0202-DPB1*2301 | HLA-DQA1*0302-DQB1*0337 |
| 1188 |  | HLA-DPA1*0202-DPB1*2401 | HLA-DQA1*0302-DQB1*0338 |
| 1189 |  | HLA-DPA1*0202-DPB1*2501 | HLA-DQA1*0302-DQB1*0401 |
| 1190 |  | HLA-DPA1*0202-DPB1*2601 | HLA-DQA1*0302-DQB1*0402 |
| 1191 |  | HLA-DPA1*0202-DPB1*2701 | HLA-DQA1*0302-DQB1*0403 |
| 1192 |  | HLA-DPA1*0202-DPB1*2801 | HLA-DQA1*0302-DQB1*0404 |
| 1193 |  | HLA-DPA1*0202-DPB1*2901 | HLA-DQA1*0302-DQB1*0405 |
| 1194 |  | HLA-DPA1*0202-DPB1*3001 | HLA-DQA1*0302-DQB1*0406 |
| 1195 |  | HLA-DPA1*0202-DPB1*1301 | HLA-DQA1*0302-DQB1*0407 |
| 1196 |  | HLA-DPA1*0202-DPB1*3101 | HLA-DQA1*0302-DQB1*0408 |
| 1197 |  | HLA-DPA1*0202-DPB1*3201 | HLA-DQA1*0302-DQB1*0501 |
| 1198 |  | HLA-DPA1*0202-DPB1*3301 | HLA-DQA1*0302-DQB1*0502 |
| 1199 |  | HLA-DPA1*0202-DPB1*3401 | HLA-DQA1*0302-DQB1*0503 |
| 1200 |  | HLA-DPA1*0202-DPB1*1401 | HLA-DQA1*0302-DQB1*0505 |
| 1201 |  | HLA-DPA1*0202-DPB1*1501 | HLA-DQA1*0302-DQB1*0506 |
| 1202 |  | HLA-DPA1*0202-DPB1*1601 | HLA-DQA1*0302-DQB1*0507 |
| 1203 |  | HLA-DPA1*0202-DPB1*1701 | HLA-DQA1*0302-DQB1*0508 |
| 1204 |  | HLA-DPA1*0202-DPB1*1801 | HLA-DQA1*0302-DQB1*0509 |
| 1205 |  | HLA-DPA1*0202-DPB1*1901 | HLA-DQA1*0302-DQB1*0510 |
| 1206 |  | HLA-DPA1*0202-DPB1*2001 | HLA-DQA1*0302-DQB1*0511 |
| 1207 |  | HLA-DPA1*0202-DPB1*2101 | HLA-DQA1*0302-DQB1*0512 |
| 1208 |  | HLA-DPA1*0202-DPB1*2201 | HLA-DQA1*0302-DQB1*0513 |
| 1209 |  | HLA-DPA1*0202-DPB1*2301 | HLA-DQA1*0302-DQB1*0514 |
| 1210 |  | HLA-DPA1*0202-DPB1*2401 | HLA-DQA1*0302-DQB1*0601 |
| 1211 |  | HLA-DPA1*0202-DPB1*2501 | HLA-DQA1*0302-DQB1*0602 |
| 1212 |  | HLA-DPA1*0202-DPB1*2601 | HLA-DQA1*0302-DQB1*0603 |
| 1213 |  | HLA-DPA1*0202-DPB1*2701 | HLA-DQA1*0302-DQB1*0604 |
| 1214 |  | HLA-DPA1*0202-DPB1*2801 | HLA-DQA1*0302-DQB1*0607 |
| 1215 |  | HLA-DPA1*0202-DPB1*2901 | HLA-DQA1*0302-DQB1*0608 |
| 1216 |  | HLA-DPA1*0202-DPB1*3001 | HLA-DQA1*0302-DQB1*0609 |
| 1217 |  | HLA-DPA1*0202-DPB1*3101 | HLA-DQA1*0302-DQB1*0610 |
| 1218 |  | HLA-DPA1*0202-DPB1*3201 | HLA-DQA1*0302-DQB1*0611 |
| 1219 |  | HLA-DPA1*0202-DPB1*3301 | HLA-DQA1*0302-DQB1*0612 |
| 1220 |  | HLA-DPA1*0202-DPB1*3401 | HLA-DQA1*0302-DQB1*0614 |
| 1221 |  | HLA-DPA1*0202-DPB1*3501 | HLA-DQA1*0302-DQB1*0615 |
| 1222 |  | HLA-DPA1*0202-DPB1*3601 | HLA-DQA1*0302-DQB1*0616 |
| 1223 |  | HLA-DPA1*0202-DPB1*3701 | HLA-DQA1*0302-DQB1*0617 |
| 1224 |  | HLA-DPA1*0202-DPB1*3801 | HLA-DQA1*0302-DQB1*0618 |
| 1225 |  | HLA-DPA1*0202-DPB1*3901 | HLA-DQA1*0302-DQB1*0619 |
| 1226 |  | HLA-DPA1*0202-DPB1*4001 | HLA-DQA1*0302-DQB1*0621 |
| 1227 |  | HLA-DPA1*0202-DPB1*4101 | HLA-DQA1*0302-DQB1*0622 |
| 1228 |  | HLA-DPA1*0202-DPB1*4401 | HLA-DQA1*0302-DQB1*0623 |
| 1229 |  | HLA-DPA1*0202-DPB1*4501 | HLA-DQA1*0302-DQB1*0624 |
| 1230 |  | HLA-DPA1*0202-DPB1*4601 | HLA-DQA1*0302-DQB1*0625 |
| 1231 |  | HLA-DPA1*0202-DPB1*4701 | HLA-DQA1*0302-DQB1*0627 |
| 1232 |  | HLA-DPA1*0202-DPB1*4801 | HLA-DQA1*0302-DQB1*0628 |
| 1233 |  | HLA-DPA1*0202-DPB1*4901 | HLA-DQA1*0302-DQB1*0629 |
| 1234 |  | HLA-DPA1*0202-DPB1*5001 | HLA-DQA1*0302-DQB1*0630 |
| 1235 |  | HLA-DPA1*0202-DPB1*5101 | HLA-DQA1*0302-DQB1*0631 |
| 1236 |  | HLA-DPA1*0202-DPB1*5201 | HLA-DQA1*0302-DQB1*0632 |
| 1237 |  | HLA-DPA1*0202-DPB1*5301 | HLA-DQA1*0302-DQB1*0633 |
| 1238 |  | HLA-DPA1*0202-DPB1*5401 | HLA-DQA1*0302-DQB1*0634 |
| 1239 |  | HLA-DPA1*0202-DPB1*5501 | HLA-DQA1*0302-DQB1*0635 |
| 1240 |  | HLA-DPA1*0202-DPB1*5601 | HLA-DQA1*0302-DQB1*0636 |
| 1241 |  | HLA-DPA1*0202-DPB1*5801 | HLA-DQA1*0302-DQB1*0637 |
| 1242 |  | HLA-DPA1*0202-DPB1*5901 | HLA-DQA1*0302-DQB1*0638 |
| 1243 |  | HLA-DPA1*0202-DPB1*6001 | HLA-DQA1*0302-DQB1*0639 |
| 1244 |  | HLA-DPA1*0202-DPB1*6201 | HLA-DQA1*0302-DQB1*0640 |
| 1245 |  | HLA-DPA1*0202-DPB1*6301 | HLA-DQA1*0302-DQB1*0641 |
| 1246 |  | HLA-DPA1*0202-DPB1*6501 | HLA-DQA1*0302-DQB1*0642 |
| 1247 |  | HLA-DPA1*0202-DPB1*6601 | HLA-DQA1*0302-DQB1*0643 |
| 1248 |  | HLA-DPA1*0202-DPB1*6701 | HLA-DQA1*0302-DQB1*0644 |
| 1249 |  | HLA-DPA1*0202-DPB1*6801 | HLA-DQA1*0303-DQB1*0201 |
| 1250 |  | HLA-DPA1*0202-DPB1*6901 | HLA-DQA1*0303-DQB1*0202 |
| 1251 |  | HLA-DPA1*0202-DPB1*7001 | HLA-DQA1*0303-DQB1*0203 |
| 1252 |  | HLA-DPA1*0202-DPB1*7101 | HLA-DQA1*0303-DQB1*0204 |
| 1253 |  | HLA-DPA1*0202-DPB1*7201 | HLA-DQA1*0303-DQB1*0205 |
| 1254 |  | HLA-DPA1*0202-DPB1*7301 | HLA-DQA1*0303-DQB1*0206 |
| 1255 |  | HLA-DPA1*0202-DPB1*7401 | HLA-DQA1*0303-DQB1*0301 |
| 1256 |  | HLA-DPA1*0202-DPB1*7501 | HLA-DQA1*0303-DQB1*0302 |
| 1257 |  | HLA-DPA1*0202-DPB1*7601 | HLA-DQA1*0303-DQB1*0303 |
| 1258 |  | HLA-DPA1*0202-DPB1*7701 | HLA-DQA1*0303-DQB1*0304 |
| 1259 |  | HLA-DPA1*0202-DPB1*7801 | HLA-DQA1*0303-DQB1*0305 |
| 1260 |  | HLA-DPA1*0202-DPB1*7901 | HLA-DQA1*0303-DQB1*0306 |
| 1261 |  | HLA-DPA1*0202-DPB1*8001 | HLA-DQA1*0303-DQB1*0307 |
| 1262 |  | HLA-DPA1*0202-DPB1*8101 | HLA-DQA1*0303-DQB1*0308 |
| 1263 |  | HLA-DPA1*0202-DPB1*8201 | HLA-DQA1*0303-DQB1*0309 |
| 1264 |  | HLA-DPA1*0202-DPB1*8301 | HLA-DQA1*0303-DQB1*0310 |
| 1265 |  | HLA-DPA1*0202-DPB1*8401 | HLA-DQA1*0303-DQB1*0311 |
| 1266 |  | HLA-DPA1*0202-DPB1*8501 | HLA-DQA1*0303-DQB1*0312 |
| 1267 |  | HLA-DPA1*0202-DPB1*8601 | HLA-DQA1*0303-DQB1*0313 |
| 1268 |  | HLA-DPA1*0202-DPB1*8701 | HLA-DQA1*0303-DQB1*0314 |
| 1269 |  | HLA-DPA1*0202-DPB1*8801 | HLA-DQA1*0303-DQB1*0315 |
| 1270 |  | HLA-DPA1*0202-DPB1*8901 | HLA-DQA1*0303-DQB1*0316 |
| 1271 |  | HLA-DPA1*0202-DPB1*9001 | HLA-DQA1*0303-DQB1*0317 |
| 1272 |  | HLA-DPA1*0202-DPB1*9101 | HLA-DQA1*0303-DQB1*0318 |
| 1273 |  | HLA-DPA1*0202-DPB1*9201 | HLA-DQA1*0303-DQB1*0319 |
| 1274 |  | HLA-DPA1*0202-DPB1*9301 | HLA-DQA1*0303-DQB1*0320 |
| 1275 |  | HLA-DPA1*0202-DPB1*9401 | HLA-DQA1*0303-DQB1*0321 |
| 1276 |  | HLA-DPA1*0202-DPB1*9501 | HLA-DQA1*0303-DQB1*0322 |
| 1277 |  | HLA-DPA1*0202-DPB1*9601 | HLA-DQA1*0303-DQB1*0323 |
| 1278 |  | HLA-DPA1*0202-DPB1*9701 | HLA-DQA1*0303-DQB1*0324 |
| 1279 |  | HLA-DPA1*0202-DPB1*9801 | HLA-DQA1*0303-DQB1*0325 |
| 1280 |  | HLA-DPA1*0202-DPB1*9901 | HLA-DQA1*0303-DQB1*0326 |
| 1281 |  | HLA-DPA1*0203-DPB1*0101 | HLA-DQA1*0303-DQB1*0327 |
| 1282 |  | HLA-DPA1*0203-DPB1*0201 | HLA-DQA1*0303-DQB1*0328 |
| 1283 |  | HLA-DPA1*0203-DPB1*0202 | HLA-DQA1*0303-DQB1*0329 |
| 1284 |  | HLA-DPA1*0203-DPB1*0301 | HLA-DQA1*0303-DQB1*0330 |
| 1285 |  | HLA-DPA1*0203-DPB1*0401 | HLA-DQA1*0303-DQB1*0331 |
| 1286 |  | HLA-DPA1*0203-DPB1*0402 | HLA-DQA1*0303-DQB1*0332 |
| 1287 |  | HLA-DPA1*0203-DPB1*0501 | HLA-DQA1*0303-DQB1*0333 |
| 1288 |  | HLA-DPA1*0203-DPB1*0601 | HLA-DQA1*0303-DQB1*0334 |
| 1289 |  | HLA-DPA1*0203-DPB1*0801 | HLA-DQA1*0303-DQB1*0335 |
| 1290 |  | HLA-DPA1*0203-DPB1*0901 | HLA-DQA1*0303-DQB1*0336 |
| 1291 |  | HLA-DPA1*0203-DPB1*0001 | HLA-DQA1*0303-DQB1*0337 |
| 1292 |  | HLA-DPA1*0203-DPB1*1001 | HLA-DQA1*0303-DQB1*0338 |
| 1293 |  | HLA-DPA1*0203-DPB1*0101 | HLA-DQA1*0303-DQB1*0401 |
| 1294 |  | HLA-DPA1*0203-DPB1*0201 | HLA-DQA1*0303-DQB1*0402 |
| 1295 |  | HLA-DPA1*0203-DPB1*0301 | HLA-DQA1*0303-DQB1*0403 |
| 1296 |  | HLA-DPA1*0203-DPB1*0401 | HLA-DQA1*0303-DQB1*0404 |
| 1297 |  | HLA-DPA1*0203-DPB1*0501 | HLA-DQA1*0303-DQB1*0405 |
| 1298 |  | HLA-DPA1*0203-DPB1*0601 | HLA-DQA1*0303-DQB1*0406 |
| 1299 |  | HLA-DPA1*0203-DPB1*0701 | HLA-DQA1*0303-DQB1*0407 |
| 1300 |  | HLA-DPA1*0203-DPB1*0801 | HLA-DQA1*0303-DQB1*0408 |
| 1301 |  | HLA-DPA1*0203-DPB1*0901 | HLA-DQA1*0303-DQB1*0501 |
| 1302 |  | HLA-DPA1*0203-DPB1*1001 | HLA-DQA1*0303-DQB1*0502 |
| 1303 |  | HLA-DPA1*0203-DPB1*1101 | HLA-DQA1*0303-DQB1*0503 |
| 1304 |  | HLA-DPA1*0203-DPB1*1101 | HLA-DQA1*0303-DQB1*0505 |
| 1305 |  | HLA-DPA1*0203-DPB1*1201 | HLA-DQA1*0303-DQB1*0506 |
| 1306 |  | HLA-DPA1*0203-DPB1*1301 | HLA-DQA1*0303-DQB1*0507 |
| 1307 |  | HLA-DPA1*0203-DPB1*1401 | HLA-DQA1*0303-DQB1*0508 |
| 1308 |  | HLA-DPA1*0203-DPB1*1501 | HLA-DQA1*0303-DQB1*0509 |
| 1309 |  | HLA-DPA1*0203-DPB1*1601 | HLA-DQA1*0303-DQB1*0510 |
| 1310 |  | HLA-DPA1*0203-DPB1*1701 | HLA-DQA1*0303-DQB1*0511 |
| 1311 |  | HLA-DPA1*0203-DPB1*1801 | HLA-DQA1*0303-DQB1*0512 |
| 1312 |  | HLA-DPA1*0203-DPB1*1901 | HLA-DQA1*0303-DQB1*0513 |
| 1313 |  | HLA-DPA1*0203-DPB1*2101 | HLA-DQA1*0303-DQB1*0514 |
| 1314 |  | HLA-DPA1*0203-DPB1*2201 | HLA-DQA1*0303-DQB1*0601 |
| 1315 |  | HLA-DPA1*0203-DPB1*2301 | HLA-DQA1*0303-DQB1*0602 |
| 1316 |  | HLA-DPA1*0203-DPB1*2401 | HLA-DQA1*0303-DQB1*0603 |
| 1317 |  | HLA-DPA1*0203-DPB1*2501 | HLA-DQA1*0303-DQB1*0604 |
| 1318 |  | HLA-DPA1*0203-DPB1*2601 | HLA-DQA1*0303-DQB1*0607 |
| 1319 |  | HLA-DPA1*0203-DPB1*2701 | HLA-DQA1*0303-DQB1*0608 |
| 1320 |  | HLA-DPA1*0203-DPB1*2801 | HLA-DQA1*0303-DQB1*0609 |
| 1321 |  | HLA-DPA1*0203-DPB1*2901 | HLA-DQA1*0303-DQB1*0610 |
| 1322 |  | HLA-DPA1*0203-DPB1*3001 | HLA-DQA1*0303-DQB1*0611 |
| 1323 |  | HLA-DPA1*0203-DPB1*1301 | HLA-DQA1*0303-DQB1*0612 |
| 1324 |  | HLA-DPA1*0203-DPB1*3101 | HLA-DQA1*0303-DQB1*0614 |
| 1325 |  | HLA-DPA1*0203-DPB1*3201 | HLA-DQA1*0303-DQB1*0615 |
| 1326 |  | HLA-DPA1*0203-DPB1*3301 | HLA-DQA1*0303-DQB1*0616 |
| 1327 |  | HLA-DPA1*0203-DPB1*3401 | HLA-DQA1*0303-DQB1*0617 |
| 1328 |  | HLA-DPA1*0203-DPB1*1401 | HLA-DQA1*0303-DQB1*0618 |
| 1329 |  | HLA-DPA1*0203-DPB1*1501 | HLA-DQA1*0303-DQB1*0619 |
| 1330 |  | HLA-DPA1*0203-DPB1*1601 | HLA-DQA1*0303-DQB1*0621 |
| 1331 |  | HLA-DPA1*0203-DPB1*1701 | HLA-DQA1*0303-DQB1*0622 |
| 1332 |  | HLA-DPA1*0203-DPB1*1801 | HLA-DQA1*0303-DQB1*0623 |
| 1333 |  | HLA-DPA1*0203-DPB1*1901 | HLA-DQA1*0303-DQB1*0624 |
| 1334 |  | HLA-DPA1*0203-DPB1*2001 | HLA-DQA1*0303-DQB1*0625 |
| 1335 |  | HLA-DPA1*0203-DPB1*2101 | HLA-DQA1*0303-DQB1*0627 |
| 1336 |  | HLA-DPA1*0203-DPB1*2201 | HLA-DQA1*0303-DQB1*0628 |
| 1337 |  | HLA-DPA1*0203-DPB1*2301 | HLA-DQA1*0303-DQB1*0629 |
| 1338 |  | HLA-DPA1*0203-DPB1*2401 | HLA-DQA1*0303-DQB1*0630 |
| 1339 |  | HLA-DPA1*0203-DPB1*2501 | HLA-DQA1*0303-DQB1*0631 |
| 1340 |  | HLA-DPA1*0203-DPB1*2601 | HLA-DQA1*0303-DQB1*0632 |
| 1341 |  | HLA-DPA1*0203-DPB1*2701 | HLA-DQA1*0303-DQB1*0633 |
| 1342 |  | HLA-DPA1*0203-DPB1*2801 | HLA-DQA1*0303-DQB1*0634 |
| 1343 |  | HLA-DPA1*0203-DPB1*2901 | HLA-DQA1*0303-DQB1*0635 |
| 1344 |  | HLA-DPA1*0203-DPB1*3001 | HLA-DQA1*0303-DQB1*0636 |
| 1345 |  | HLA-DPA1*0203-DPB1*3101 | HLA-DQA1*0303-DQB1*0637 |
| 1346 |  | HLA-DPA1*0203-DPB1*3201 | HLA-DQA1*0303-DQB1*0638 |
| 1347 |  | HLA-DPA1*0203-DPB1*3301 | HLA-DQA1*0303-DQB1*0639 |
| 1348 |  | HLA-DPA1*0203-DPB1*3401 | HLA-DQA1*0303-DQB1*0640 |
| 1349 |  | HLA-DPA1*0203-DPB1*3501 | HLA-DQA1*0303-DQB1*0641 |
| 1350 |  | HLA-DPA1*0203-DPB1*3601 | HLA-DQA1*0303-DQB1*0642 |
| 1351 |  | HLA-DPA1*0203-DPB1*3701 | HLA-DQA1*0303-DQB1*0643 |
| 1352 |  | HLA-DPA1*0203-DPB1*3801 | HLA-DQA1*0303-DQB1*0644 |
| 1353 |  | HLA-DPA1*0203-DPB1*3901 | HLA-DQA1*0401-DQB1*0201 |
| 1354 |  | HLA-DPA1*0203-DPB1*4001 | HLA-DQA1*0401-DQB1*0202 |
| 1355 |  | HLA-DPA1*0203-DPB1*4101 | HLA-DQA1*0401-DQB1*0203 |
| 1356 |  | HLA-DPA1*0203-DPB1*4401 | HLA-DQA1*0401-DQB1*0204 |
| 1357 |  | HLA-DPA1*0203-DPB1*4501 | HLA-DQA1*0401-DQB1*0205 |
| 1358 |  | HLA-DPA1*0203-DPB1*4601 | HLA-DQA1*0401-DQB1*0206 |
| 1359 |  | HLA-DPA1*0203-DPB1*4701 | HLA-DQA1*0401-DQB1*0301 |
| 1360 |  | HLA-DPA1*0203-DPB1*4801 | HLA-DQA1*0401-DQB1*0302 |
| 1361 |  | HLA-DPA1*0203-DPB1*4901 | HLA-DQA1*0401-DQB1*0303 |
| 1362 |  | HLA-DPA1*0203-DPB1*5001 | HLA-DQA1*0401-DQB1*0304 |
| 1363 |  | HLA-DPA1*0203-DPB1*5101 | HLA-DQA1*0401-DQB1*0305 |
| 1364 |  | HLA-DPA1*0203-DPB1*5201 | HLA-DQA1*0401-DQB1*0306 |
| 1365 |  | HLA-DPA1*0203-DPB1*5301 | HLA-DQA1*0401-DQB1*0307 |
| 1366 |  | HLA-DPA1*0203-DPB1*5401 | HLA-DQA1*0401-DQB1*0308 |
| 1367 |  | HLA-DPA1*0203-DPB1*5501 | HLA-DQA1*0401-DQB1*0309 |
| 1368 |  | HLA-DPA1*0203-DPB1*5601 | HLA-DQA1*0401-DQB1*0310 |
| 1369 |  | HLA-DPA1*0203-DPB1*5801 | HLA-DQA1*0401-DQB1*0311 |
| 1370 |  | HLA-DPA1*0203-DPB1*5901 | HLA-DQA1*0401-DQB1*0312 |
| 1371 |  | HLA-DPA1*0203-DPB1*6001 | HLA-DQA1*0401-DQB1*0313 |
| 1372 |  | HLA-DPA1*0203-DPB1*6201 | HLA-DQA1*0401-DQB1*0314 |
| 1373 |  | HLA-DPA1*0203-DPB1*6301 | HLA-DQA1*0401-DQB1*0315 |
| 1374 |  | HLA-DPA1*0203-DPB1*6501 | HLA-DQA1*0401-DQB1*0316 |
| 1375 |  | HLA-DPA1*0203-DPB1*6601 | HLA-DQA1*0401-DQB1*0317 |
| 1376 |  | HLA-DPA1*0203-DPB1*6701 | HLA-DQA1*0401-DQB1*0318 |
| 1377 |  | HLA-DPA1*0203-DPB1*6801 | HLA-DQA1*0401-DQB1*0319 |
| 1378 |  | HLA-DPA1*0203-DPB1*6901 | HLA-DQA1*0401-DQB1*0320 |
| 1379 |  | HLA-DPA1*0203-DPB1*7001 | HLA-DQA1*0401-DQB1*0321 |
| 1380 |  | HLA-DPA1*0203-DPB1*7101 | HLA-DQA1*0401-DQB1*0322 |
| 1381 |  | HLA-DPA1*0203-DPB1*7201 | HLA-DQA1*0401-DQB1*0323 |
| 1382 |  | HLA-DPA1*0203-DPB1*7301 | HLA-DQA1*0401-DQB1*0324 |
| 1383 |  | HLA-DPA1*0203-DPB1*7401 | HLA-DQA1*0401-DQB1*0325 |
| 1384 |  | HLA-DPA1*0203-DPB1*7501 | HLA-DQA1*0401-DQB1*0326 |
| 1385 |  | HLA-DPA1*0203-DPB1*7601 | HLA-DQA1*0401-DQB1*0327 |
| 1386 |  | HLA-DPA1*0203-DPB1*7701 | HLA-DQA1*0401-DQB1*0328 |
| 1387 |  | HLA-DPA1*0203-DPB1*7801 | HLA-DQA1*0401-DQB1*0329 |
| 1388 |  | HLA-DPA1*0203-DPB1*7901 | HLA-DQA1*0401-DQB1*0330 |
| 1389 |  | HLA-DPA1*0203-DPB1*8001 | HLA-DQA1*0401-DQB1*0331 |
| 1390 |  | HLA-DPA1*0203-DPB1*8101 | HLA-DQA1*0401-DQB1*0332 |
| 1391 |  | HLA-DPA1*0203-DPB1*8201 | HLA-DQA1*0401-DQB1*0333 |
| 1392 |  | HLA-DPA1*0203-DPB1*8301 | HLA-DQA1*0401-DQB1*0334 |
| 1393 |  | HLA-DPA1*0203-DPB1*8401 | HLA-DQA1*0401-DQB1*0335 |
| 1394 |  | HLA-DPA1*0203-DPB1*8501 | HLA-DQA1*0401-DQB1*0336 |
| 1395 |  | HLA-DPA1*0203-DPB1*8601 | HLA-DQA1*0401-DQB1*0337 |
| 1396 |  | HLA-DPA1*0203-DPB1*8701 | HLA-DQA1*0401-DQB1*0338 |
| 1397 |  | HLA-DPA1*0203-DPB1*8801 | HLA-DQA1*0401-DQB1*0401 |
| 1398 |  | HLA-DPA1*0203-DPB1*8901 | HLA-DQA1*0401-DQB1*0402 |
| 1399 |  | HLA-DPA1*0203-DPB1*9001 | HLA-DQA1*0401-DQB1*0403 |
| 1400 |  | HLA-DPA1*0203-DPB1*9101 | HLA-DQA1*0401-DQB1*0404 |
| 1401 |  | HLA-DPA1*0203-DPB1*9201 | HLA-DQA1*0401-DQB1*0405 |
| 1402 |  | HLA-DPA1*0203-DPB1*9301 | HLA-DQA1*0401-DQB1*0406 |
| 1403 |  | HLA-DPA1*0203-DPB1*9401 | HLA-DQA1*0401-DQB1*0407 |
| 1404 |  | HLA-DPA1*0203-DPB1*9501 | HLA-DQA1*0401-DQB1*0408 |
| 1405 |  | HLA-DPA1*0203-DPB1*9601 | HLA-DQA1*0401-DQB1*0501 |
| 1406 |  | HLA-DPA1*0203-DPB1*9701 | HLA-DQA1*0401-DQB1*0502 |
| 1407 |  | HLA-DPA1*0203-DPB1*9801 | HLA-DQA1*0401-DQB1*0503 |
| 1408 |  | HLA-DPA1*0203-DPB1*9901 | HLA-DQA1*0401-DQB1*0505 |
| 1409 |  | HLA-DPA1*0204-DPB1*0101 | HLA-DQA1*0401-DQB1*0506 |
| 1410 |  | HLA-DPA1*0204-DPB1*0201 | HLA-DQA1*0401-DQB1*0507 |
| 1411 |  | HLA-DPA1*0204-DPB1*0202 | HLA-DQA1*0401-DQB1*0508 |
| 1412 |  | HLA-DPA1*0204-DPB1*0301 | HLA-DQA1*0401-DQB1*0509 |
| 1413 |  | HLA-DPA1*0204-DPB1*0401 | HLA-DQA1*0401-DQB1*0510 |
| 1414 |  | HLA-DPA1*0204-DPB1*0402 | HLA-DQA1*0401-DQB1*0511 |
| 1415 |  | HLA-DPA1*0204-DPB1*0501 | HLA-DQA1*0401-DQB1*0512 |
| 1416 |  | HLA-DPA1*0204-DPB1*0601 | HLA-DQA1*0401-DQB1*0513 |
| 1417 |  | HLA-DPA1*0204-DPB1*0801 | HLA-DQA1*0401-DQB1*0514 |
| 1418 |  | HLA-DPA1*0204-DPB1*0901 | HLA-DQA1*0401-DQB1*0601 |
| 1419 |  | HLA-DPA1*0204-DPB1*0001 | HLA-DQA1*0401-DQB1*0602 |
| 1420 |  | HLA-DPA1*0204-DPB1*1001 | HLA-DQA1*0401-DQB1*0603 |
| 1421 |  | HLA-DPA1*0204-DPB1*0101 | HLA-DQA1*0401-DQB1*0604 |
| 1422 |  | HLA-DPA1*0204-DPB1*0201 | HLA-DQA1*0401-DQB1*0607 |
| 1423 |  | HLA-DPA1*0204-DPB1*0301 | HLA-DQA1*0401-DQB1*0608 |
| 1424 |  | HLA-DPA1*0204-DPB1*0401 | HLA-DQA1*0401-DQB1*0609 |
| 1425 |  | HLA-DPA1*0204-DPB1*0501 | HLA-DQA1*0401-DQB1*0610 |
| 1426 |  | HLA-DPA1*0204-DPB1*0601 | HLA-DQA1*0401-DQB1*0611 |
| 1427 |  | HLA-DPA1*0204-DPB1*0701 | HLA-DQA1*0401-DQB1*0612 |
| 1428 |  | HLA-DPA1*0204-DPB1*0801 | HLA-DQA1*0401-DQB1*0614 |
| 1429 |  | HLA-DPA1*0204-DPB1*0901 | HLA-DQA1*0401-DQB1*0615 |
| 1430 |  | HLA-DPA1*0204-DPB1*1001 | HLA-DQA1*0401-DQB1*0616 |
| 1431 |  | HLA-DPA1*0204-DPB1*1101 | HLA-DQA1*0401-DQB1*0617 |
| 1432 |  | HLA-DPA1*0204-DPB1*1101 | HLA-DQA1*0401-DQB1*0618 |
| 1433 |  | HLA-DPA1*0204-DPB1*1201 | HLA-DQA1*0401-DQB1*0619 |
| 1434 |  | HLA-DPA1*0204-DPB1*1301 | HLA-DQA1*0401-DQB1*0621 |
| 1435 |  | HLA-DPA1*0204-DPB1*1401 | HLA-DQA1*0401-DQB1*0622 |
| 1436 |  | HLA-DPA1*0204-DPB1*1501 | HLA-DQA1*0401-DQB1*0623 |
| 1437 |  | HLA-DPA1*0204-DPB1*1601 | HLA-DQA1*0401-DQB1*0624 |
| 1438 |  | HLA-DPA1*0204-DPB1*1701 | HLA-DQA1*0401-DQB1*0625 |
| 1439 |  | HLA-DPA1*0204-DPB1*1801 | HLA-DQA1*0401-DQB1*0627 |
| 1440 |  | HLA-DPA1*0204-DPB1*1901 | HLA-DQA1*0401-DQB1*0628 |
| 1441 |  | HLA-DPA1*0204-DPB1*2101 | HLA-DQA1*0401-DQB1*0629 |
| 1442 |  | HLA-DPA1*0204-DPB1*2201 | HLA-DQA1*0401-DQB1*0630 |
| 1443 |  | HLA-DPA1*0204-DPB1*2301 | HLA-DQA1*0401-DQB1*0631 |
| 1444 |  | HLA-DPA1*0204-DPB1*2401 | HLA-DQA1*0401-DQB1*0632 |
| 1445 |  | HLA-DPA1*0204-DPB1*2501 | HLA-DQA1*0401-DQB1*0633 |
| 1446 |  | HLA-DPA1*0204-DPB1*2601 | HLA-DQA1*0401-DQB1*0634 |
| 1447 |  | HLA-DPA1*0204-DPB1*2701 | HLA-DQA1*0401-DQB1*0635 |
| 1448 |  | HLA-DPA1*0204-DPB1*2801 | HLA-DQA1*0401-DQB1*0636 |
| 1449 |  | HLA-DPA1*0204-DPB1*2901 | HLA-DQA1*0401-DQB1*0637 |
| 1450 |  | HLA-DPA1*0204-DPB1*3001 | HLA-DQA1*0401-DQB1*0638 |
| 1451 |  | HLA-DPA1*0204-DPB1*1301 | HLA-DQA1*0401-DQB1*0639 |
| 1452 |  | HLA-DPA1*0204-DPB1*3101 | HLA-DQA1*0401-DQB1*0640 |
| 1453 |  | HLA-DPA1*0204-DPB1*3201 | HLA-DQA1*0401-DQB1*0641 |
| 1454 |  | HLA-DPA1*0204-DPB1*3301 | HLA-DQA1*0401-DQB1*0642 |
| 1455 |  | HLA-DPA1*0204-DPB1*3401 | HLA-DQA1*0401-DQB1*0643 |
| 1456 |  | HLA-DPA1*0204-DPB1*1401 | HLA-DQA1*0401-DQB1*0644 |
| 1457 |  | HLA-DPA1*0204-DPB1*1501 | HLA-DQA1*0402-DQB1*0201 |
| 1458 |  | HLA-DPA1*0204-DPB1*1601 | HLA-DQA1*0402-DQB1*0202 |
| 1459 |  | HLA-DPA1*0204-DPB1*1701 | HLA-DQA1*0402-DQB1*0203 |
| 1460 |  | HLA-DPA1*0204-DPB1*1801 | HLA-DQA1*0402-DQB1*0204 |
| 1461 |  | HLA-DPA1*0204-DPB1*1901 | HLA-DQA1*0402-DQB1*0205 |
| 1462 |  | HLA-DPA1*0204-DPB1*2001 | HLA-DQA1*0402-DQB1*0206 |
| 1463 |  | HLA-DPA1*0204-DPB1*2101 | HLA-DQA1*0402-DQB1*0301 |
| 1464 |  | HLA-DPA1*0204-DPB1*2201 | HLA-DQA1*0402-DQB1*0302 |
| 1465 |  | HLA-DPA1*0204-DPB1*2301 | HLA-DQA1*0402-DQB1*0303 |
| 1466 |  | HLA-DPA1*0204-DPB1*2401 | HLA-DQA1*0402-DQB1*0304 |
| 1467 |  | HLA-DPA1*0204-DPB1*2501 | HLA-DQA1*0402-DQB1*0305 |
| 1468 |  | HLA-DPA1*0204-DPB1*2601 | HLA-DQA1*0402-DQB1*0306 |
| 1469 |  | HLA-DPA1*0204-DPB1*2701 | HLA-DQA1*0402-DQB1*0307 |
| 1470 |  | HLA-DPA1*0204-DPB1*2801 | HLA-DQA1*0402-DQB1*0308 |
| 1471 |  | HLA-DPA1*0204-DPB1*2901 | HLA-DQA1*0402-DQB1*0309 |
| 1472 |  | HLA-DPA1*0204-DPB1*3001 | HLA-DQA1*0402-DQB1*0310 |
| 1473 |  | HLA-DPA1*0204-DPB1*3101 | HLA-DQA1*0402-DQB1*0311 |
| 1474 |  | HLA-DPA1*0204-DPB1*3201 | HLA-DQA1*0402-DQB1*0312 |
| 1475 |  | HLA-DPA1*0204-DPB1*3301 | HLA-DQA1*0402-DQB1*0313 |
| 1476 |  | HLA-DPA1*0204-DPB1*3401 | HLA-DQA1*0402-DQB1*0314 |
| 1477 |  | HLA-DPA1*0204-DPB1*3501 | HLA-DQA1*0402-DQB1*0315 |
| 1478 |  | HLA-DPA1*0204-DPB1*3601 | HLA-DQA1*0402-DQB1*0316 |
| 1479 |  | HLA-DPA1*0204-DPB1*3701 | HLA-DQA1*0402-DQB1*0317 |
| 1480 |  | HLA-DPA1*0204-DPB1*3801 | HLA-DQA1*0402-DQB1*0318 |
| 1481 |  | HLA-DPA1*0204-DPB1*3901 | HLA-DQA1*0402-DQB1*0319 |
| 1482 |  | HLA-DPA1*0204-DPB1*4001 | HLA-DQA1*0402-DQB1*0320 |
| 1483 |  | HLA-DPA1*0204-DPB1*4101 | HLA-DQA1*0402-DQB1*0321 |
| 1484 |  | HLA-DPA1*0204-DPB1*4401 | HLA-DQA1*0402-DQB1*0322 |
| 1485 |  | HLA-DPA1*0204-DPB1*4501 | HLA-DQA1*0402-DQB1*0323 |
| 1486 |  | HLA-DPA1*0204-DPB1*4601 | HLA-DQA1*0402-DQB1*0324 |
| 1487 |  | HLA-DPA1*0204-DPB1*4701 | HLA-DQA1*0402-DQB1*0325 |
| 1488 |  | HLA-DPA1*0204-DPB1*4801 | HLA-DQA1*0402-DQB1*0326 |
| 1489 |  | HLA-DPA1*0204-DPB1*4901 | HLA-DQA1*0402-DQB1*0327 |
| 1490 |  | HLA-DPA1*0204-DPB1*5001 | HLA-DQA1*0402-DQB1*0328 |
| 1491 |  | HLA-DPA1*0204-DPB1*5101 | HLA-DQA1*0402-DQB1*0329 |
| 1492 |  | HLA-DPA1*0204-DPB1*5201 | HLA-DQA1*0402-DQB1*0330 |
| 1493 |  | HLA-DPA1*0204-DPB1*5301 | HLA-DQA1*0402-DQB1*0331 |
| 1494 |  | HLA-DPA1*0204-DPB1*5401 | HLA-DQA1*0402-DQB1*0332 |
| 1495 |  | HLA-DPA1*0204-DPB1*5501 | HLA-DQA1*0402-DQB1*0333 |
| 1496 |  | HLA-DPA1*0204-DPB1*5601 | HLA-DQA1*0402-DQB1*0334 |
| 1497 |  | HLA-DPA1*0204-DPB1*5801 | HLA-DQA1*0402-DQB1*0335 |
| 1498 |  | HLA-DPA1*0204-DPB1*5901 | HLA-DQA1*0402-DQB1*0336 |
| 1499 |  | HLA-DPA1*0204-DPB1*6001 | HLA-DQA1*0402-DQB1*0337 |
| 1500 |  | HLA-DPA1*0204-DPB1*6201 | HLA-DQA1*0402-DQB1*0338 |
| 1501 |  | HLA-DPA1*0204-DPB1*6301 | HLA-DQA1*0402-DQB1*0401 |
| 1502 |  | HLA-DPA1*0204-DPB1*6501 | HLA-DQA1*0402-DQB1*0402 |
| 1503 |  | HLA-DPA1*0204-DPB1*6601 | HLA-DQA1*0402-DQB1*0403 |
| 1504 |  | HLA-DPA1*0204-DPB1*6701 | HLA-DQA1*0402-DQB1*0404 |
| 1505 |  | HLA-DPA1*0204-DPB1*6801 | HLA-DQA1*0402-DQB1*0405 |
| 1506 |  | HLA-DPA1*0204-DPB1*6901 | HLA-DQA1*0402-DQB1*0406 |
| 1507 |  | HLA-DPA1*0204-DPB1*7001 | HLA-DQA1*0402-DQB1*0407 |
| 1508 |  | HLA-DPA1*0204-DPB1*7101 | HLA-DQA1*0402-DQB1*0408 |
| 1509 |  | HLA-DPA1*0204-DPB1*7201 | HLA-DQA1*0402-DQB1*0501 |
| 1510 |  | HLA-DPA1*0204-DPB1*7301 | HLA-DQA1*0402-DQB1*0502 |
| 1511 |  | HLA-DPA1*0204-DPB1*7401 | HLA-DQA1*0402-DQB1*0503 |
| 1512 |  | HLA-DPA1*0204-DPB1*7501 | HLA-DQA1*0402-DQB1*0505 |
| 1513 |  | HLA-DPA1*0204-DPB1*7601 | HLA-DQA1*0402-DQB1*0506 |
| 1514 |  | HLA-DPA1*0204-DPB1*7701 | HLA-DQA1*0402-DQB1*0507 |
| 1515 |  | HLA-DPA1*0204-DPB1*7801 | HLA-DQA1*0402-DQB1*0508 |
| 1516 |  | HLA-DPA1*0204-DPB1*7901 | HLA-DQA1*0402-DQB1*0509 |
| 1517 |  | HLA-DPA1*0204-DPB1*8001 | HLA-DQA1*0402-DQB1*0510 |
| 1518 |  | HLA-DPA1*0204-DPB1*8101 | HLA-DQA1*0402-DQB1*0511 |
| 1519 |  | HLA-DPA1*0204-DPB1*8201 | HLA-DQA1*0402-DQB1*0512 |
| 1520 |  | HLA-DPA1*0204-DPB1*8301 | HLA-DQA1*0402-DQB1*0513 |
| 1521 |  | HLA-DPA1*0204-DPB1*8401 | HLA-DQA1*0402-DQB1*0514 |
| 1522 |  | HLA-DPA1*0204-DPB1*8501 | HLA-DQA1*0402-DQB1*0601 |
| 1523 |  | HLA-DPA1*0204-DPB1*8601 | HLA-DQA1*0402-DQB1*0602 |
| 1524 |  | HLA-DPA1*0204-DPB1*8701 | HLA-DQA1*0402-DQB1*0603 |
| 1525 |  | HLA-DPA1*0204-DPB1*8801 | HLA-DQA1*0402-DQB1*0604 |
| 1526 |  | HLA-DPA1*0204-DPB1*8901 | HLA-DQA1*0402-DQB1*0607 |
| 1527 |  | HLA-DPA1*0204-DPB1*9001 | HLA-DQA1*0402-DQB1*0608 |
| 1528 |  | HLA-DPA1*0204-DPB1*9101 | HLA-DQA1*0402-DQB1*0609 |
| 1529 |  | HLA-DPA1*0204-DPB1*9201 | HLA-DQA1*0402-DQB1*0610 |
| 1530 |  | HLA-DPA1*0204-DPB1*9301 | HLA-DQA1*0402-DQB1*0611 |
| 1531 |  | HLA-DPA1*0204-DPB1*9401 | HLA-DQA1*0402-DQB1*0612 |
| 1532 |  | HLA-DPA1*0204-DPB1*9501 | HLA-DQA1*0402-DQB1*0614 |
| 1533 |  | HLA-DPA1*0204-DPB1*9601 | HLA-DQA1*0402-DQB1*0615 |
| 1534 |  | HLA-DPA1*0204-DPB1*9701 | HLA-DQA1*0402-DQB1*0616 |
| 1535 |  | HLA-DPA1*0204-DPB1*9801 | HLA-DQA1*0402-DQB1*0617 |
| 1536 |  | HLA-DPA1*0204-DPB1*9901 | HLA-DQA1*0402-DQB1*0618 |
| 1537 |  | HLA-DPA1*0301-DPB1*0101 | HLA-DQA1*0402-DQB1*0619 |
| 1538 |  | HLA-DPA1*0301-DPB1*0201 | HLA-DQA1*0402-DQB1*0621 |
| 1539 |  | HLA-DPA1*0301-DPB1*0202 | HLA-DQA1*0402-DQB1*0622 |
| 1540 |  | HLA-DPA1*0301-DPB1*0301 | HLA-DQA1*0402-DQB1*0623 |
| 1541 |  | HLA-DPA1*0301-DPB1*0401 | HLA-DQA1*0402-DQB1*0624 |
| 1542 |  | HLA-DPA1*0301-DPB1*0402 | HLA-DQA1*0402-DQB1*0625 |
| 1543 |  | HLA-DPA1*0301-DPB1*0501 | HLA-DQA1*0402-DQB1*0627 |
| 1544 |  | HLA-DPA1*0301-DPB1*0601 | HLA-DQA1*0402-DQB1*0628 |
| 1545 |  | HLA-DPA1*0301-DPB1*0801 | HLA-DQA1*0402-DQB1*0629 |
| 1546 |  | HLA-DPA1*0301-DPB1*0901 | HLA-DQA1*0402-DQB1*0630 |
| 1547 |  | HLA-DPA1*0301-DPB1*0001 | HLA-DQA1*0402-DQB1*0631 |
| 1548 |  | HLA-DPA1*0301-DPB1*1001 | HLA-DQA1*0402-DQB1*0632 |
| 1549 |  | HLA-DPA1*0301-DPB1*0101 | HLA-DQA1*0402-DQB1*0633 |
| 1550 |  | HLA-DPA1*0301-DPB1*0201 | HLA-DQA1*0402-DQB1*0634 |
| 1551 |  | HLA-DPA1*0301-DPB1*0301 | HLA-DQA1*0402-DQB1*0635 |
| 1552 |  | HLA-DPA1*0301-DPB1*0401 | HLA-DQA1*0402-DQB1*0636 |
| 1553 |  | HLA-DPA1*0301-DPB1*0501 | HLA-DQA1*0402-DQB1*0637 |
| 1554 |  | HLA-DPA1*0301-DPB1*0601 | HLA-DQA1*0402-DQB1*0638 |
| 1555 |  | HLA-DPA1*0301-DPB1*0701 | HLA-DQA1*0402-DQB1*0639 |
| 1556 |  | HLA-DPA1*0301-DPB1*0801 | HLA-DQA1*0402-DQB1*0640 |
| 1557 |  | HLA-DPA1*0301-DPB1*0901 | HLA-DQA1*0402-DQB1*0641 |
| 1558 |  | HLA-DPA1*0301-DPB1*1001 | HLA-DQA1*0402-DQB1*0642 |
| 1559 |  | HLA-DPA1*0301-DPB1*1101 | HLA-DQA1*0402-DQB1*0643 |
| 1560 |  | HLA-DPA1*0301-DPB1*1101 | HLA-DQA1*0402-DQB1*0644 |
| 1561 |  | HLA-DPA1*0301-DPB1*1201 | HLA-DQA1*0404-DQB1*0201 |
| 1562 |  | HLA-DPA1*0301-DPB1*1301 | HLA-DQA1*0404-DQB1*0202 |
| 1563 |  | HLA-DPA1*0301-DPB1*1401 | HLA-DQA1*0404-DQB1*0203 |
| 1564 |  | HLA-DPA1*0301-DPB1*1501 | HLA-DQA1*0404-DQB1*0204 |
| 1565 |  | HLA-DPA1*0301-DPB1*1601 | HLA-DQA1*0404-DQB1*0205 |
| 1566 |  | HLA-DPA1*0301-DPB1*1701 | HLA-DQA1*0404-DQB1*0206 |
| 1567 |  | HLA-DPA1*0301-DPB1*1801 | HLA-DQA1*0404-DQB1*0301 |
| 1568 |  | HLA-DPA1*0301-DPB1*1901 | HLA-DQA1*0404-DQB1*0302 |
| 1569 |  | HLA-DPA1*0301-DPB1*2101 | HLA-DQA1*0404-DQB1*0303 |
| 1570 |  | HLA-DPA1*0301-DPB1*2201 | HLA-DQA1*0404-DQB1*0304 |
| 1571 |  | HLA-DPA1*0301-DPB1*2301 | HLA-DQA1*0404-DQB1*0305 |
| 1572 |  | HLA-DPA1*0301-DPB1*2401 | HLA-DQA1*0404-DQB1*0306 |
| 1573 |  | HLA-DPA1*0301-DPB1*2501 | HLA-DQA1*0404-DQB1*0307 |
| 1574 |  | HLA-DPA1*0301-DPB1*2601 | HLA-DQA1*0404-DQB1*0308 |
| 1575 |  | HLA-DPA1*0301-DPB1*2701 | HLA-DQA1*0404-DQB1*0309 |
| 1576 |  | HLA-DPA1*0301-DPB1*2801 | HLA-DQA1*0404-DQB1*0310 |
| 1577 |  | HLA-DPA1*0301-DPB1*2901 | HLA-DQA1*0404-DQB1*0311 |
| 1578 |  | HLA-DPA1*0301-DPB1*3001 | HLA-DQA1*0404-DQB1*0312 |
| 1579 |  | HLA-DPA1*0301-DPB1*1301 | HLA-DQA1*0404-DQB1*0313 |
| 1580 |  | HLA-DPA1*0301-DPB1*3101 | HLA-DQA1*0404-DQB1*0314 |
| 1581 |  | HLA-DPA1*0301-DPB1*3201 | HLA-DQA1*0404-DQB1*0315 |
| 1582 |  | HLA-DPA1*0301-DPB1*3301 | HLA-DQA1*0404-DQB1*0316 |
| 1583 |  | HLA-DPA1*0301-DPB1*3401 | HLA-DQA1*0404-DQB1*0317 |
| 1584 |  | HLA-DPA1*0301-DPB1*1401 | HLA-DQA1*0404-DQB1*0318 |
| 1585 |  | HLA-DPA1*0301-DPB1*1501 | HLA-DQA1*0404-DQB1*0319 |
| 1586 |  | HLA-DPA1*0301-DPB1*1601 | HLA-DQA1*0404-DQB1*0320 |
| 1587 |  | HLA-DPA1*0301-DPB1*1701 | HLA-DQA1*0404-DQB1*0321 |
| 1588 |  | HLA-DPA1*0301-DPB1*1801 | HLA-DQA1*0404-DQB1*0322 |
| 1589 |  | HLA-DPA1*0301-DPB1*1901 | HLA-DQA1*0404-DQB1*0323 |
| 1590 |  | HLA-DPA1*0301-DPB1*2001 | HLA-DQA1*0404-DQB1*0324 |
| 1591 |  | HLA-DPA1*0301-DPB1*2101 | HLA-DQA1*0404-DQB1*0325 |
| 1592 |  | HLA-DPA1*0301-DPB1*2201 | HLA-DQA1*0404-DQB1*0326 |
| 1593 |  | HLA-DPA1*0301-DPB1*2301 | HLA-DQA1*0404-DQB1*0327 |
| 1594 |  | HLA-DPA1*0301-DPB1*2401 | HLA-DQA1*0404-DQB1*0328 |
| 1595 |  | HLA-DPA1*0301-DPB1*2501 | HLA-DQA1*0404-DQB1*0329 |
| 1596 |  | HLA-DPA1*0301-DPB1*2601 | HLA-DQA1*0404-DQB1*0330 |
| 1597 |  | HLA-DPA1*0301-DPB1*2701 | HLA-DQA1*0404-DQB1*0331 |
| 1598 |  | HLA-DPA1*0301-DPB1*2801 | HLA-DQA1*0404-DQB1*0332 |
| 1599 |  | HLA-DPA1*0301-DPB1*2901 | HLA-DQA1*0404-DQB1*0333 |
| 1600 |  | HLA-DPA1*0301-DPB1*3001 | HLA-DQA1*0404-DQB1*0334 |
| 1601 |  | HLA-DPA1*0301-DPB1*3101 | HLA-DQA1*0404-DQB1*0335 |
| 1602 |  | HLA-DPA1*0301-DPB1*3201 | HLA-DQA1*0404-DQB1*0336 |
| 1603 |  | HLA-DPA1*0301-DPB1*3301 | HLA-DQA1*0404-DQB1*0337 |
| 1604 |  | HLA-DPA1*0301-DPB1*3401 | HLA-DQA1*0404-DQB1*0338 |
| 1605 |  | HLA-DPA1*0301-DPB1*3501 | HLA-DQA1*0404-DQB1*0401 |
| 1606 |  | HLA-DPA1*0301-DPB1*3601 | HLA-DQA1*0404-DQB1*0402 |
| 1607 |  | HLA-DPA1*0301-DPB1*3701 | HLA-DQA1*0404-DQB1*0403 |
| 1608 |  | HLA-DPA1*0301-DPB1*3801 | HLA-DQA1*0404-DQB1*0404 |
| 1609 |  | HLA-DPA1*0301-DPB1*3901 | HLA-DQA1*0404-DQB1*0405 |
| 1610 |  | HLA-DPA1*0301-DPB1*4001 | HLA-DQA1*0404-DQB1*0406 |
| 1611 |  | HLA-DPA1*0301-DPB1*4101 | HLA-DQA1*0404-DQB1*0407 |
| 1612 |  | HLA-DPA1*0301-DPB1*4401 | HLA-DQA1*0404-DQB1*0408 |
| 1613 |  | HLA-DPA1*0301-DPB1*4501 | HLA-DQA1*0404-DQB1*0501 |
| 1614 |  | HLA-DPA1*0301-DPB1*4601 | HLA-DQA1*0404-DQB1*0502 |
| 1615 |  | HLA-DPA1*0301-DPB1*4701 | HLA-DQA1*0404-DQB1*0503 |
| 1616 |  | HLA-DPA1*0301-DPB1*4801 | HLA-DQA1*0404-DQB1*0505 |
| 1617 |  | HLA-DPA1*0301-DPB1*4901 | HLA-DQA1*0404-DQB1*0506 |
| 1618 |  | HLA-DPA1*0301-DPB1*5001 | HLA-DQA1*0404-DQB1*0507 |
| 1619 |  | HLA-DPA1*0301-DPB1*5101 | HLA-DQA1*0404-DQB1*0508 |
| 1620 |  | HLA-DPA1*0301-DPB1*5201 | HLA-DQA1*0404-DQB1*0509 |
| 1621 |  | HLA-DPA1*0301-DPB1*5301 | HLA-DQA1*0404-DQB1*0510 |
| 1622 |  | HLA-DPA1*0301-DPB1*5401 | HLA-DQA1*0404-DQB1*0511 |
| 1623 |  | HLA-DPA1*0301-DPB1*5501 | HLA-DQA1*0404-DQB1*0512 |
| 1624 |  | HLA-DPA1*0301-DPB1*5601 | HLA-DQA1*0404-DQB1*0513 |
| 1625 |  | HLA-DPA1*0301-DPB1*5801 | HLA-DQA1*0404-DQB1*0514 |
| 1626 |  | HLA-DPA1*0301-DPB1*5901 | HLA-DQA1*0404-DQB1*0601 |
| 1627 |  | HLA-DPA1*0301-DPB1*6001 | HLA-DQA1*0404-DQB1*0602 |
| 1628 |  | HLA-DPA1*0301-DPB1*6201 | HLA-DQA1*0404-DQB1*0603 |
| 1629 |  | HLA-DPA1*0301-DPB1*6301 | HLA-DQA1*0404-DQB1*0604 |
| 1630 |  | HLA-DPA1*0301-DPB1*6501 | HLA-DQA1*0404-DQB1*0607 |
| 1631 |  | HLA-DPA1*0301-DPB1*6601 | HLA-DQA1*0404-DQB1*0608 |
| 1632 |  | HLA-DPA1*0301-DPB1*6701 | HLA-DQA1*0404-DQB1*0609 |
| 1633 |  | HLA-DPA1*0301-DPB1*6801 | HLA-DQA1*0404-DQB1*0610 |
| 1634 |  | HLA-DPA1*0301-DPB1*6901 | HLA-DQA1*0404-DQB1*0611 |
| 1635 |  | HLA-DPA1*0301-DPB1*7001 | HLA-DQA1*0404-DQB1*0612 |
| 1636 |  | HLA-DPA1*0301-DPB1*7101 | HLA-DQA1*0404-DQB1*0614 |
| 1637 |  | HLA-DPA1*0301-DPB1*7201 | HLA-DQA1*0404-DQB1*0615 |
| 1638 |  | HLA-DPA1*0301-DPB1*7301 | HLA-DQA1*0404-DQB1*0616 |
| 1639 |  | HLA-DPA1*0301-DPB1*7401 | HLA-DQA1*0404-DQB1*0617 |
| 1640 |  | HLA-DPA1*0301-DPB1*7501 | HLA-DQA1*0404-DQB1*0618 |
| 1641 |  | HLA-DPA1*0301-DPB1*7601 | HLA-DQA1*0404-DQB1*0619 |
| 1642 |  | HLA-DPA1*0301-DPB1*7701 | HLA-DQA1*0404-DQB1*0621 |
| 1643 |  | HLA-DPA1*0301-DPB1*7801 | HLA-DQA1*0404-DQB1*0622 |
| 1644 |  | HLA-DPA1*0301-DPB1*7901 | HLA-DQA1*0404-DQB1*0623 |
| 1645 |  | HLA-DPA1*0301-DPB1*8001 | HLA-DQA1*0404-DQB1*0624 |
| 1646 |  | HLA-DPA1*0301-DPB1*8101 | HLA-DQA1*0404-DQB1*0625 |
| 1647 |  | HLA-DPA1*0301-DPB1*8201 | HLA-DQA1*0404-DQB1*0627 |
| 1648 |  | HLA-DPA1*0301-DPB1*8301 | HLA-DQA1*0404-DQB1*0628 |
| 1649 |  | HLA-DPA1*0301-DPB1*8401 | HLA-DQA1*0404-DQB1*0629 |
| 1650 |  | HLA-DPA1*0301-DPB1*8501 | HLA-DQA1*0404-DQB1*0630 |
| 1651 |  | HLA-DPA1*0301-DPB1*8601 | HLA-DQA1*0404-DQB1*0631 |
| 1652 |  | HLA-DPA1*0301-DPB1*8701 | HLA-DQA1*0404-DQB1*0632 |
| 1653 |  | HLA-DPA1*0301-DPB1*8801 | HLA-DQA1*0404-DQB1*0633 |
| 1654 |  | HLA-DPA1*0301-DPB1*8901 | HLA-DQA1*0404-DQB1*0634 |
| 1655 |  | HLA-DPA1*0301-DPB1*9001 | HLA-DQA1*0404-DQB1*0635 |
| 1656 |  | HLA-DPA1*0301-DPB1*9101 | HLA-DQA1*0404-DQB1*0636 |
| 1657 |  | HLA-DPA1*0301-DPB1*9201 | HLA-DQA1*0404-DQB1*0637 |
| 1658 |  | HLA-DPA1*0301-DPB1*9301 | HLA-DQA1*0404-DQB1*0638 |
| 1659 |  | HLA-DPA1*0301-DPB1*9401 | HLA-DQA1*0404-DQB1*0639 |
| 1660 |  | HLA-DPA1*0301-DPB1*9501 | HLA-DQA1*0404-DQB1*0640 |
| 1661 |  | HLA-DPA1*0301-DPB1*9601 | HLA-DQA1*0404-DQB1*0641 |
| 1662 |  | HLA-DPA1*0301-DPB1*9701 | HLA-DQA1*0404-DQB1*0642 |
| 1663 |  | HLA-DPA1*0301-DPB1*9801 | HLA-DQA1*0404-DQB1*0643 |
| 1664 |  | HLA-DPA1*0301-DPB1*9901 | HLA-DQA1*0404-DQB1*0644 |
| 1665 |  | HLA-DPA1*0302-DPB1*0101 | HLA-DQA1*0501-DQB1*0201 |
| 1666 |  | HLA-DPA1*0302-DPB1*0201 | HLA-DQA1*0501-DQB1*0202 |
| 1667 |  | HLA-DPA1*0302-DPB1*0202 | HLA-DQA1*0501-DQB1*0203 |
| 1668 |  | HLA-DPA1*0302-DPB1*0301 | HLA-DQA1*0501-DQB1*0204 |
| 1669 |  | HLA-DPA1*0302-DPB1*0401 | HLA-DQA1*0501-DQB1*0205 |
| 1670 |  | HLA-DPA1*0302-DPB1*0402 | HLA-DQA1*0501-DQB1*0206 |
| 1671 |  | HLA-DPA1*0302-DPB1*0501 | HLA-DQA1*0501-DQB1*0301 |
| 1672 |  | HLA-DPA1*0302-DPB1*0601 | HLA-DQA1*0501-DQB1*0302 |
| 1673 |  | HLA-DPA1*0302-DPB1*0801 | HLA-DQA1*0501-DQB1*0303 |
| 1674 |  | HLA-DPA1*0302-DPB1*0901 | HLA-DQA1*0501-DQB1*0304 |
| 1675 |  | HLA-DPA1*0302-DPB1*0001 | HLA-DQA1*0501-DQB1*0305 |
| 1676 |  | HLA-DPA1*0302-DPB1*1001 | HLA-DQA1*0501-DQB1*0306 |
| 1677 |  | HLA-DPA1*0302-DPB1*0101 | HLA-DQA1*0501-DQB1*0307 |
| 1678 |  | HLA-DPA1*0302-DPB1*0201 | HLA-DQA1*0501-DQB1*0308 |
| 1679 |  | HLA-DPA1*0302-DPB1*0301 | HLA-DQA1*0501-DQB1*0309 |
| 1680 |  | HLA-DPA1*0302-DPB1*0401 | HLA-DQA1*0501-DQB1*0310 |
| 1681 |  | HLA-DPA1*0302-DPB1*0501 | HLA-DQA1*0501-DQB1*0311 |
| 1682 |  | HLA-DPA1*0302-DPB1*0601 | HLA-DQA1*0501-DQB1*0312 |
| 1683 |  | HLA-DPA1*0302-DPB1*0701 | HLA-DQA1*0501-DQB1*0313 |
| 1684 |  | HLA-DPA1*0302-DPB1*0801 | HLA-DQA1*0501-DQB1*0314 |
| 1685 |  | HLA-DPA1*0302-DPB1*0901 | HLA-DQA1*0501-DQB1*0315 |
| 1686 |  | HLA-DPA1*0302-DPB1*1001 | HLA-DQA1*0501-DQB1*0316 |
| 1687 |  | HLA-DPA1*0302-DPB1*1101 | HLA-DQA1*0501-DQB1*0317 |
| 1688 |  | HLA-DPA1*0302-DPB1*1101 | HLA-DQA1*0501-DQB1*0318 |
| 1689 |  | HLA-DPA1*0302-DPB1*1201 | HLA-DQA1*0501-DQB1*0319 |
| 1690 |  | HLA-DPA1*0302-DPB1*1301 | HLA-DQA1*0501-DQB1*0320 |
| 1691 |  | HLA-DPA1*0302-DPB1*1401 | HLA-DQA1*0501-DQB1*0321 |
| 1692 |  | HLA-DPA1*0302-DPB1*1501 | HLA-DQA1*0501-DQB1*0322 |
| 1693 |  | HLA-DPA1*0302-DPB1*1601 | HLA-DQA1*0501-DQB1*0323 |
| 1694 |  | HLA-DPA1*0302-DPB1*1701 | HLA-DQA1*0501-DQB1*0324 |
| 1695 |  | HLA-DPA1*0302-DPB1*1801 | HLA-DQA1*0501-DQB1*0325 |
| 1696 |  | HLA-DPA1*0302-DPB1*1901 | HLA-DQA1*0501-DQB1*0326 |
| 1697 |  | HLA-DPA1*0302-DPB1*2101 | HLA-DQA1*0501-DQB1*0327 |
| 1698 |  | HLA-DPA1*0302-DPB1*2201 | HLA-DQA1*0501-DQB1*0328 |
| 1699 |  | HLA-DPA1*0302-DPB1*2301 | HLA-DQA1*0501-DQB1*0329 |
| 1700 |  | HLA-DPA1*0302-DPB1*2401 | HLA-DQA1*0501-DQB1*0330 |
| 1701 |  | HLA-DPA1*0302-DPB1*2501 | HLA-DQA1*0501-DQB1*0331 |
| 1702 |  | HLA-DPA1*0302-DPB1*2601 | HLA-DQA1*0501-DQB1*0332 |
| 1703 |  | HLA-DPA1*0302-DPB1*2701 | HLA-DQA1*0501-DQB1*0333 |
| 1704 |  | HLA-DPA1*0302-DPB1*2801 | HLA-DQA1*0501-DQB1*0334 |
| 1705 |  | HLA-DPA1*0302-DPB1*2901 | HLA-DQA1*0501-DQB1*0335 |
| 1706 |  | HLA-DPA1*0302-DPB1*3001 | HLA-DQA1*0501-DQB1*0336 |
| 1707 |  | HLA-DPA1*0302-DPB1*1301 | HLA-DQA1*0501-DQB1*0337 |
| 1708 |  | HLA-DPA1*0302-DPB1*3101 | HLA-DQA1*0501-DQB1*0338 |
| 1709 |  | HLA-DPA1*0302-DPB1*3201 | HLA-DQA1*0501-DQB1*0401 |
| 1710 |  | HLA-DPA1*0302-DPB1*3301 | HLA-DQA1*0501-DQB1*0402 |
| 1711 |  | HLA-DPA1*0302-DPB1*3401 | HLA-DQA1*0501-DQB1*0403 |
| 1712 |  | HLA-DPA1*0302-DPB1*1401 | HLA-DQA1*0501-DQB1*0404 |
| 1713 |  | HLA-DPA1*0302-DPB1*1501 | HLA-DQA1*0501-DQB1*0405 |
| 1714 |  | HLA-DPA1*0302-DPB1*1601 | HLA-DQA1*0501-DQB1*0406 |
| 1715 |  | HLA-DPA1*0302-DPB1*1701 | HLA-DQA1*0501-DQB1*0407 |
| 1716 |  | HLA-DPA1*0302-DPB1*1801 | HLA-DQA1*0501-DQB1*0408 |
| 1717 |  | HLA-DPA1*0302-DPB1*1901 | HLA-DQA1*0501-DQB1*0501 |
| 1718 |  | HLA-DPA1*0302-DPB1*2001 | HLA-DQA1*0501-DQB1*0502 |
| 1719 |  | HLA-DPA1*0302-DPB1*2101 | HLA-DQA1*0501-DQB1*0503 |
| 1720 |  | HLA-DPA1*0302-DPB1*2201 | HLA-DQA1*0501-DQB1*0505 |
| 1721 |  | HLA-DPA1*0302-DPB1*2301 | HLA-DQA1*0501-DQB1*0506 |
| 1722 |  | HLA-DPA1*0302-DPB1*2401 | HLA-DQA1*0501-DQB1*0507 |
| 1723 |  | HLA-DPA1*0302-DPB1*2501 | HLA-DQA1*0501-DQB1*0508 |
| 1724 |  | HLA-DPA1*0302-DPB1*2601 | HLA-DQA1*0501-DQB1*0509 |
| 1725 |  | HLA-DPA1*0302-DPB1*2701 | HLA-DQA1*0501-DQB1*0510 |
| 1726 |  | HLA-DPA1*0302-DPB1*2801 | HLA-DQA1*0501-DQB1*0511 |
| 1727 |  | HLA-DPA1*0302-DPB1*2901 | HLA-DQA1*0501-DQB1*0512 |
| 1728 |  | HLA-DPA1*0302-DPB1*3001 | HLA-DQA1*0501-DQB1*0513 |
| 1729 |  | HLA-DPA1*0302-DPB1*3101 | HLA-DQA1*0501-DQB1*0514 |
| 1730 |  | HLA-DPA1*0302-DPB1*3201 | HLA-DQA1*0501-DQB1*0601 |
| 1731 |  | HLA-DPA1*0302-DPB1*3301 | HLA-DQA1*0501-DQB1*0602 |
| 1732 |  | HLA-DPA1*0302-DPB1*3401 | HLA-DQA1*0501-DQB1*0603 |
| 1733 |  | HLA-DPA1*0302-DPB1*3501 | HLA-DQA1*0501-DQB1*0604 |
| 1734 |  | HLA-DPA1*0302-DPB1*3601 | HLA-DQA1*0501-DQB1*0607 |
| 1735 |  | HLA-DPA1*0302-DPB1*3701 | HLA-DQA1*0501-DQB1*0608 |
| 1736 |  | HLA-DPA1*0302-DPB1*3801 | HLA-DQA1*0501-DQB1*0609 |
| 1737 |  | HLA-DPA1*0302-DPB1*3901 | HLA-DQA1*0501-DQB1*0610 |
| 1738 |  | HLA-DPA1*0302-DPB1*4001 | HLA-DQA1*0501-DQB1*0611 |
| 1739 |  | HLA-DPA1*0302-DPB1*4101 | HLA-DQA1*0501-DQB1*0612 |
| 1740 |  | HLA-DPA1*0302-DPB1*4401 | HLA-DQA1*0501-DQB1*0614 |
| 1741 |  | HLA-DPA1*0302-DPB1*4501 | HLA-DQA1*0501-DQB1*0615 |
| 1742 |  | HLA-DPA1*0302-DPB1*4601 | HLA-DQA1*0501-DQB1*0616 |
| 1743 |  | HLA-DPA1*0302-DPB1*4701 | HLA-DQA1*0501-DQB1*0617 |
| 1744 |  | HLA-DPA1*0302-DPB1*4801 | HLA-DQA1*0501-DQB1*0618 |
| 1745 |  | HLA-DPA1*0302-DPB1*4901 | HLA-DQA1*0501-DQB1*0619 |
| 1746 |  | HLA-DPA1*0302-DPB1*5001 | HLA-DQA1*0501-DQB1*0621 |
| 1747 |  | HLA-DPA1*0302-DPB1*5101 | HLA-DQA1*0501-DQB1*0622 |
| 1748 |  | HLA-DPA1*0302-DPB1*5201 | HLA-DQA1*0501-DQB1*0623 |
| 1749 |  | HLA-DPA1*0302-DPB1*5301 | HLA-DQA1*0501-DQB1*0624 |
| 1750 |  | HLA-DPA1*0302-DPB1*5401 | HLA-DQA1*0501-DQB1*0625 |
| 1751 |  | HLA-DPA1*0302-DPB1*5501 | HLA-DQA1*0501-DQB1*0627 |
| 1752 |  | HLA-DPA1*0302-DPB1*5601 | HLA-DQA1*0501-DQB1*0628 |
| 1753 |  | HLA-DPA1*0302-DPB1*5801 | HLA-DQA1*0501-DQB1*0629 |
| 1754 |  | HLA-DPA1*0302-DPB1*5901 | HLA-DQA1*0501-DQB1*0630 |
| 1755 |  | HLA-DPA1*0302-DPB1*6001 | HLA-DQA1*0501-DQB1*0631 |
| 1756 |  | HLA-DPA1*0302-DPB1*6201 | HLA-DQA1*0501-DQB1*0632 |
| 1757 |  | HLA-DPA1*0302-DPB1*6301 | HLA-DQA1*0501-DQB1*0633 |
| 1758 |  | HLA-DPA1*0302-DPB1*6501 | HLA-DQA1*0501-DQB1*0634 |
| 1759 |  | HLA-DPA1*0302-DPB1*6601 | HLA-DQA1*0501-DQB1*0635 |
| 1760 |  | HLA-DPA1*0302-DPB1*6701 | HLA-DQA1*0501-DQB1*0636 |
| 1761 |  | HLA-DPA1*0302-DPB1*6801 | HLA-DQA1*0501-DQB1*0637 |
| 1762 |  | HLA-DPA1*0302-DPB1*6901 | HLA-DQA1*0501-DQB1*0638 |
| 1763 |  | HLA-DPA1*0302-DPB1*7001 | HLA-DQA1*0501-DQB1*0639 |
| 1764 |  | HLA-DPA1*0302-DPB1*7101 | HLA-DQA1*0501-DQB1*0640 |
| 1765 |  | HLA-DPA1*0302-DPB1*7201 | HLA-DQA1*0501-DQB1*0641 |
| 1766 |  | HLA-DPA1*0302-DPB1*7301 | HLA-DQA1*0501-DQB1*0642 |
| 1767 |  | HLA-DPA1*0302-DPB1*7401 | HLA-DQA1*0501-DQB1*0643 |
| 1768 |  | HLA-DPA1*0302-DPB1*7501 | HLA-DQA1*0501-DQB1*0644 |
| 1769 |  | HLA-DPA1*0302-DPB1*7601 | HLA-DQA1*0503-DQB1*0201 |
| 1770 |  | HLA-DPA1*0302-DPB1*7701 | HLA-DQA1*0503-DQB1*0202 |
| 1771 |  | HLA-DPA1*0302-DPB1*7801 | HLA-DQA1*0503-DQB1*0203 |
| 1772 |  | HLA-DPA1*0302-DPB1*7901 | HLA-DQA1*0503-DQB1*0204 |
| 1773 |  | HLA-DPA1*0302-DPB1*8001 | HLA-DQA1*0503-DQB1*0205 |
| 1774 |  | HLA-DPA1*0302-DPB1*8101 | HLA-DQA1*0503-DQB1*0206 |
| 1775 |  | HLA-DPA1*0302-DPB1*8201 | HLA-DQA1*0503-DQB1*0301 |
| 1776 |  | HLA-DPA1*0302-DPB1*8301 | HLA-DQA1*0503-DQB1*0302 |
| 1777 |  | HLA-DPA1*0302-DPB1*8401 | HLA-DQA1*0503-DQB1*0303 |
| 1778 |  | HLA-DPA1*0302-DPB1*8501 | HLA-DQA1*0503-DQB1*0304 |
| 1779 |  | HLA-DPA1*0302-DPB1*8601 | HLA-DQA1*0503-DQB1*0305 |
| 1780 |  | HLA-DPA1*0302-DPB1*8701 | HLA-DQA1*0503-DQB1*0306 |
| 1781 |  | HLA-DPA1*0302-DPB1*8801 | HLA-DQA1*0503-DQB1*0307 |
| 1782 |  | HLA-DPA1*0302-DPB1*8901 | HLA-DQA1*0503-DQB1*0308 |
| 1783 |  | HLA-DPA1*0302-DPB1*9001 | HLA-DQA1*0503-DQB1*0309 |
| 1784 |  | HLA-DPA1*0302-DPB1*9101 | HLA-DQA1*0503-DQB1*0310 |
| 1785 |  | HLA-DPA1*0302-DPB1*9201 | HLA-DQA1*0503-DQB1*0311 |
| 1786 |  | HLA-DPA1*0302-DPB1*9301 | HLA-DQA1*0503-DQB1*0312 |
| 1787 |  | HLA-DPA1*0302-DPB1*9401 | HLA-DQA1*0503-DQB1*0313 |
| 1788 |  | HLA-DPA1*0302-DPB1*9501 | HLA-DQA1*0503-DQB1*0314 |
| 1789 |  | HLA-DPA1*0302-DPB1*9601 | HLA-DQA1*0503-DQB1*0315 |
| 1790 |  | HLA-DPA1*0302-DPB1*9701 | HLA-DQA1*0503-DQB1*0316 |
| 1791 |  | HLA-DPA1*0302-DPB1*9801 | HLA-DQA1*0503-DQB1*0317 |
| 1792 |  | HLA-DPA1*0302-DPB1*9901 | HLA-DQA1*0503-DQB1*0318 |
| 1793 |  | HLA-DPA1*0303-DPB1*0101 | HLA-DQA1*0503-DQB1*0319 |
| 1794 |  | HLA-DPA1*0303-DPB1*0201 | HLA-DQA1*0503-DQB1*0320 |
| 1795 |  | HLA-DPA1*0303-DPB1*0202 | HLA-DQA1*0503-DQB1*0321 |
| 1796 |  | HLA-DPA1*0303-DPB1*0301 | HLA-DQA1*0503-DQB1*0322 |
| 1797 |  | HLA-DPA1*0303-DPB1*0401 | HLA-DQA1*0503-DQB1*0323 |
| 1798 |  | HLA-DPA1*0303-DPB1*0402 | HLA-DQA1*0503-DQB1*0324 |
| 1799 |  | HLA-DPA1*0303-DPB1*0501 | HLA-DQA1*0503-DQB1*0325 |
| 1800 |  | HLA-DPA1*0303-DPB1*0601 | HLA-DQA1*0503-DQB1*0326 |
| 1801 |  | HLA-DPA1*0303-DPB1*0801 | HLA-DQA1*0503-DQB1*0327 |
| 1802 |  | HLA-DPA1*0303-DPB1*0901 | HLA-DQA1*0503-DQB1*0328 |
| 1803 |  | HLA-DPA1*0303-DPB1*0001 | HLA-DQA1*0503-DQB1*0329 |
| 1804 |  | HLA-DPA1*0303-DPB1*1001 | HLA-DQA1*0503-DQB1*0330 |
| 1805 |  | HLA-DPA1*0303-DPB1*0101 | HLA-DQA1*0503-DQB1*0331 |
| 1806 |  | HLA-DPA1*0303-DPB1*0201 | HLA-DQA1*0503-DQB1*0332 |
| 1807 |  | HLA-DPA1*0303-DPB1*0301 | HLA-DQA1*0503-DQB1*0333 |
| 1808 |  | HLA-DPA1*0303-DPB1*0401 | HLA-DQA1*0503-DQB1*0334 |
| 1809 |  | HLA-DPA1*0303-DPB1*0501 | HLA-DQA1*0503-DQB1*0335 |
| 1810 |  | HLA-DPA1*0303-DPB1*0601 | HLA-DQA1*0503-DQB1*0336 |
| 1811 |  | HLA-DPA1*0303-DPB1*0701 | HLA-DQA1*0503-DQB1*0337 |
| 1812 |  | HLA-DPA1*0303-DPB1*0801 | HLA-DQA1*0503-DQB1*0338 |
| 1813 |  | HLA-DPA1*0303-DPB1*0901 | HLA-DQA1*0503-DQB1*0401 |
| 1814 |  | HLA-DPA1*0303-DPB1*1001 | HLA-DQA1*0503-DQB1*0402 |
| 1815 |  | HLA-DPA1*0303-DPB1*1101 | HLA-DQA1*0503-DQB1*0403 |
| 1816 |  | HLA-DPA1*0303-DPB1*1101 | HLA-DQA1*0503-DQB1*0404 |
| 1817 |  | HLA-DPA1*0303-DPB1*1201 | HLA-DQA1*0503-DQB1*0405 |
| 1818 |  | HLA-DPA1*0303-DPB1*1301 | HLA-DQA1*0503-DQB1*0406 |
| 1819 |  | HLA-DPA1*0303-DPB1*1401 | HLA-DQA1*0503-DQB1*0407 |
| 1820 |  | HLA-DPA1*0303-DPB1*1501 | HLA-DQA1*0503-DQB1*0408 |
| 1821 |  | HLA-DPA1*0303-DPB1*1601 | HLA-DQA1*0503-DQB1*0501 |
| 1822 |  | HLA-DPA1*0303-DPB1*1701 | HLA-DQA1*0503-DQB1*0502 |
| 1823 |  | HLA-DPA1*0303-DPB1*1801 | HLA-DQA1*0503-DQB1*0503 |
| 1824 |  | HLA-DPA1*0303-DPB1*1901 | HLA-DQA1*0503-DQB1*0505 |
| 1825 |  | HLA-DPA1*0303-DPB1*2101 | HLA-DQA1*0503-DQB1*0506 |
| 1826 |  | HLA-DPA1*0303-DPB1*2201 | HLA-DQA1*0503-DQB1*0507 |
| 1827 |  | HLA-DPA1*0303-DPB1*2301 | HLA-DQA1*0503-DQB1*0508 |
| 1828 |  | HLA-DPA1*0303-DPB1*2401 | HLA-DQA1*0503-DQB1*0509 |
| 1829 |  | HLA-DPA1*0303-DPB1*2501 | HLA-DQA1*0503-DQB1*0510 |
| 1830 |  | HLA-DPA1*0303-DPB1*2601 | HLA-DQA1*0503-DQB1*0511 |
| 1831 |  | HLA-DPA1*0303-DPB1*2701 | HLA-DQA1*0503-DQB1*0512 |
| 1832 |  | HLA-DPA1*0303-DPB1*2801 | HLA-DQA1*0503-DQB1*0513 |
| 1833 |  | HLA-DPA1*0303-DPB1*2901 | HLA-DQA1*0503-DQB1*0514 |
| 1834 |  | HLA-DPA1*0303-DPB1*3001 | HLA-DQA1*0503-DQB1*0601 |
| 1835 |  | HLA-DPA1*0303-DPB1*1301 | HLA-DQA1*0503-DQB1*0602 |
| 1836 |  | HLA-DPA1*0303-DPB1*3101 | HLA-DQA1*0503-DQB1*0603 |
| 1837 |  | HLA-DPA1*0303-DPB1*3201 | HLA-DQA1*0503-DQB1*0604 |
| 1838 |  | HLA-DPA1*0303-DPB1*3301 | HLA-DQA1*0503-DQB1*0607 |
| 1839 |  | HLA-DPA1*0303-DPB1*3401 | HLA-DQA1*0503-DQB1*0608 |
| 1840 |  | HLA-DPA1*0303-DPB1*1401 | HLA-DQA1*0503-DQB1*0609 |
| 1841 |  | HLA-DPA1*0303-DPB1*1501 | HLA-DQA1*0503-DQB1*0610 |
| 1842 |  | HLA-DPA1*0303-DPB1*1601 | HLA-DQA1*0503-DQB1*0611 |
| 1843 |  | HLA-DPA1*0303-DPB1*1701 | HLA-DQA1*0503-DQB1*0612 |
| 1844 |  | HLA-DPA1*0303-DPB1*1801 | HLA-DQA1*0503-DQB1*0614 |
| 1845 |  | HLA-DPA1*0303-DPB1*1901 | HLA-DQA1*0503-DQB1*0615 |
| 1846 |  | HLA-DPA1*0303-DPB1*2001 | HLA-DQA1*0503-DQB1*0616 |
| 1847 |  | HLA-DPA1*0303-DPB1*2101 | HLA-DQA1*0503-DQB1*0617 |
| 1848 |  | HLA-DPA1*0303-DPB1*2201 | HLA-DQA1*0503-DQB1*0618 |
| 1849 |  | HLA-DPA1*0303-DPB1*2301 | HLA-DQA1*0503-DQB1*0619 |
| 1850 |  | HLA-DPA1*0303-DPB1*2401 | HLA-DQA1*0503-DQB1*0621 |
| 1851 |  | HLA-DPA1*0303-DPB1*2501 | HLA-DQA1*0503-DQB1*0622 |
| 1852 |  | HLA-DPA1*0303-DPB1*2601 | HLA-DQA1*0503-DQB1*0623 |
| 1853 |  | HLA-DPA1*0303-DPB1*2701 | HLA-DQA1*0503-DQB1*0624 |
| 1854 |  | HLA-DPA1*0303-DPB1*2801 | HLA-DQA1*0503-DQB1*0625 |
| 1855 |  | HLA-DPA1*0303-DPB1*2901 | HLA-DQA1*0503-DQB1*0627 |
| 1856 |  | HLA-DPA1*0303-DPB1*3001 | HLA-DQA1*0503-DQB1*0628 |
| 1857 |  | HLA-DPA1*0303-DPB1*3101 | HLA-DQA1*0503-DQB1*0629 |
| 1858 |  | HLA-DPA1*0303-DPB1*3201 | HLA-DQA1*0503-DQB1*0630 |
| 1859 |  | HLA-DPA1*0303-DPB1*3301 | HLA-DQA1*0503-DQB1*0631 |
| 1860 |  | HLA-DPA1*0303-DPB1*3401 | HLA-DQA1*0503-DQB1*0632 |
| 1861 |  | HLA-DPA1*0303-DPB1*3501 | HLA-DQA1*0503-DQB1*0633 |
| 1862 |  | HLA-DPA1*0303-DPB1*3601 | HLA-DQA1*0503-DQB1*0634 |
| 1863 |  | HLA-DPA1*0303-DPB1*3701 | HLA-DQA1*0503-DQB1*0635 |
| 1864 |  | HLA-DPA1*0303-DPB1*3801 | HLA-DQA1*0503-DQB1*0636 |
| 1865 |  | HLA-DPA1*0303-DPB1*3901 | HLA-DQA1*0503-DQB1*0637 |
| 1866 |  | HLA-DPA1*0303-DPB1*4001 | HLA-DQA1*0503-DQB1*0638 |
| 1867 |  | HLA-DPA1*0303-DPB1*4101 | HLA-DQA1*0503-DQB1*0639 |
| 1868 |  | HLA-DPA1*0303-DPB1*4401 | HLA-DQA1*0503-DQB1*0640 |
| 1869 |  | HLA-DPA1*0303-DPB1*4501 | HLA-DQA1*0503-DQB1*0641 |
| 1870 |  | HLA-DPA1*0303-DPB1*4601 | HLA-DQA1*0503-DQB1*0642 |
| 1871 |  | HLA-DPA1*0303-DPB1*4701 | HLA-DQA1*0503-DQB1*0643 |
| 1872 |  | HLA-DPA1*0303-DPB1*4801 | HLA-DQA1*0503-DQB1*0644 |
| 1873 |  | HLA-DPA1*0303-DPB1*4901 | HLA-DQA1*0504-DQB1*0201 |
| 1874 |  | HLA-DPA1*0303-DPB1*5001 | HLA-DQA1*0504-DQB1*0202 |
| 1875 |  | HLA-DPA1*0303-DPB1*5101 | HLA-DQA1*0504-DQB1*0203 |
| 1876 |  | HLA-DPA1*0303-DPB1*5201 | HLA-DQA1*0504-DQB1*0204 |
| 1877 |  | HLA-DPA1*0303-DPB1*5301 | HLA-DQA1*0504-DQB1*0205 |
| 1878 |  | HLA-DPA1*0303-DPB1*5401 | HLA-DQA1*0504-DQB1*0206 |
| 1879 |  | HLA-DPA1*0303-DPB1*5501 | HLA-DQA1*0504-DQB1*0301 |
| 1880 |  | HLA-DPA1*0303-DPB1*5601 | HLA-DQA1*0504-DQB1*0302 |
| 1881 |  | HLA-DPA1*0303-DPB1*5801 | HLA-DQA1*0504-DQB1*0303 |
| 1882 |  | HLA-DPA1*0303-DPB1*5901 | HLA-DQA1*0504-DQB1*0304 |
| 1883 |  | HLA-DPA1*0303-DPB1*6001 | HLA-DQA1*0504-DQB1*0305 |
| 1884 |  | HLA-DPA1*0303-DPB1*6201 | HLA-DQA1*0504-DQB1*0306 |
| 1885 |  | HLA-DPA1*0303-DPB1*6301 | HLA-DQA1*0504-DQB1*0307 |
| 1886 |  | HLA-DPA1*0303-DPB1*6501 | HLA-DQA1*0504-DQB1*0308 |
| 1887 |  | HLA-DPA1*0303-DPB1*6601 | HLA-DQA1*0504-DQB1*0309 |
| 1888 |  | HLA-DPA1*0303-DPB1*6701 | HLA-DQA1*0504-DQB1*0310 |
| 1889 |  | HLA-DPA1*0303-DPB1*6801 | HLA-DQA1*0504-DQB1*0311 |
| 1890 |  | HLA-DPA1*0303-DPB1*6901 | HLA-DQA1*0504-DQB1*0312 |
| 1891 |  | HLA-DPA1*0303-DPB1*7001 | HLA-DQA1*0504-DQB1*0313 |
| 1892 |  | HLA-DPA1*0303-DPB1*7101 | HLA-DQA1*0504-DQB1*0314 |
| 1893 |  | HLA-DPA1*0303-DPB1*7201 | HLA-DQA1*0504-DQB1*0315 |
| 1894 |  | HLA-DPA1*0303-DPB1*7301 | HLA-DQA1*0504-DQB1*0316 |
| 1895 |  | HLA-DPA1*0303-DPB1*7401 | HLA-DQA1*0504-DQB1*0317 |
| 1896 |  | HLA-DPA1*0303-DPB1*7501 | HLA-DQA1*0504-DQB1*0318 |
| 1897 |  | HLA-DPA1*0303-DPB1*7601 | HLA-DQA1*0504-DQB1*0319 |
| 1898 |  | HLA-DPA1*0303-DPB1*7701 | HLA-DQA1*0504-DQB1*0320 |
| 1899 |  | HLA-DPA1*0303-DPB1*7801 | HLA-DQA1*0504-DQB1*0321 |
| 1900 |  | HLA-DPA1*0303-DPB1*7901 | HLA-DQA1*0504-DQB1*0322 |
| 1901 |  | HLA-DPA1*0303-DPB1*8001 | HLA-DQA1*0504-DQB1*0323 |
| 1902 |  | HLA-DPA1*0303-DPB1*8101 | HLA-DQA1*0504-DQB1*0324 |
| 1903 |  | HLA-DPA1*0303-DPB1*8201 | HLA-DQA1*0504-DQB1*0325 |
| 1904 |  | HLA-DPA1*0303-DPB1*8301 | HLA-DQA1*0504-DQB1*0326 |
| 1905 |  | HLA-DPA1*0303-DPB1*8401 | HLA-DQA1*0504-DQB1*0327 |
| 1906 |  | HLA-DPA1*0303-DPB1*8501 | HLA-DQA1*0504-DQB1*0328 |
| 1907 |  | HLA-DPA1*0303-DPB1*8601 | HLA-DQA1*0504-DQB1*0329 |
| 1908 |  | HLA-DPA1*0303-DPB1*8701 | HLA-DQA1*0504-DQB1*0330 |
| 1909 |  | HLA-DPA1*0303-DPB1*8801 | HLA-DQA1*0504-DQB1*0331 |
| 1910 |  | HLA-DPA1*0303-DPB1*8901 | HLA-DQA1*0504-DQB1*0332 |
| 1911 |  | HLA-DPA1*0303-DPB1*9001 | HLA-DQA1*0504-DQB1*0333 |
| 1912 |  | HLA-DPA1*0303-DPB1*9101 | HLA-DQA1*0504-DQB1*0334 |
| 1913 |  | HLA-DPA1*0303-DPB1*9201 | HLA-DQA1*0504-DQB1*0335 |
| 1914 |  | HLA-DPA1*0303-DPB1*9301 | HLA-DQA1*0504-DQB1*0336 |
| 1915 |  | HLA-DPA1*0303-DPB1*9401 | HLA-DQA1*0504-DQB1*0337 |
| 1916 |  | HLA-DPA1*0303-DPB1*9501 | HLA-DQA1*0504-DQB1*0338 |
| 1917 |  | HLA-DPA1*0303-DPB1*9601 | HLA-DQA1*0504-DQB1*0401 |
| 1918 |  | HLA-DPA1*0303-DPB1*9701 | HLA-DQA1*0504-DQB1*0402 |
| 1919 |  | HLA-DPA1*0303-DPB1*9801 | HLA-DQA1*0504-DQB1*0403 |
| 1920 |  | HLA-DPA1*0303-DPB1*9901 | HLA-DQA1*0504-DQB1*0404 |
| 1921 |  | HLA-DPA1*0401-DPB1*0101 | HLA-DQA1*0504-DQB1*0405 |
| 1922 |  | HLA-DPA1*0401-DPB1*0201 | HLA-DQA1*0504-DQB1*0406 |
| 1923 |  | HLA-DPA1*0401-DPB1*0202 | HLA-DQA1*0504-DQB1*0407 |
| 1924 |  | HLA-DPA1*0401-DPB1*0301 | HLA-DQA1*0504-DQB1*0408 |
| 1925 |  | HLA-DPA1*0401-DPB1*0401 | HLA-DQA1*0504-DQB1*0501 |
| 1926 |  | HLA-DPA1*0401-DPB1*0402 | HLA-DQA1*0504-DQB1*0502 |
| 1927 |  | HLA-DPA1*0401-DPB1*0501 | HLA-DQA1*0504-DQB1*0503 |
| 1928 |  | HLA-DPA1*0401-DPB1*0601 | HLA-DQA1*0504-DQB1*0505 |
| 1929 |  | HLA-DPA1*0401-DPB1*0801 | HLA-DQA1*0504-DQB1*0506 |
| 1930 |  | HLA-DPA1*0401-DPB1*0901 | HLA-DQA1*0504-DQB1*0507 |
| 1931 |  | HLA-DPA1*0401-DPB1*0001 | HLA-DQA1*0504-DQB1*0508 |
| 1932 |  | HLA-DPA1*0401-DPB1*1001 | HLA-DQA1*0504-DQB1*0509 |
| 1933 |  | HLA-DPA1*0401-DPB1*0101 | HLA-DQA1*0504-DQB1*0510 |
| 1934 |  | HLA-DPA1*0401-DPB1*0201 | HLA-DQA1*0504-DQB1*0511 |
| 1935 |  | HLA-DPA1*0401-DPB1*0301 | HLA-DQA1*0504-DQB1*0512 |
| 1936 |  | HLA-DPA1*0401-DPB1*0401 | HLA-DQA1*0504-DQB1*0513 |
| 1937 |  | HLA-DPA1*0401-DPB1*0501 | HLA-DQA1*0504-DQB1*0514 |
| 1938 |  | HLA-DPA1*0401-DPB1*0601 | HLA-DQA1*0504-DQB1*0601 |
| 1939 |  | HLA-DPA1*0401-DPB1*0701 | HLA-DQA1*0504-DQB1*0602 |
| 1940 |  | HLA-DPA1*0401-DPB1*0801 | HLA-DQA1*0504-DQB1*0603 |
| 1941 |  | HLA-DPA1*0401-DPB1*0901 | HLA-DQA1*0504-DQB1*0604 |
| 1942 |  | HLA-DPA1*0401-DPB1*1001 | HLA-DQA1*0504-DQB1*0607 |
| 1943 |  | HLA-DPA1*0401-DPB1*1101 | HLA-DQA1*0504-DQB1*0608 |
| 1944 |  | HLA-DPA1*0401-DPB1*1101 | HLA-DQA1*0504-DQB1*0609 |
| 1945 |  | HLA-DPA1*0401-DPB1*1201 | HLA-DQA1*0504-DQB1*0610 |
| 1946 |  | HLA-DPA1*0401-DPB1*1301 | HLA-DQA1*0504-DQB1*0611 |
| 1947 |  | HLA-DPA1*0401-DPB1*1401 | HLA-DQA1*0504-DQB1*0612 |
| 1948 |  | HLA-DPA1*0401-DPB1*1501 | HLA-DQA1*0504-DQB1*0614 |
| 1949 |  | HLA-DPA1*0401-DPB1*1601 | HLA-DQA1*0504-DQB1*0615 |
| 1950 |  | HLA-DPA1*0401-DPB1*1701 | HLA-DQA1*0504-DQB1*0616 |
| 1951 |  | HLA-DPA1*0401-DPB1*1801 | HLA-DQA1*0504-DQB1*0617 |
| 1952 |  | HLA-DPA1*0401-DPB1*1901 | HLA-DQA1*0504-DQB1*0618 |
| 1953 |  | HLA-DPA1*0401-DPB1*2101 | HLA-DQA1*0504-DQB1*0619 |
| 1954 |  | HLA-DPA1*0401-DPB1*2201 | HLA-DQA1*0504-DQB1*0621 |
| 1955 |  | HLA-DPA1*0401-DPB1*2301 | HLA-DQA1*0504-DQB1*0622 |
| 1956 |  | HLA-DPA1*0401-DPB1*2401 | HLA-DQA1*0504-DQB1*0623 |
| 1957 |  | HLA-DPA1*0401-DPB1*2501 | HLA-DQA1*0504-DQB1*0624 |
| 1958 |  | HLA-DPA1*0401-DPB1*2601 | HLA-DQA1*0504-DQB1*0625 |
| 1959 |  | HLA-DPA1*0401-DPB1*2701 | HLA-DQA1*0504-DQB1*0627 |
| 1960 |  | HLA-DPA1*0401-DPB1*2801 | HLA-DQA1*0504-DQB1*0628 |
| 1961 |  | HLA-DPA1*0401-DPB1*2901 | HLA-DQA1*0504-DQB1*0629 |
| 1962 |  | HLA-DPA1*0401-DPB1*3001 | HLA-DQA1*0504-DQB1*0630 |
| 1963 |  | HLA-DPA1*0401-DPB1*1301 | HLA-DQA1*0504-DQB1*0631 |
| 1964 |  | HLA-DPA1*0401-DPB1*3101 | HLA-DQA1*0504-DQB1*0632 |
| 1965 |  | HLA-DPA1*0401-DPB1*3201 | HLA-DQA1*0504-DQB1*0633 |
| 1966 |  | HLA-DPA1*0401-DPB1*3301 | HLA-DQA1*0504-DQB1*0634 |
| 1967 |  | HLA-DPA1*0401-DPB1*3401 | HLA-DQA1*0504-DQB1*0635 |
| 1968 |  | HLA-DPA1*0401-DPB1*1401 | HLA-DQA1*0504-DQB1*0636 |
| 1969 |  | HLA-DPA1*0401-DPB1*1501 | HLA-DQA1*0504-DQB1*0637 |
| 1970 |  | HLA-DPA1*0401-DPB1*1601 | HLA-DQA1*0504-DQB1*0638 |
| 1971 |  | HLA-DPA1*0401-DPB1*1701 | HLA-DQA1*0504-DQB1*0639 |
| 1972 |  | HLA-DPA1*0401-DPB1*1801 | HLA-DQA1*0504-DQB1*0640 |
| 1973 |  | HLA-DPA1*0401-DPB1*1901 | HLA-DQA1*0504-DQB1*0641 |
| 1974 |  | HLA-DPA1*0401-DPB1*2001 | HLA-DQA1*0504-DQB1*0642 |
| 1975 |  | HLA-DPA1*0401-DPB1*2101 | HLA-DQA1*0504-DQB1*0643 |
| 1976 |  | HLA-DPA1*0401-DPB1*2201 | HLA-DQA1*0504-DQB1*0644 |
| 1977 |  | HLA-DPA1*0401-DPB1*2301 | HLA-DQA1*0505-DQB1*0201 |
| 1978 |  | HLA-DPA1*0401-DPB1*2401 | HLA-DQA1*0505-DQB1*0202 |
| 1979 |  | HLA-DPA1*0401-DPB1*2501 | HLA-DQA1*0505-DQB1*0203 |
| 1980 |  | HLA-DPA1*0401-DPB1*2601 | HLA-DQA1*0505-DQB1*0204 |
| 1981 |  | HLA-DPA1*0401-DPB1*2701 | HLA-DQA1*0505-DQB1*0205 |
| 1982 |  | HLA-DPA1*0401-DPB1*2801 | HLA-DQA1*0505-DQB1*0206 |
| 1983 |  | HLA-DPA1*0401-DPB1*2901 | HLA-DQA1*0505-DQB1*0301 |
| 1984 |  | HLA-DPA1*0401-DPB1*3001 | HLA-DQA1*0505-DQB1*0302 |
| 1985 |  | HLA-DPA1*0401-DPB1*3101 | HLA-DQA1*0505-DQB1*0303 |
| 1986 |  | HLA-DPA1*0401-DPB1*3201 | HLA-DQA1*0505-DQB1*0304 |
| 1987 |  | HLA-DPA1*0401-DPB1*3301 | HLA-DQA1*0505-DQB1*0305 |
| 1988 |  | HLA-DPA1*0401-DPB1*3401 | HLA-DQA1*0505-DQB1*0306 |
| 1989 |  | HLA-DPA1*0401-DPB1*3501 | HLA-DQA1*0505-DQB1*0307 |
| 1990 |  | HLA-DPA1*0401-DPB1*3601 | HLA-DQA1*0505-DQB1*0308 |
| 1991 |  | HLA-DPA1*0401-DPB1*3701 | HLA-DQA1*0505-DQB1*0309 |
| 1992 |  | HLA-DPA1*0401-DPB1*3801 | HLA-DQA1*0505-DQB1*0310 |
| 1993 |  | HLA-DPA1*0401-DPB1*3901 | HLA-DQA1*0505-DQB1*0311 |
| 1994 |  | HLA-DPA1*0401-DPB1*4001 | HLA-DQA1*0505-DQB1*0312 |
| 1995 |  | HLA-DPA1*0401-DPB1*4101 | HLA-DQA1*0505-DQB1*0313 |
| 1996 |  | HLA-DPA1*0401-DPB1*4401 | HLA-DQA1*0505-DQB1*0314 |
| 1997 |  | HLA-DPA1*0401-DPB1*4501 | HLA-DQA1*0505-DQB1*0315 |
| 1998 |  | HLA-DPA1*0401-DPB1*4601 | HLA-DQA1*0505-DQB1*0316 |
| 1999 |  | HLA-DPA1*0401-DPB1*4701 | HLA-DQA1*0505-DQB1*0317 |
| 2000 |  | HLA-DPA1*0401-DPB1*4801 | HLA-DQA1*0505-DQB1*0318 |
| 2001 |  | HLA-DPA1*0401-DPB1*4901 | HLA-DQA1*0505-DQB1*0319 |
| 2002 |  | HLA-DPA1*0401-DPB1*5001 | HLA-DQA1*0505-DQB1*0320 |
| 2003 |  | HLA-DPA1*0401-DPB1*5101 | HLA-DQA1*0505-DQB1*0321 |
| 2004 |  | HLA-DPA1*0401-DPB1*5201 | HLA-DQA1*0505-DQB1*0322 |
| 2005 |  | HLA-DPA1*0401-DPB1*5301 | HLA-DQA1*0505-DQB1*0323 |
| 2006 |  | HLA-DPA1*0401-DPB1*5401 | HLA-DQA1*0505-DQB1*0324 |
| 2007 |  | HLA-DPA1*0401-DPB1*5501 | HLA-DQA1*0505-DQB1*0325 |
| 2008 |  | HLA-DPA1*0401-DPB1*5601 | HLA-DQA1*0505-DQB1*0326 |
| 2009 |  | HLA-DPA1*0401-DPB1*5801 | HLA-DQA1*0505-DQB1*0327 |
| 2010 |  | HLA-DPA1*0401-DPB1*5901 | HLA-DQA1*0505-DQB1*0328 |
| 2011 |  | HLA-DPA1*0401-DPB1*6001 | HLA-DQA1*0505-DQB1*0329 |
| 2012 |  | HLA-DPA1*0401-DPB1*6201 | HLA-DQA1*0505-DQB1*0330 |
| 2013 |  | HLA-DPA1*0401-DPB1*6301 | HLA-DQA1*0505-DQB1*0331 |
| 2014 |  | HLA-DPA1*0401-DPB1*6501 | HLA-DQA1*0505-DQB1*0332 |
| 2015 |  | HLA-DPA1*0401-DPB1*6601 | HLA-DQA1*0505-DQB1*0333 |
| 2016 |  | HLA-DPA1*0401-DPB1*6701 | HLA-DQA1*0505-DQB1*0334 |
| 2017 |  | HLA-DPA1*0401-DPB1*6801 | HLA-DQA1*0505-DQB1*0335 |
| 2018 |  | HLA-DPA1*0401-DPB1*6901 | HLA-DQA1*0505-DQB1*0336 |
| 2019 |  | HLA-DPA1*0401-DPB1*7001 | HLA-DQA1*0505-DQB1*0337 |
| 2020 |  | HLA-DPA1*0401-DPB1*7101 | HLA-DQA1*0505-DQB1*0338 |
| 2021 |  | HLA-DPA1*0401-DPB1*7201 | HLA-DQA1*0505-DQB1*0401 |
| 2022 |  | HLA-DPA1*0401-DPB1*7301 | HLA-DQA1*0505-DQB1*0402 |
| 2023 |  | HLA-DPA1*0401-DPB1*7401 | HLA-DQA1*0505-DQB1*0403 |
| 2024 |  | HLA-DPA1*0401-DPB1*7501 | HLA-DQA1*0505-DQB1*0404 |
| 2025 |  | HLA-DPA1*0401-DPB1*7601 | HLA-DQA1*0505-DQB1*0405 |
| 2026 |  | HLA-DPA1*0401-DPB1*7701 | HLA-DQA1*0505-DQB1*0406 |
| 2027 |  | HLA-DPA1*0401-DPB1*7801 | HLA-DQA1*0505-DQB1*0407 |
| 2028 |  | HLA-DPA1*0401-DPB1*7901 | HLA-DQA1*0505-DQB1*0408 |
| 2029 |  | HLA-DPA1*0401-DPB1*8001 | HLA-DQA1*0505-DQB1*0501 |
| 2030 |  | HLA-DPA1*0401-DPB1*8101 | HLA-DQA1*0505-DQB1*0502 |
| 2031 |  | HLA-DPA1*0401-DPB1*8201 | HLA-DQA1*0505-DQB1*0503 |
| 2032 |  | HLA-DPA1*0401-DPB1*8301 | HLA-DQA1*0505-DQB1*0505 |
| 2033 |  | HLA-DPA1*0401-DPB1*8401 | HLA-DQA1*0505-DQB1*0506 |
| 2034 |  | HLA-DPA1*0401-DPB1*8501 | HLA-DQA1*0505-DQB1*0507 |
| 2035 |  | HLA-DPA1*0401-DPB1*8601 | HLA-DQA1*0505-DQB1*0508 |
| 2036 |  | HLA-DPA1*0401-DPB1*8701 | HLA-DQA1*0505-DQB1*0509 |
| 2037 |  | HLA-DPA1*0401-DPB1*8801 | HLA-DQA1*0505-DQB1*0510 |
| 2038 |  | HLA-DPA1*0401-DPB1*8901 | HLA-DQA1*0505-DQB1*0511 |
| 2039 |  | HLA-DPA1*0401-DPB1*9001 | HLA-DQA1*0505-DQB1*0512 |
| 2040 |  | HLA-DPA1*0401-DPB1*9101 | HLA-DQA1*0505-DQB1*0513 |
| 2041 |  | HLA-DPA1*0401-DPB1*9201 | HLA-DQA1*0505-DQB1*0514 |
| 2042 |  | HLA-DPA1*0401-DPB1*9301 | HLA-DQA1*0505-DQB1*0601 |
| 2043 |  | HLA-DPA1*0401-DPB1*9401 | HLA-DQA1*0505-DQB1*0602 |
| 2044 |  | HLA-DPA1*0401-DPB1*9501 | HLA-DQA1*0505-DQB1*0603 |
| 2045 |  | HLA-DPA1*0401-DPB1*9601 | HLA-DQA1*0505-DQB1*0604 |
| 2046 |  | HLA-DPA1*0401-DPB1*9701 | HLA-DQA1*0505-DQB1*0607 |
| 2047 |  | HLA-DPA1*0401-DPB1*9801 | HLA-DQA1*0505-DQB1*0608 |
| 2048 |  | HLA-DPA1*0401-DPB1*9901 | HLA-DQA1*0505-DQB1*0609 |
| 2049 |  |  | HLA-DQA1*0505-DQB1*0610 |
| 2050 |  |  | HLA-DQA1*0505-DQB1*0611 |
| 2051 |  |  | HLA-DQA1*0505-DQB1*0612 |
| 2052 |  |  | HLA-DQA1*0505-DQB1*0614 |
| 2053 |  |  | HLA-DQA1*0505-DQB1*0615 |
| 2054 |  |  | HLA-DQA1*0505-DQB1*0616 |
| 2055 |  |  | HLA-DQA1*0505-DQB1*0617 |
| 2056 |  |  | HLA-DQA1*0505-DQB1*0618 |
| 2057 |  |  | HLA-DQA1*0505-DQB1*0619 |
| 2058 |  |  | HLA-DQA1*0505-DQB1*0621 |
| 2059 |  |  | HLA-DQA1*0505-DQB1*0622 |
| 2060 |  |  | HLA-DQA1*0505-DQB1*0623 |
| 2061 |  |  | HLA-DQA1*0505-DQB1*0624 |
| 2062 |  |  | HLA-DQA1*0505-DQB1*0625 |
| 2063 |  |  | HLA-DQA1*0505-DQB1*0627 |
| 2064 |  |  | HLA-DQA1*0505-DQB1*0628 |
| 2065 |  |  | HLA-DQA1*0505-DQB1*0629 |
| 2066 |  |  | HLA-DQA1*0505-DQB1*0630 |
| 2067 |  |  | HLA-DQA1*0505-DQB1*0631 |
| 2068 |  |  | HLA-DQA1*0505-DQB1*0632 |
| 2069 |  |  | HLA-DQA1*0505-DQB1*0633 |
| 2070 |  |  | HLA-DQA1*0505-DQB1*0634 |
| 2071 |  |  | HLA-DQA1*0505-DQB1*0635 |
| 2072 |  |  | HLA-DQA1*0505-DQB1*0636 |
| 2073 |  |  | HLA-DQA1*0505-DQB1*0637 |
| 2074 |  |  | HLA-DQA1*0505-DQB1*0638 |
| 2075 |  |  | HLA-DQA1*0505-DQB1*0639 |
| 2076 |  |  | HLA-DQA1*0505-DQB1*0640 |
| 2077 |  |  | HLA-DQA1*0505-DQB1*0641 |
| 2078 |  |  | HLA-DQA1*0505-DQB1*0642 |
| 2079 |  |  | HLA-DQA1*0505-DQB1*0643 |
| 2080 |  |  | HLA-DQA1*0505-DQB1*0644 |
| 2081 |  |  | HLA-DQA1*0506-DQB1*0201 |
| 2082 |  |  | HLA-DQA1*0506-DQB1*0202 |
| 2083 |  |  | HLA-DQA1*0506-DQB1*0203 |
| 2084 |  |  | HLA-DQA1*0506-DQB1*0204 |
| 2085 |  |  | HLA-DQA1*0506-DQB1*0205 |
| 2086 |  |  | HLA-DQA1*0506-DQB1*0206 |
| 2087 |  |  | HLA-DQA1*0506-DQB1*0301 |
| 2088 |  |  | HLA-DQA1*0506-DQB1*0302 |
| 2089 |  |  | HLA-DQA1*0506-DQB1*0303 |
| 2090 |  |  | HLA-DQA1*0506-DQB1*0304 |
| 2091 |  |  | HLA-DQA1*0506-DQB1*0305 |
| 2092 |  |  | HLA-DQA1*0506-DQB1*0306 |
| 2093 |  |  | HLA-DQA1*0506-DQB1*0307 |
| 2094 |  |  | HLA-DQA1*0506-DQB1*0308 |
| 2095 |  |  | HLA-DQA1*0506-DQB1*0309 |
| 2096 |  |  | HLA-DQA1*0506-DQB1*0310 |
| 2097 |  |  | HLA-DQA1*0506-DQB1*0311 |
| 2098 |  |  | HLA-DQA1*0506-DQB1*0312 |
| 2099 |  |  | HLA-DQA1*0506-DQB1*0313 |
| 2100 |  |  | HLA-DQA1*0506-DQB1*0314 |
| 2101 |  |  | HLA-DQA1*0506-DQB1*0315 |
| 2102 |  |  | HLA-DQA1*0506-DQB1*0316 |
| 2103 |  |  | HLA-DQA1*0506-DQB1*0317 |
| 2104 |  |  | HLA-DQA1*0506-DQB1*0318 |
| 2105 |  |  | HLA-DQA1*0506-DQB1*0319 |
| 2106 |  |  | HLA-DQA1*0506-DQB1*0320 |
| 2107 |  |  | HLA-DQA1*0506-DQB1*0321 |
| 2108 |  |  | HLA-DQA1*0506-DQB1*0322 |
| 2109 |  |  | HLA-DQA1*0506-DQB1*0323 |
| 2110 |  |  | HLA-DQA1*0506-DQB1*0324 |
| 2111 |  |  | HLA-DQA1*0506-DQB1*0325 |
| 2112 |  |  | HLA-DQA1*0506-DQB1*0326 |
| 2113 |  |  | HLA-DQA1*0506-DQB1*0327 |
| 2114 |  |  | HLA-DQA1*0506-DQB1*0328 |
| 2115 |  |  | HLA-DQA1*0506-DQB1*0329 |
| 2116 |  |  | HLA-DQA1*0506-DQB1*0330 |
| 2117 |  |  | HLA-DQA1*0506-DQB1*0331 |
| 2118 |  |  | HLA-DQA1*0506-DQB1*0332 |
| 2119 |  |  | HLA-DQA1*0506-DQB1*0333 |
| 2120 |  |  | HLA-DQA1*0506-DQB1*0334 |
| 2121 |  |  | HLA-DQA1*0506-DQB1*0335 |
| 2122 |  |  | HLA-DQA1*0506-DQB1*0336 |
| 2123 |  |  | HLA-DQA1*0506-DQB1*0337 |
| 2124 |  |  | HLA-DQA1*0506-DQB1*0338 |
| 2125 |  |  | HLA-DQA1*0506-DQB1*0401 |
| 2126 |  |  | HLA-DQA1*0506-DQB1*0402 |
| 2127 |  |  | HLA-DQA1*0506-DQB1*0403 |
| 2128 |  |  | HLA-DQA1*0506-DQB1*0404 |
| 2129 |  |  | HLA-DQA1*0506-DQB1*0405 |
| 2130 |  |  | HLA-DQA1*0506-DQB1*0406 |
| 2131 |  |  | HLA-DQA1*0506-DQB1*0407 |
| 2132 |  |  | HLA-DQA1*0506-DQB1*0408 |
| 2133 |  |  | HLA-DQA1*0506-DQB1*0501 |
| 2134 |  |  | HLA-DQA1*0506-DQB1*0502 |
| 2135 |  |  | HLA-DQA1*0506-DQB1*0503 |
| 2136 |  |  | HLA-DQA1*0506-DQB1*0505 |
| 2137 |  |  | HLA-DQA1*0506-DQB1*0506 |
| 2138 |  |  | HLA-DQA1*0506-DQB1*0507 |
| 2139 |  |  | HLA-DQA1*0506-DQB1*0508 |
| 2140 |  |  | HLA-DQA1*0506-DQB1*0509 |
| 2141 |  |  | HLA-DQA1*0506-DQB1*0510 |
| 2142 |  |  | HLA-DQA1*0506-DQB1*0511 |
| 2143 |  |  | HLA-DQA1*0506-DQB1*0512 |
| 2144 |  |  | HLA-DQA1*0506-DQB1*0513 |
| 2145 |  |  | HLA-DQA1*0506-DQB1*0514 |
| 2146 |  |  | HLA-DQA1*0506-DQB1*0601 |
| 2147 |  |  | HLA-DQA1*0506-DQB1*0602 |
| 2148 |  |  | HLA-DQA1*0506-DQB1*0603 |
| 2149 |  |  | HLA-DQA1*0506-DQB1*0604 |
| 2150 |  |  | HLA-DQA1*0506-DQB1*0607 |
| 2151 |  |  | HLA-DQA1*0506-DQB1*0608 |
| 2152 |  |  | HLA-DQA1*0506-DQB1*0609 |
| 2153 |  |  | HLA-DQA1*0506-DQB1*0610 |
| 2154 |  |  | HLA-DQA1*0506-DQB1*0611 |
| 2155 |  |  | HLA-DQA1*0506-DQB1*0612 |
| 2156 |  |  | HLA-DQA1*0506-DQB1*0614 |
| 2157 |  |  | HLA-DQA1*0506-DQB1*0615 |
| 2158 |  |  | HLA-DQA1*0506-DQB1*0616 |
| 2159 |  |  | HLA-DQA1*0506-DQB1*0617 |
| 2160 |  |  | HLA-DQA1*0506-DQB1*0618 |
| 2161 |  |  | HLA-DQA1*0506-DQB1*0619 |
| 2162 |  |  | HLA-DQA1*0506-DQB1*0621 |
| 2163 |  |  | HLA-DQA1*0506-DQB1*0622 |
| 2164 |  |  | HLA-DQA1*0506-DQB1*0623 |
| 2165 |  |  | HLA-DQA1*0506-DQB1*0624 |
| 2166 |  |  | HLA-DQA1*0506-DQB1*0625 |
| 2167 |  |  | HLA-DQA1*0506-DQB1*0627 |
| 2168 |  |  | HLA-DQA1*0506-DQB1*0628 |
| 2169 |  |  | HLA-DQA1*0506-DQB1*0629 |
| 2170 |  |  | HLA-DQA1*0506-DQB1*0630 |
| 2171 |  |  | HLA-DQA1*0506-DQB1*0631 |
| 2172 |  |  | HLA-DQA1*0506-DQB1*0632 |
| 2173 |  |  | HLA-DQA1*0506-DQB1*0633 |
| 2174 |  |  | HLA-DQA1*0506-DQB1*0634 |
| 2175 |  |  | HLA-DQA1*0506-DQB1*0635 |
| 2176 |  |  | HLA-DQA1*0506-DQB1*0636 |
| 2177 |  |  | HLA-DQA1*0506-DQB1*0637 |
| 2178 |  |  | HLA-DQA1*0506-DQB1*0638 |
| 2179 |  |  | HLA-DQA1*0506-DQB1*0639 |
| 2180 |  |  | HLA-DQA1*0506-DQB1*0640 |
| 2181 |  |  | HLA-DQA1*0506-DQB1*0641 |
| 2182 |  |  | HLA-DQA1*0506-DQB1*0642 |
| 2183 |  |  | HLA-DQA1*0506-DQB1*0643 |
| 2184 |  |  | HLA-DQA1*0506-DQB1*0644 |
| 2185 |  |  | HLA-DQA1*0507-DQB1*0201 |
| 2186 |  |  | HLA-DQA1*0507-DQB1*0202 |
| 2187 |  |  | HLA-DQA1*0507-DQB1*0203 |
| 2188 |  |  | HLA-DQA1*0507-DQB1*0204 |
| 2189 |  |  | HLA-DQA1*0507-DQB1*0205 |
| 2190 |  |  | HLA-DQA1*0507-DQB1*0206 |
| 2191 |  |  | HLA-DQA1*0507-DQB1*0301 |
| 2192 |  |  | HLA-DQA1*0507-DQB1*0302 |
| 2193 |  |  | HLA-DQA1*0507-DQB1*0303 |
| 2194 |  |  | HLA-DQA1*0507-DQB1*0304 |
| 2195 |  |  | HLA-DQA1*0507-DQB1*0305 |
| 2196 |  |  | HLA-DQA1*0507-DQB1*0306 |
| 2197 |  |  | HLA-DQA1*0507-DQB1*0307 |
| 2198 |  |  | HLA-DQA1*0507-DQB1*0308 |
| 2199 |  |  | HLA-DQA1*0507-DQB1*0309 |
| 2200 |  |  | HLA-DQA1*0507-DQB1*0310 |
| 2201 |  |  | HLA-DQA1*0507-DQB1*0311 |
| 2202 |  |  | HLA-DQA1*0507-DQB1*0312 |
| 2203 |  |  | HLA-DQA1*0507-DQB1*0313 |
| 2204 |  |  | HLA-DQA1*0507-DQB1*0314 |
| 2205 |  |  | HLA-DQA1*0507-DQB1*0315 |
| 2206 |  |  | HLA-DQA1*0507-DQB1*0316 |
| 2207 |  |  | HLA-DQA1*0507-DQB1*0317 |
| 2208 |  |  | HLA-DQA1*0507-DQB1*0318 |
| 2209 |  |  | HLA-DQA1*0507-DQB1*0319 |
| 2210 |  |  | HLA-DQA1*0507-DQB1*0320 |
| 2211 |  |  | HLA-DQA1*0507-DQB1*0321 |
| 2212 |  |  | HLA-DQA1*0507-DQB1*0322 |
| 2213 |  |  | HLA-DQA1*0507-DQB1*0323 |
| 2214 |  |  | HLA-DQA1*0507-DQB1*0324 |
| 2215 |  |  | HLA-DQA1*0507-DQB1*0325 |
| 2216 |  |  | HLA-DQA1*0507-DQB1*0326 |
| 2217 |  |  | HLA-DQA1*0507-DQB1*0327 |
| 2218 |  |  | HLA-DQA1*0507-DQB1*0328 |
| 2219 |  |  | HLA-DQA1*0507-DQB1*0329 |
| 2220 |  |  | HLA-DQA1*0507-DQB1*0330 |
| 2221 |  |  | HLA-DQA1*0507-DQB1*0331 |
| 2222 |  |  | HLA-DQA1*0507-DQB1*0332 |
| 2223 |  |  | HLA-DQA1*0507-DQB1*0333 |
| 2224 |  |  | HLA-DQA1*0507-DQB1*0334 |
| 2225 |  |  | HLA-DQA1*0507-DQB1*0335 |
| 2226 |  |  | HLA-DQA1*0507-DQB1*0336 |
| 2227 |  |  | HLA-DQA1*0507-DQB1*0337 |
| 2228 |  |  | HLA-DQA1*0507-DQB1*0338 |
| 2229 |  |  | HLA-DQA1*0507-DQB1*0401 |
| 2230 |  |  | HLA-DQA1*0507-DQB1*0402 |
| 2231 |  |  | HLA-DQA1*0507-DQB1*0403 |
| 2232 |  |  | HLA-DQA1*0507-DQB1*0404 |
| 2233 |  |  | HLA-DQA1*0507-DQB1*0405 |
| 2234 |  |  | HLA-DQA1*0507-DQB1*0406 |
| 2235 |  |  | HLA-DQA1*0507-DQB1*0407 |
| 2236 |  |  | HLA-DQA1*0507-DQB1*0408 |
| 2237 |  |  | HLA-DQA1*0507-DQB1*0501 |
| 2238 |  |  | HLA-DQA1*0507-DQB1*0502 |
| 2239 |  |  | HLA-DQA1*0507-DQB1*0503 |
| 2240 |  |  | HLA-DQA1*0507-DQB1*0505 |
| 2241 |  |  | HLA-DQA1*0507-DQB1*0506 |
| 2242 |  |  | HLA-DQA1*0507-DQB1*0507 |
| 2243 |  |  | HLA-DQA1*0507-DQB1*0508 |
| 2244 |  |  | HLA-DQA1*0507-DQB1*0509 |
| 2245 |  |  | HLA-DQA1*0507-DQB1*0510 |
| 2246 |  |  | HLA-DQA1*0507-DQB1*0511 |
| 2247 |  |  | HLA-DQA1*0507-DQB1*0512 |
| 2248 |  |  | HLA-DQA1*0507-DQB1*0513 |
| 2249 |  |  | HLA-DQA1*0507-DQB1*0514 |
| 2250 |  |  | HLA-DQA1*0507-DQB1*0601 |
| 2251 |  |  | HLA-DQA1*0507-DQB1*0602 |
| 2252 |  |  | HLA-DQA1*0507-DQB1*0603 |
| 2253 |  |  | HLA-DQA1*0507-DQB1*0604 |
| 2254 |  |  | HLA-DQA1*0507-DQB1*0607 |
| 2255 |  |  | HLA-DQA1*0507-DQB1*0608 |
| 2256 |  |  | HLA-DQA1*0507-DQB1*0609 |
| 2257 |  |  | HLA-DQA1*0507-DQB1*0610 |
| 2258 |  |  | HLA-DQA1*0507-DQB1*0611 |
| 2259 |  |  | HLA-DQA1*0507-DQB1*0612 |
| 2260 |  |  | HLA-DQA1*0507-DQB1*0614 |
| 2261 |  |  | HLA-DQA1*0507-DQB1*0615 |
| 2262 |  |  | HLA-DQA1*0507-DQB1*0616 |
| 2263 |  |  | HLA-DQA1*0507-DQB1*0617 |
| 2264 |  |  | HLA-DQA1*0507-DQB1*0618 |
| 2265 |  |  | HLA-DQA1*0507-DQB1*0619 |
| 2266 |  |  | HLA-DQA1*0507-DQB1*0621 |
| 2267 |  |  | HLA-DQA1*0507-DQB1*0622 |
| 2268 |  |  | HLA-DQA1*0507-DQB1*0623 |
| 2269 |  |  | HLA-DQA1*0507-DQB1*0624 |
| 2270 |  |  | HLA-DQA1*0507-DQB1*0625 |
| 2271 |  |  | HLA-DQA1*0507-DQB1*0627 |
| 2272 |  |  | HLA-DQA1*0507-DQB1*0628 |
| 2273 |  |  | HLA-DQA1*0507-DQB1*0629 |
| 2274 |  |  | HLA-DQA1*0507-DQB1*0630 |
| 2275 |  |  | HLA-DQA1*0507-DQB1*0631 |
| 2276 |  |  | HLA-DQA1*0507-DQB1*0632 |
| 2277 |  |  | HLA-DQA1*0507-DQB1*0633 |
| 2278 |  |  | HLA-DQA1*0507-DQB1*0634 |
| 2279 |  |  | HLA-DQA1*0507-DQB1*0635 |
| 2280 |  |  | HLA-DQA1*0507-DQB1*0636 |
| 2281 |  |  | HLA-DQA1*0507-DQB1*0637 |
| 2282 |  |  | HLA-DQA1*0507-DQB1*0638 |
| 2283 |  |  | HLA-DQA1*0507-DQB1*0639 |
| 2284 |  |  | HLA-DQA1*0507-DQB1*0640 |
| 2285 |  |  | HLA-DQA1*0507-DQB1*0641 |
| 2286 |  |  | HLA-DQA1*0507-DQB1*0642 |
| 2287 |  |  | HLA-DQA1*0507-DQB1*0643 |
| 2288 |  |  | HLA-DQA1*0507-DQB1*0644 |
| 2289 |  |  | HLA-DQA1*0508-DQB1*0201 |
| 2290 |  |  | HLA-DQA1*0508-DQB1*0202 |
| 2291 |  |  | HLA-DQA1*0508-DQB1*0203 |
| 2292 |  |  | HLA-DQA1*0508-DQB1*0204 |
| 2293 |  |  | HLA-DQA1*0508-DQB1*0205 |
| 2294 |  |  | HLA-DQA1*0508-DQB1*0206 |
| 2295 |  |  | HLA-DQA1*0508-DQB1*0301 |
| 2296 |  |  | HLA-DQA1*0508-DQB1*0302 |
| 2297 |  |  | HLA-DQA1*0508-DQB1*0303 |
| 2298 |  |  | HLA-DQA1*0508-DQB1*0304 |
| 2299 |  |  | HLA-DQA1*0508-DQB1*0305 |
| 2300 |  |  | HLA-DQA1*0508-DQB1*0306 |
| 2301 |  |  | HLA-DQA1*0508-DQB1*0307 |
| 2302 |  |  | HLA-DQA1*0508-DQB1*0308 |
| 2303 |  |  | HLA-DQA1*0508-DQB1*0309 |
| 2304 |  |  | HLA-DQA1*0508-DQB1*0310 |
| 2305 |  |  | HLA-DQA1*0508-DQB1*0311 |
| 2306 |  |  | HLA-DQA1*0508-DQB1*0312 |
| 2307 |  |  | HLA-DQA1*0508-DQB1*0313 |
| 2308 |  |  | HLA-DQA1*0508-DQB1*0314 |
| 2309 |  |  | HLA-DQA1*0508-DQB1*0315 |
| 2310 |  |  | HLA-DQA1*0508-DQB1*0316 |
| 2311 |  |  | HLA-DQA1*0508-DQB1*0317 |
| 2312 |  |  | HLA-DQA1*0508-DQB1*0318 |
| 2313 |  |  | HLA-DQA1*0508-DQB1*0319 |
| 2314 |  |  | HLA-DQA1*0508-DQB1*0320 |
| 2315 |  |  | HLA-DQA1*0508-DQB1*0321 |
| 2316 |  |  | HLA-DQA1*0508-DQB1*0322 |
| 2317 |  |  | HLA-DQA1*0508-DQB1*0323 |
| 2318 |  |  | HLA-DQA1*0508-DQB1*0324 |
| 2319 |  |  | HLA-DQA1*0508-DQB1*0325 |
| 2320 |  |  | HLA-DQA1*0508-DQB1*0326 |
| 2321 |  |  | HLA-DQA1*0508-DQB1*0327 |
| 2322 |  |  | HLA-DQA1*0508-DQB1*0328 |
| 2323 |  |  | HLA-DQA1*0508-DQB1*0329 |
| 2324 |  |  | HLA-DQA1*0508-DQB1*0330 |
| 2325 |  |  | HLA-DQA1*0508-DQB1*0331 |
| 2326 |  |  | HLA-DQA1*0508-DQB1*0332 |
| 2327 |  |  | HLA-DQA1*0508-DQB1*0333 |
| 2328 |  |  | HLA-DQA1*0508-DQB1*0334 |
| 2329 |  |  | HLA-DQA1*0508-DQB1*0335 |
| 2330 |  |  | HLA-DQA1*0508-DQB1*0336 |
| 2331 |  |  | HLA-DQA1*0508-DQB1*0337 |
| 2332 |  |  | HLA-DQA1*0508-DQB1*0338 |
| 2333 |  |  | HLA-DQA1*0508-DQB1*0401 |
| 2334 |  |  | HLA-DQA1*0508-DQB1*0402 |
| 2335 |  |  | HLA-DQA1*0508-DQB1*0403 |
| 2336 |  |  | HLA-DQA1*0508-DQB1*0404 |
| 2337 |  |  | HLA-DQA1*0508-DQB1*0405 |
| 2338 |  |  | HLA-DQA1*0508-DQB1*0406 |
| 2339 |  |  | HLA-DQA1*0508-DQB1*0407 |
| 2340 |  |  | HLA-DQA1*0508-DQB1*0408 |
| 2341 |  |  | HLA-DQA1*0508-DQB1*0501 |
| 2342 |  |  | HLA-DQA1*0508-DQB1*0502 |
| 2343 |  |  | HLA-DQA1*0508-DQB1*0503 |
| 2344 |  |  | HLA-DQA1*0508-DQB1*0505 |
| 2345 |  |  | HLA-DQA1*0508-DQB1*0506 |
| 2346 |  |  | HLA-DQA1*0508-DQB1*0507 |
| 2347 |  |  | HLA-DQA1*0508-DQB1*0508 |
| 2348 |  |  | HLA-DQA1*0508-DQB1*0509 |
| 2349 |  |  | HLA-DQA1*0508-DQB1*0510 |
| 2350 |  |  | HLA-DQA1*0508-DQB1*0511 |
| 2351 |  |  | HLA-DQA1*0508-DQB1*0512 |
| 2352 |  |  | HLA-DQA1*0508-DQB1*0513 |
| 2353 |  |  | HLA-DQA1*0508-DQB1*0514 |
| 2354 |  |  | HLA-DQA1*0508-DQB1*0601 |
| 2355 |  |  | HLA-DQA1*0508-DQB1*0602 |
| 2356 |  |  | HLA-DQA1*0508-DQB1*0603 |
| 2357 |  |  | HLA-DQA1*0508-DQB1*0604 |
| 2358 |  |  | HLA-DQA1*0508-DQB1*0607 |
| 2359 |  |  | HLA-DQA1*0508-DQB1*0608 |
| 2360 |  |  | HLA-DQA1*0508-DQB1*0609 |
| 2361 |  |  | HLA-DQA1*0508-DQB1*0610 |
| 2362 |  |  | HLA-DQA1*0508-DQB1*0611 |
| 2363 |  |  | HLA-DQA1*0508-DQB1*0612 |
| 2364 |  |  | HLA-DQA1*0508-DQB1*0614 |
| 2365 |  |  | HLA-DQA1*0508-DQB1*0615 |
| 2366 |  |  | HLA-DQA1*0508-DQB1*0616 |
| 2367 |  |  | HLA-DQA1*0508-DQB1*0617 |
| 2368 |  |  | HLA-DQA1*0508-DQB1*0618 |
| 2369 |  |  | HLA-DQA1*0508-DQB1*0619 |
| 2370 |  |  | HLA-DQA1*0508-DQB1*0621 |
| 2371 |  |  | HLA-DQA1*0508-DQB1*0622 |
| 2372 |  |  | HLA-DQA1*0508-DQB1*0623 |
| 2373 |  |  | HLA-DQA1*0508-DQB1*0624 |
| 2374 |  |  | HLA-DQA1*0508-DQB1*0625 |
| 2375 |  |  | HLA-DQA1*0508-DQB1*0627 |
| 2376 |  |  | HLA-DQA1*0508-DQB1*0628 |
| 2377 |  |  | HLA-DQA1*0508-DQB1*0629 |
| 2378 |  |  | HLA-DQA1*0508-DQB1*0630 |
| 2379 |  |  | HLA-DQA1*0508-DQB1*0631 |
| 2380 |  |  | HLA-DQA1*0508-DQB1*0632 |
| 2381 |  |  | HLA-DQA1*0508-DQB1*0633 |
| 2382 |  |  | HLA-DQA1*0508-DQB1*0634 |
| 2383 |  |  | HLA-DQA1*0508-DQB1*0635 |
| 2384 |  |  | HLA-DQA1*0508-DQB1*0636 |
| 2385 |  |  | HLA-DQA1*0508-DQB1*0637 |
| 2386 |  |  | HLA-DQA1*0508-DQB1*0638 |
| 2387 |  |  | HLA-DQA1*0508-DQB1*0639 |
| 2388 |  |  | HLA-DQA1*0508-DQB1*0640 |
| 2389 |  |  | HLA-DQA1*0508-DQB1*0641 |
| 2390 |  |  | HLA-DQA1*0508-DQB1*0642 |
| 2391 |  |  | HLA-DQA1*0508-DQB1*0643 |
| 2392 |  |  | HLA-DQA1*0508-DQB1*0644 |
| 2393 |  |  | HLA-DQA1*0509-DQB1*0201 |
| 2394 |  |  | HLA-DQA1*0509-DQB1*0202 |
| 2395 |  |  | HLA-DQA1*0509-DQB1*0203 |
| 2396 |  |  | HLA-DQA1*0509-DQB1*0204 |
| 2397 |  |  | HLA-DQA1*0509-DQB1*0205 |
| 2398 |  |  | HLA-DQA1*0509-DQB1*0206 |
| 2399 |  |  | HLA-DQA1*0509-DQB1*0301 |
| 2400 |  |  | HLA-DQA1*0509-DQB1*0302 |
| 2401 |  |  | HLA-DQA1*0509-DQB1*0303 |
| 2402 |  |  | HLA-DQA1*0509-DQB1*0304 |
| 2403 |  |  | HLA-DQA1*0509-DQB1*0305 |
| 2404 |  |  | HLA-DQA1*0509-DQB1*0306 |
| 2405 |  |  | HLA-DQA1*0509-DQB1*0307 |
| 2406 |  |  | HLA-DQA1*0509-DQB1*0308 |
| 2407 |  |  | HLA-DQA1*0509-DQB1*0309 |
| 2408 |  |  | HLA-DQA1*0509-DQB1*0310 |
| 2409 |  |  | HLA-DQA1*0509-DQB1*0311 |
| 2410 |  |  | HLA-DQA1*0509-DQB1*0312 |
| 2411 |  |  | HLA-DQA1*0509-DQB1*0313 |
| 2412 |  |  | HLA-DQA1*0509-DQB1*0314 |
| 2413 |  |  | HLA-DQA1*0509-DQB1*0315 |
| 2414 |  |  | HLA-DQA1*0509-DQB1*0316 |
| 2415 |  |  | HLA-DQA1*0509-DQB1*0317 |
| 2416 |  |  | HLA-DQA1*0509-DQB1*0318 |
| 2417 |  |  | HLA-DQA1*0509-DQB1*0319 |
| 2418 |  |  | HLA-DQA1*0509-DQB1*0320 |
| 2419 |  |  | HLA-DQA1*0509-DQB1*0321 |
| 2420 |  |  | HLA-DQA1*0509-DQB1*0322 |
| 2421 |  |  | HLA-DQA1*0509-DQB1*0323 |
| 2422 |  |  | HLA-DQA1*0509-DQB1*0324 |
| 2423 |  |  | HLA-DQA1*0509-DQB1*0325 |
| 2424 |  |  | HLA-DQA1*0509-DQB1*0326 |
| 2425 |  |  | HLA-DQA1*0509-DQB1*0327 |
| 2426 |  |  | HLA-DQA1*0509-DQB1*0328 |
| 2427 |  |  | HLA-DQA1*0509-DQB1*0329 |
| 2428 |  |  | HLA-DQA1*0509-DQB1*0330 |
| 2429 |  |  | HLA-DQA1*0509-DQB1*0331 |
| 2430 |  |  | HLA-DQA1*0509-DQB1*0332 |
| 2431 |  |  | HLA-DQA1*0509-DQB1*0333 |
| 2432 |  |  | HLA-DQA1*0509-DQB1*0334 |
| 2433 |  |  | HLA-DQA1*0509-DQB1*0335 |
| 2434 |  |  | HLA-DQA1*0509-DQB1*0336 |
| 2435 |  |  | HLA-DQA1*0509-DQB1*0337 |
| 2436 |  |  | HLA-DQA1*0509-DQB1*0338 |
| 2437 |  |  | HLA-DQA1*0509-DQB1*0401 |
| 2438 |  |  | HLA-DQA1*0509-DQB1*0402 |
| 2439 |  |  | HLA-DQA1*0509-DQB1*0403 |
| 2440 |  |  | HLA-DQA1*0509-DQB1*0404 |
| 2441 |  |  | HLA-DQA1*0509-DQB1*0405 |
| 2442 |  |  | HLA-DQA1*0509-DQB1*0406 |
| 2443 |  |  | HLA-DQA1*0509-DQB1*0407 |
| 2444 |  |  | HLA-DQA1*0509-DQB1*0408 |
| 2445 |  |  | HLA-DQA1*0509-DQB1*0501 |
| 2446 |  |  | HLA-DQA1*0509-DQB1*0502 |
| 2447 |  |  | HLA-DQA1*0509-DQB1*0503 |
| 2448 |  |  | HLA-DQA1*0509-DQB1*0505 |
| 2449 |  |  | HLA-DQA1*0509-DQB1*0506 |
| 2450 |  |  | HLA-DQA1*0509-DQB1*0507 |
| 2451 |  |  | HLA-DQA1*0509-DQB1*0508 |
| 2452 |  |  | HLA-DQA1*0509-DQB1*0509 |
| 2453 |  |  | HLA-DQA1*0509-DQB1*0510 |
| 2454 |  |  | HLA-DQA1*0509-DQB1*0511 |
| 2455 |  |  | HLA-DQA1*0509-DQB1*0512 |
| 2456 |  |  | HLA-DQA1*0509-DQB1*0513 |
| 2457 |  |  | HLA-DQA1*0509-DQB1*0514 |
| 2458 |  |  | HLA-DQA1*0509-DQB1*0601 |
| 2459 |  |  | HLA-DQA1*0509-DQB1*0602 |
| 2460 |  |  | HLA-DQA1*0509-DQB1*0603 |
| 2461 |  |  | HLA-DQA1*0509-DQB1*0604 |
| 2462 |  |  | HLA-DQA1*0509-DQB1*0607 |
| 2463 |  |  | HLA-DQA1*0509-DQB1*0608 |
| 2464 |  |  | HLA-DQA1*0509-DQB1*0609 |
| 2465 |  |  | HLA-DQA1*0509-DQB1*0610 |
| 2466 |  |  | HLA-DQA1*0509-DQB1*0611 |
| 2467 |  |  | HLA-DQA1*0509-DQB1*0612 |
| 2468 |  |  | HLA-DQA1*0509-DQB1*0614 |
| 2469 |  |  | HLA-DQA1*0509-DQB1*0615 |
| 2470 |  |  | HLA-DQA1*0509-DQB1*0616 |
| 2471 |  |  | HLA-DQA1*0509-DQB1*0617 |
| 2472 |  |  | HLA-DQA1*0509-DQB1*0618 |
| 2473 |  |  | HLA-DQA1*0509-DQB1*0619 |
| 2474 |  |  | HLA-DQA1*0509-DQB1*0621 |
| 2475 |  |  | HLA-DQA1*0509-DQB1*0622 |
| 2476 |  |  | HLA-DQA1*0509-DQB1*0623 |
| 2477 |  |  | HLA-DQA1*0509-DQB1*0624 |
| 2478 |  |  | HLA-DQA1*0509-DQB1*0625 |
| 2479 |  |  | HLA-DQA1*0509-DQB1*0627 |
| 2480 |  |  | HLA-DQA1*0509-DQB1*0628 |
| 2481 |  |  | HLA-DQA1*0509-DQB1*0629 |
| 2482 |  |  | HLA-DQA1*0509-DQB1*0630 |
| 2483 |  |  | HLA-DQA1*0509-DQB1*0631 |
| 2484 |  |  | HLA-DQA1*0509-DQB1*0632 |
| 2485 |  |  | HLA-DQA1*0509-DQB1*0633 |
| 2486 |  |  | HLA-DQA1*0509-DQB1*0634 |
| 2487 |  |  | HLA-DQA1*0509-DQB1*0635 |
| 2488 |  |  | HLA-DQA1*0509-DQB1*0636 |
| 2489 |  |  | HLA-DQA1*0509-DQB1*0637 |
| 2490 |  |  | HLA-DQA1*0509-DQB1*0638 |
| 2491 |  |  | HLA-DQA1*0509-DQB1*0639 |
| 2492 |  |  | HLA-DQA1*0509-DQB1*0640 |
| 2493 |  |  | HLA-DQA1*0509-DQB1*0641 |
| 2494 |  |  | HLA-DQA1*0509-DQB1*0642 |
| 2495 |  |  | HLA-DQA1*0509-DQB1*0643 |
| 2496 |  |  | HLA-DQA1*0509-DQB1*0644 |
| 2497 |  |  | HLA-DQA1*0510-DQB1*0201 |
| 2498 |  |  | HLA-DQA1*0510-DQB1*0202 |
| 2499 |  |  | HLA-DQA1*0510-DQB1*0203 |
| 2500 |  |  | HLA-DQA1*0510-DQB1*0204 |
| 2501 |  |  | HLA-DQA1*0510-DQB1*0205 |
| 2502 |  |  | HLA-DQA1*0510-DQB1*0206 |
| 2503 |  |  | HLA-DQA1*0510-DQB1*0301 |
| 2504 |  |  | HLA-DQA1*0510-DQB1*0302 |
| 2505 |  |  | HLA-DQA1*0510-DQB1*0303 |
| 2506 |  |  | HLA-DQA1*0510-DQB1*0304 |
| 2507 |  |  | HLA-DQA1*0510-DQB1*0305 |
| 2508 |  |  | HLA-DQA1*0510-DQB1*0306 |
| 2509 |  |  | HLA-DQA1*0510-DQB1*0307 |
| 2510 |  |  | HLA-DQA1*0510-DQB1*0308 |
| 2511 |  |  | HLA-DQA1*0510-DQB1*0309 |
| 2512 |  |  | HLA-DQA1*0510-DQB1*0310 |
| 2513 |  |  | HLA-DQA1*0510-DQB1*0311 |
| 2514 |  |  | HLA-DQA1*0510-DQB1*0312 |
| 2515 |  |  | HLA-DQA1*0510-DQB1*0313 |
| 2516 |  |  | HLA-DQA1*0510-DQB1*0314 |
| 2517 |  |  | HLA-DQA1*0510-DQB1*0315 |
| 2518 |  |  | HLA-DQA1*0510-DQB1*0316 |
| 2519 |  |  | HLA-DQA1*0510-DQB1*0317 |
| 2520 |  |  | HLA-DQA1*0510-DQB1*0318 |
| 2521 |  |  | HLA-DQA1*0510-DQB1*0319 |
| 2522 |  |  | HLA-DQA1*0510-DQB1*0320 |
| 2523 |  |  | HLA-DQA1*0510-DQB1*0321 |
| 2524 |  |  | HLA-DQA1*0510-DQB1*0322 |
| 2525 |  |  | HLA-DQA1*0510-DQB1*0323 |
| 2526 |  |  | HLA-DQA1*0510-DQB1*0324 |
| 2527 |  |  | HLA-DQA1*0510-DQB1*0325 |
| 2528 |  |  | HLA-DQA1*0510-DQB1*0326 |
| 2529 |  |  | HLA-DQA1*0510-DQB1*0327 |
| 2530 |  |  | HLA-DQA1*0510-DQB1*0328 |
| 2531 |  |  | HLA-DQA1*0510-DQB1*0329 |
| 2532 |  |  | HLA-DQA1*0510-DQB1*0330 |
| 2533 |  |  | HLA-DQA1*0510-DQB1*0331 |
| 2534 |  |  | HLA-DQA1*0510-DQB1*0332 |
| 2535 |  |  | HLA-DQA1*0510-DQB1*0333 |
| 2536 |  |  | HLA-DQA1*0510-DQB1*0334 |
| 2537 |  |  | HLA-DQA1*0510-DQB1*0335 |
| 2538 |  |  | HLA-DQA1*0510-DQB1*0336 |
| 2539 |  |  | HLA-DQA1*0510-DQB1*0337 |
| 2540 |  |  | HLA-DQA1*0510-DQB1*0338 |
| 2541 |  |  | HLA-DQA1*0510-DQB1*0401 |
| 2542 |  |  | HLA-DQA1*0510-DQB1*0402 |
| 2543 |  |  | HLA-DQA1*0510-DQB1*0403 |
| 2544 |  |  | HLA-DQA1*0510-DQB1*0404 |
| 2545 |  |  | HLA-DQA1*0510-DQB1*0405 |
| 2546 |  |  | HLA-DQA1*0510-DQB1*0406 |
| 2547 |  |  | HLA-DQA1*0510-DQB1*0407 |
| 2548 |  |  | HLA-DQA1*0510-DQB1*0408 |
| 2549 |  |  | HLA-DQA1*0510-DQB1*0501 |
| 2550 |  |  | HLA-DQA1*0510-DQB1*0502 |
| 2551 |  |  | HLA-DQA1*0510-DQB1*0503 |
| 2552 |  |  | HLA-DQA1*0510-DQB1*0505 |
| 2553 |  |  | HLA-DQA1*0510-DQB1*0506 |
| 2554 |  |  | HLA-DQA1*0510-DQB1*0507 |
| 2555 |  |  | HLA-DQA1*0510-DQB1*0508 |
| 2556 |  |  | HLA-DQA1*0510-DQB1*0509 |
| 2557 |  |  | HLA-DQA1*0510-DQB1*0510 |
| 2558 |  |  | HLA-DQA1*0510-DQB1*0511 |
| 2559 |  |  | HLA-DQA1*0510-DQB1*0512 |
| 2560 |  |  | HLA-DQA1*0510-DQB1*0513 |
| 2561 |  |  | HLA-DQA1*0510-DQB1*0514 |
| 2562 |  |  | HLA-DQA1*0510-DQB1*0601 |
| 2563 |  |  | HLA-DQA1*0510-DQB1*0602 |
| 2564 |  |  | HLA-DQA1*0510-DQB1*0603 |
| 2565 |  |  | HLA-DQA1*0510-DQB1*0604 |
| 2566 |  |  | HLA-DQA1*0510-DQB1*0607 |
| 2567 |  |  | HLA-DQA1*0510-DQB1*0608 |
| 2568 |  |  | HLA-DQA1*0510-DQB1*0609 |
| 2569 |  |  | HLA-DQA1*0510-DQB1*0610 |
| 2570 |  |  | HLA-DQA1*0510-DQB1*0611 |
| 2571 |  |  | HLA-DQA1*0510-DQB1*0612 |
| 2572 |  |  | HLA-DQA1*0510-DQB1*0614 |
| 2573 |  |  | HLA-DQA1*0510-DQB1*0615 |
| 2574 |  |  | HLA-DQA1*0510-DQB1*0616 |
| 2575 |  |  | HLA-DQA1*0510-DQB1*0617 |
| 2576 |  |  | HLA-DQA1*0510-DQB1*0618 |
| 2577 |  |  | HLA-DQA1*0510-DQB1*0619 |
| 2578 |  |  | HLA-DQA1*0510-DQB1*0621 |
| 2579 |  |  | HLA-DQA1*0510-DQB1*0622 |
| 2580 |  |  | HLA-DQA1*0510-DQB1*0623 |
| 2581 |  |  | HLA-DQA1*0510-DQB1*0624 |
| 2582 |  |  | HLA-DQA1*0510-DQB1*0625 |
| 2583 |  |  | HLA-DQA1*0510-DQB1*0627 |
| 2584 |  |  | HLA-DQA1*0510-DQB1*0628 |
| 2585 |  |  | HLA-DQA1*0510-DQB1*0629 |
| 2586 |  |  | HLA-DQA1*0510-DQB1*0630 |
| 2587 |  |  | HLA-DQA1*0510-DQB1*0631 |
| 2588 |  |  | HLA-DQA1*0510-DQB1*0632 |
| 2589 |  |  | HLA-DQA1*0510-DQB1*0633 |
| 2590 |  |  | HLA-DQA1*0510-DQB1*0634 |
| 2591 |  |  | HLA-DQA1*0510-DQB1*0635 |
| 2592 |  |  | HLA-DQA1*0510-DQB1*0636 |
| 2593 |  |  | HLA-DQA1*0510-DQB1*0637 |
| 2594 |  |  | HLA-DQA1*0510-DQB1*0638 |
| 2595 |  |  | HLA-DQA1*0510-DQB1*0639 |
| 2596 |  |  | HLA-DQA1*0510-DQB1*0640 |
| 2597 |  |  | HLA-DQA1*0510-DQB1*0641 |
| 2598 |  |  | HLA-DQA1*0510-DQB1*0642 |
| 2599 |  |  | HLA-DQA1*0510-DQB1*0643 |
| 2600 |  |  | HLA-DQA1*0510-DQB1*0644 |
| 2601 |  |  | HLA-DQA1*0511-DQB1*0201 |
| 2602 |  |  | HLA-DQA1*0511-DQB1*0202 |
| 2603 |  |  | HLA-DQA1*0511-DQB1*0203 |
| 2604 |  |  | HLA-DQA1*0511-DQB1*0204 |
| 2605 |  |  | HLA-DQA1*0511-DQB1*0205 |
| 2606 |  |  | HLA-DQA1*0511-DQB1*0206 |
| 2607 |  |  | HLA-DQA1*0511-DQB1*0301 |
| 2608 |  |  | HLA-DQA1*0511-DQB1*0302 |
| 2609 |  |  | HLA-DQA1*0511-DQB1*0303 |
| 2610 |  |  | HLA-DQA1*0511-DQB1*0304 |
| 2611 |  |  | HLA-DQA1*0511-DQB1*0305 |
| 2612 |  |  | HLA-DQA1*0511-DQB1*0306 |
| 2613 |  |  | HLA-DQA1*0511-DQB1*0307 |
| 2614 |  |  | HLA-DQA1*0511-DQB1*0308 |
| 2615 |  |  | HLA-DQA1*0511-DQB1*0309 |
| 2616 |  |  | HLA-DQA1*0511-DQB1*0310 |
| 2617 |  |  | HLA-DQA1*0511-DQB1*0311 |
| 2618 |  |  | HLA-DQA1*0511-DQB1*0312 |
| 2619 |  |  | HLA-DQA1*0511-DQB1*0313 |
| 2620 |  |  | HLA-DQA1*0511-DQB1*0314 |
| 2621 |  |  | HLA-DQA1*0511-DQB1*0315 |
| 2622 |  |  | HLA-DQA1*0511-DQB1*0316 |
| 2623 |  |  | HLA-DQA1*0511-DQB1*0317 |
| 2624 |  |  | HLA-DQA1*0511-DQB1*0318 |
| 2625 |  |  | HLA-DQA1*0511-DQB1*0319 |
| 2626 |  |  | HLA-DQA1*0511-DQB1*0320 |
| 2627 |  |  | HLA-DQA1*0511-DQB1*0321 |
| 2628 |  |  | HLA-DQA1*0511-DQB1*0322 |
| 2629 |  |  | HLA-DQA1*0511-DQB1*0323 |
| 2630 |  |  | HLA-DQA1*0511-DQB1*0324 |
| 2631 |  |  | HLA-DQA1*0511-DQB1*0325 |
| 2632 |  |  | HLA-DQA1*0511-DQB1*0326 |
| 2633 |  |  | HLA-DQA1*0511-DQB1*0327 |
| 2634 |  |  | HLA-DQA1*0511-DQB1*0328 |
| 2635 |  |  | HLA-DQA1*0511-DQB1*0329 |
| 2636 |  |  | HLA-DQA1*0511-DQB1*0330 |
| 2637 |  |  | HLA-DQA1*0511-DQB1*0331 |
| 2638 |  |  | HLA-DQA1*0511-DQB1*0332 |
| 2639 |  |  | HLA-DQA1*0511-DQB1*0333 |
| 2640 |  |  | HLA-DQA1*0511-DQB1*0334 |
| 2641 |  |  | HLA-DQA1*0511-DQB1*0335 |
| 2642 |  |  | HLA-DQA1*0511-DQB1*0336 |
| 2643 |  |  | HLA-DQA1*0511-DQB1*0337 |
| 2644 |  |  | HLA-DQA1*0511-DQB1*0338 |
| 2645 |  |  | HLA-DQA1*0511-DQB1*0401 |
| 2646 |  |  | HLA-DQA1*0511-DQB1*0402 |
| 2647 |  |  | HLA-DQA1*0511-DQB1*0403 |
| 2648 |  |  | HLA-DQA1*0511-DQB1*0404 |
| 2649 |  |  | HLA-DQA1*0511-DQB1*0405 |
| 2650 |  |  | HLA-DQA1*0511-DQB1*0406 |
| 2651 |  |  | HLA-DQA1*0511-DQB1*0407 |
| 2652 |  |  | HLA-DQA1*0511-DQB1*0408 |
| 2653 |  |  | HLA-DQA1*0511-DQB1*0501 |
| 2654 |  |  | HLA-DQA1*0511-DQB1*0502 |
| 2655 |  |  | HLA-DQA1*0511-DQB1*0503 |
| 2656 |  |  | HLA-DQA1*0511-DQB1*0505 |
| 2657 |  |  | HLA-DQA1*0511-DQB1*0506 |
| 2658 |  |  | HLA-DQA1*0511-DQB1*0507 |
| 2659 |  |  | HLA-DQA1*0511-DQB1*0508 |
| 2660 |  |  | HLA-DQA1*0511-DQB1*0509 |
| 2661 |  |  | HLA-DQA1*0511-DQB1*0510 |
| 2662 |  |  | HLA-DQA1*0511-DQB1*0511 |
| 2663 |  |  | HLA-DQA1*0511-DQB1*0512 |
| 2664 |  |  | HLA-DQA1*0511-DQB1*0513 |
| 2665 |  |  | HLA-DQA1*0511-DQB1*0514 |
| 2666 |  |  | HLA-DQA1*0511-DQB1*0601 |
| 2667 |  |  | HLA-DQA1*0511-DQB1*0602 |
| 2668 |  |  | HLA-DQA1*0511-DQB1*0603 |
| 2669 |  |  | HLA-DQA1*0511-DQB1*0604 |
| 2670 |  |  | HLA-DQA1*0511-DQB1*0607 |
| 2671 |  |  | HLA-DQA1*0511-DQB1*0608 |
| 2672 |  |  | HLA-DQA1*0511-DQB1*0609 |
| 2673 |  |  | HLA-DQA1*0511-DQB1*0610 |
| 2674 |  |  | HLA-DQA1*0511-DQB1*0611 |
| 2675 |  |  | HLA-DQA1*0511-DQB1*0612 |
| 2676 |  |  | HLA-DQA1*0511-DQB1*0614 |
| 2677 |  |  | HLA-DQA1*0511-DQB1*0615 |
| 2678 |  |  | HLA-DQA1*0511-DQB1*0616 |
| 2679 |  |  | HLA-DQA1*0511-DQB1*0617 |
| 2680 |  |  | HLA-DQA1*0511-DQB1*0618 |
| 2681 |  |  | HLA-DQA1*0511-DQB1*0619 |
| 2682 |  |  | HLA-DQA1*0511-DQB1*0621 |
| 2683 |  |  | HLA-DQA1*0511-DQB1*0622 |
| 2684 |  |  | HLA-DQA1*0511-DQB1*0623 |
| 2685 |  |  | HLA-DQA1*0511-DQB1*0624 |
| 2686 |  |  | HLA-DQA1*0511-DQB1*0625 |
| 2687 |  |  | HLA-DQA1*0511-DQB1*0627 |
| 2688 |  |  | HLA-DQA1*0511-DQB1*0628 |
| 2689 |  |  | HLA-DQA1*0511-DQB1*0629 |
| 2690 |  |  | HLA-DQA1*0511-DQB1*0630 |
| 2691 |  |  | HLA-DQA1*0511-DQB1*0631 |
| 2692 |  |  | HLA-DQA1*0511-DQB1*0632 |
| 2693 |  |  | HLA-DQA1*0511-DQB1*0633 |
| 2694 |  |  | HLA-DQA1*0511-DQB1*0634 |
| 2695 |  |  | HLA-DQA1*0511-DQB1*0635 |
| 2696 |  |  | HLA-DQA1*0511-DQB1*0636 |
| 2697 |  |  | HLA-DQA1*0511-DQB1*0637 |
| 2698 |  |  | HLA-DQA1*0511-DQB1*0638 |
| 2699 |  |  | HLA-DQA1*0511-DQB1*0639 |
| 2700 |  |  | HLA-DQA1*0511-DQB1*0640 |
| 2701 |  |  | HLA-DQA1*0511-DQB1*0641 |
| 2702 |  |  | HLA-DQA1*0511-DQB1*0642 |
| 2703 |  |  | HLA-DQA1*0511-DQB1*0643 |
| 2704 |  |  | HLA-DQA1*0511-DQB1*0644 |
| 2705 |  |  | HLA-DQA1*0601-DQB1*0201 |
| 2706 |  |  | HLA-DQA1*0601-DQB1*0202 |
| 2707 |  |  | HLA-DQA1*0601-DQB1*0203 |
| 2708 |  |  | HLA-DQA1*0601-DQB1*0204 |
| 2709 |  |  | HLA-DQA1*0601-DQB1*0205 |
| 2710 |  |  | HLA-DQA1*0601-DQB1*0206 |
| 2711 |  |  | HLA-DQA1*0601-DQB1*0301 |
| 2712 |  |  | HLA-DQA1*0601-DQB1*0302 |
| 2713 |  |  | HLA-DQA1*0601-DQB1*0303 |
| 2714 |  |  | HLA-DQA1*0601-DQB1*0304 |
| 2715 |  |  | HLA-DQA1*0601-DQB1*0305 |
| 2716 |  |  | HLA-DQA1*0601-DQB1*0306 |
| 2717 |  |  | HLA-DQA1*0601-DQB1*0307 |
| 2718 |  |  | HLA-DQA1*0601-DQB1*0308 |
| 2719 |  |  | HLA-DQA1*0601-DQB1*0309 |
| 2720 |  |  | HLA-DQA1*0601-DQB1*0310 |
| 2721 |  |  | HLA-DQA1*0601-DQB1*0311 |
| 2722 |  |  | HLA-DQA1*0601-DQB1*0312 |
| 2723 |  |  | HLA-DQA1*0601-DQB1*0313 |
| 2724 |  |  | HLA-DQA1*0601-DQB1*0314 |
| 2725 |  |  | HLA-DQA1*0601-DQB1*0315 |
| 2726 |  |  | HLA-DQA1*0601-DQB1*0316 |
| 2727 |  |  | HLA-DQA1*0601-DQB1*0317 |
| 2728 |  |  | HLA-DQA1*0601-DQB1*0318 |
| 2729 |  |  | HLA-DQA1*0601-DQB1*0319 |
| 2730 |  |  | HLA-DQA1*0601-DQB1*0320 |
| 2731 |  |  | HLA-DQA1*0601-DQB1*0321 |
| 2732 |  |  | HLA-DQA1*0601-DQB1*0322 |
| 2733 |  |  | HLA-DQA1*0601-DQB1*0323 |
| 2734 |  |  | HLA-DQA1*0601-DQB1*0324 |
| 2735 |  |  | HLA-DQA1*0601-DQB1*0325 |
| 2736 |  |  | HLA-DQA1*0601-DQB1*0326 |
| 2737 |  |  | HLA-DQA1*0601-DQB1*0327 |
| 2738 |  |  | HLA-DQA1*0601-DQB1*0328 |
| 2739 |  |  | HLA-DQA1*0601-DQB1*0329 |
| 2740 |  |  | HLA-DQA1*0601-DQB1*0330 |
| 2741 |  |  | HLA-DQA1*0601-DQB1*0331 |
| 2742 |  |  | HLA-DQA1*0601-DQB1*0332 |
| 2743 |  |  | HLA-DQA1*0601-DQB1*0333 |
| 2744 |  |  | HLA-DQA1*0601-DQB1*0334 |
| 2745 |  |  | HLA-DQA1*0601-DQB1*0335 |
| 2746 |  |  | HLA-DQA1*0601-DQB1*0336 |
| 2747 |  |  | HLA-DQA1*0601-DQB1*0337 |
| 2748 |  |  | HLA-DQA1*0601-DQB1*0338 |
| 2749 |  |  | HLA-DQA1*0601-DQB1*0401 |
| 2750 |  |  | HLA-DQA1*0601-DQB1*0402 |
| 2751 |  |  | HLA-DQA1*0601-DQB1*0403 |
| 2752 |  |  | HLA-DQA1*0601-DQB1*0404 |
| 2753 |  |  | HLA-DQA1*0601-DQB1*0405 |
| 2754 |  |  | HLA-DQA1*0601-DQB1*0406 |
| 2755 |  |  | HLA-DQA1*0601-DQB1*0407 |
| 2756 |  |  | HLA-DQA1*0601-DQB1*0408 |
| 2757 |  |  | HLA-DQA1*0601-DQB1*0501 |
| 2758 |  |  | HLA-DQA1*0601-DQB1*0502 |
| 2759 |  |  | HLA-DQA1*0601-DQB1*0503 |
| 2760 |  |  | HLA-DQA1*0601-DQB1*0505 |
| 2761 |  |  | HLA-DQA1*0601-DQB1*0506 |
| 2762 |  |  | HLA-DQA1*0601-DQB1*0507 |
| 2763 |  |  | HLA-DQA1*0601-DQB1*0508 |
| 2764 |  |  | HLA-DQA1*0601-DQB1*0509 |
| 2765 |  |  | HLA-DQA1*0601-DQB1*0510 |
| 2766 |  |  | HLA-DQA1*0601-DQB1*0511 |
| 2767 |  |  | HLA-DQA1*0601-DQB1*0512 |
| 2768 |  |  | HLA-DQA1*0601-DQB1*0513 |
| 2769 |  |  | HLA-DQA1*0601-DQB1*0514 |
| 2770 |  |  | HLA-DQA1*0601-DQB1*0601 |
| 2771 |  |  | HLA-DQA1*0601-DQB1*0602 |
| 2772 |  |  | HLA-DQA1*0601-DQB1*0603 |
| 2773 |  |  | HLA-DQA1*0601-DQB1*0604 |
| 2774 |  |  | HLA-DQA1*0601-DQB1*0607 |
| 2775 |  |  | HLA-DQA1*0601-DQB1*0608 |
| 2776 |  |  | HLA-DQA1*0601-DQB1*0609 |
| 2777 |  |  | HLA-DQA1*0601-DQB1*0610 |
| 2778 |  |  | HLA-DQA1*0601-DQB1*0611 |
| 2779 |  |  | HLA-DQA1*0601-DQB1*0612 |
| 2780 |  |  | HLA-DQA1*0601-DQB1*0614 |
| 2781 |  |  | HLA-DQA1*0601-DQB1*0615 |
| 2782 |  |  | HLA-DQA1*0601-DQB1*0616 |
| 2783 |  |  | HLA-DQA1*0601-DQB1*0617 |
| 2784 |  |  | HLA-DQA1*0601-DQB1*0618 |
| 2785 |  |  | HLA-DQA1*0601-DQB1*0619 |
| 2786 |  |  | HLA-DQA1*0601-DQB1*0621 |
| 2787 |  |  | HLA-DQA1*0601-DQB1*0622 |
| 2788 |  |  | HLA-DQA1*0601-DQB1*0623 |
| 2789 |  |  | HLA-DQA1*0601-DQB1*0624 |
| 2790 |  |  | HLA-DQA1*0601-DQB1*0625 |
| 2791 |  |  | HLA-DQA1*0601-DQB1*0627 |
| 2792 |  |  | HLA-DQA1*0601-DQB1*0628 |
| 2793 |  |  | HLA-DQA1*0601-DQB1*0629 |
| 2794 |  |  | HLA-DQA1*0601-DQB1*0630 |
| 2795 |  |  | HLA-DQA1*0601-DQB1*0631 |
| 2796 |  |  | HLA-DQA1*0601-DQB1*0632 |
| 2797 |  |  | HLA-DQA1*0601-DQB1*0633 |
| 2798 |  |  | HLA-DQA1*0601-DQB1*0634 |
| 2799 |  |  | HLA-DQA1*0601-DQB1*0635 |
| 2800 |  |  | HLA-DQA1*0601-DQB1*0636 |
| 2801 |  |  | HLA-DQA1*0601-DQB1*0637 |
| 2802 |  |  | HLA-DQA1*0601-DQB1*0638 |
| 2803 |  |  | HLA-DQA1*0601-DQB1*0639 |
| 2804 |  |  | HLA-DQA1*0601-DQB1*0640 |
| 2805 |  |  | HLA-DQA1*0601-DQB1*0641 |
| 2806 |  |  | HLA-DQA1*0601-DQB1*0642 |
| 2807 |  |  | HLA-DQA1*0601-DQB1*0643 |
| 2808 |  |  | HLA-DQA1*0601-DQB1*0644 |
| 2809 |  |  | HLA-DQA1*0602-DQB1*0201 |
| 2810 |  |  | HLA-DQA1*0602-DQB1*0202 |
| 2811 |  |  | HLA-DQA1*0602-DQB1*0203 |
| 2812 |  |  | HLA-DQA1*0602-DQB1*0204 |
| 2813 |  |  | HLA-DQA1*0602-DQB1*0205 |
| 2814 |  |  | HLA-DQA1*0602-DQB1*0206 |
| 2815 |  |  | HLA-DQA1*0602-DQB1*0301 |
| 2816 |  |  | HLA-DQA1*0602-DQB1*0302 |
| 2817 |  |  | HLA-DQA1*0602-DQB1*0303 |
| 2818 |  |  | HLA-DQA1*0602-DQB1*0304 |
| 2819 |  |  | HLA-DQA1*0602-DQB1*0305 |
| 2820 |  |  | HLA-DQA1*0602-DQB1*0306 |
| 2821 |  |  | HLA-DQA1*0602-DQB1*0307 |
| 2822 |  |  | HLA-DQA1*0602-DQB1*0308 |
| 2823 |  |  | HLA-DQA1*0602-DQB1*0309 |
| 2824 |  |  | HLA-DQA1*0602-DQB1*0310 |
| 2825 |  |  | HLA-DQA1*0602-DQB1*0311 |
| 2826 |  |  | HLA-DQA1*0602-DQB1*0312 |
| 2827 |  |  | HLA-DQA1*0602-DQB1*0313 |
| 2828 |  |  | HLA-DQA1*0602-DQB1*0314 |
| 2829 |  |  | HLA-DQA1*0602-DQB1*0315 |
| 2830 |  |  | HLA-DQA1*0602-DQB1*0316 |
| 2831 |  |  | HLA-DQA1*0602-DQB1*0317 |
| 2832 |  |  | HLA-DQA1*0602-DQB1*0318 |
| 2833 |  |  | HLA-DQA1*0602-DQB1*0319 |
| 2834 |  |  | HLA-DQA1*0602-DQB1*0320 |
| 2835 |  |  | HLA-DQA1*0602-DQB1*0321 |
| 2836 |  |  | HLA-DQA1*0602-DQB1*0322 |
| 2837 |  |  | HLA-DQA1*0602-DQB1*0323 |
| 2838 |  |  | HLA-DQA1*0602-DQB1*0324 |
| 2839 |  |  | HLA-DQA1*0602-DQB1*0325 |
| 2840 |  |  | HLA-DQA1*0602-DQB1*0326 |
| 2841 |  |  | HLA-DQA1*0602-DQB1*0327 |
| 2842 |  |  | HLA-DQA1*0602-DQB1*0328 |
| 2843 |  |  | HLA-DQA1*0602-DQB1*0329 |
| 2844 |  |  | HLA-DQA1*0602-DQB1*0330 |
| 2845 |  |  | HLA-DQA1*0602-DQB1*0331 |
| 2846 |  |  | HLA-DQA1*0602-DQB1*0332 |
| 2847 |  |  | HLA-DQA1*0602-DQB1*0333 |
| 2848 |  |  | HLA-DQA1*0602-DQB1*0334 |
| 2849 |  |  | HLA-DQA1*0602-DQB1*0335 |
| 2850 |  |  | HLA-DQA1*0602-DQB1*0336 |
| 2851 |  |  | HLA-DQA1*0602-DQB1*0337 |
| 2852 |  |  | HLA-DQA1*0602-DQB1*0338 |
| 2853 |  |  | HLA-DQA1*0602-DQB1*0401 |
| 2854 |  |  | HLA-DQA1*0602-DQB1*0402 |
| 2855 |  |  | HLA-DQA1*0602-DQB1*0403 |
| 2856 |  |  | HLA-DQA1*0602-DQB1*0404 |
| 2857 |  |  | HLA-DQA1*0602-DQB1*0405 |
| 2858 |  |  | HLA-DQA1*0602-DQB1*0406 |
| 2859 |  |  | HLA-DQA1*0602-DQB1*0407 |
| 2860 |  |  | HLA-DQA1*0602-DQB1*0408 |
| 2861 |  |  | HLA-DQA1*0602-DQB1*0501 |
| 2862 |  |  | HLA-DQA1*0602-DQB1*0502 |
| 2863 |  |  | HLA-DQA1*0602-DQB1*0503 |
| 2864 |  |  | HLA-DQA1*0602-DQB1*0505 |
| 2865 |  |  | HLA-DQA1*0602-DQB1*0506 |
| 2866 |  |  | HLA-DQA1*0602-DQB1*0507 |
| 2867 |  |  | HLA-DQA1*0602-DQB1*0508 |
| 2868 |  |  | HLA-DQA1*0602-DQB1*0509 |
| 2869 |  |  | HLA-DQA1*0602-DQB1*0510 |
| 2870 |  |  | HLA-DQA1*0602-DQB1*0511 |
| 2871 |  |  | HLA-DQA1*0602-DQB1*0512 |
| 2872 |  |  | HLA-DQA1*0602-DQB1*0513 |
| 2873 |  |  | HLA-DQA1*0602-DQB1*0514 |
| 2874 |  |  | HLA-DQA1*0602-DQB1*0601 |
| 2875 |  |  | HLA-DQA1*0602-DQB1*0602 |
| 2876 |  |  | HLA-DQA1*0602-DQB1*0603 |
| 2877 |  |  | HLA-DQA1*0602-DQB1*0604 |
| 2878 |  |  | HLA-DQA1*0602-DQB1*0607 |
| 2879 |  |  | HLA-DQA1*0602-DQB1*0608 |
| 2880 |  |  | HLA-DQA1*0602-DQB1*0609 |
| 2881 |  |  | HLA-DQA1*0602-DQB1*0610 |
| 2882 |  |  | HLA-DQA1*0602-DQB1*0611 |
| 2883 |  |  | HLA-DQA1*0602-DQB1*0612 |
| 2884 |  |  | HLA-DQA1*0602-DQB1*0614 |
| 2885 |  |  | HLA-DQA1*0602-DQB1*0615 |
| 2886 |  |  | HLA-DQA1*0602-DQB1*0616 |
| 2887 |  |  | HLA-DQA1*0602-DQB1*0617 |
| 2888 |  |  | HLA-DQA1*0602-DQB1*0618 |
| 2889 |  |  | HLA-DQA1*0602-DQB1*0619 |
| 2890 |  |  | HLA-DQA1*0602-DQB1*0621 |
| 2891 |  |  | HLA-DQA1*0602-DQB1*0622 |
| 2892 |  |  | HLA-DQA1*0602-DQB1*0623 |
| 2893 |  |  | HLA-DQA1*0602-DQB1*0624 |
| 2894 |  |  | HLA-DQA1*0602-DQB1*0625 |
| 2895 |  |  | HLA-DQA1*0602-DQB1*0627 |
| 2896 |  |  | HLA-DQA1*0602-DQB1*0628 |
| 2897 |  |  | HLA-DQA1*0602-DQB1*0629 |
| 2898 |  |  | HLA-DQA1*0602-DQB1*0630 |
| 2899 |  |  | HLA-DQA1*0602-DQB1*0631 |
| 2900 |  |  | HLA-DQA1*0602-DQB1*0632 |
| 2901 |  |  | HLA-DQA1*0602-DQB1*0633 |
| 2902 |  |  | HLA-DQA1*0602-DQB1*0634 |
| 2903 |  |  | HLA-DQA1*0602-DQB1*0635 |
| 2904 |  |  | HLA-DQA1*0602-DQB1*0636 |
| 2905 |  |  | HLA-DQA1*0602-DQB1*0637 |
| 2906 |  |  | HLA-DQA1*0602-DQB1*0638 |
| 2907 |  |  | HLA-DQA1*0602-DQB1*0639 |
| 2908 |  |  | HLA-DQA1*0602-DQB1*0640 |
| 2909 |  |  | HLA-DQA1*0602-DQB1*0641 |
| 2910 |  |  | HLA-DQA1*0602-DQB1*0642 |
| 2911 |  |  | HLA-DQA1*0602-DQB1*0643 |
| 2912 |  |  | HLA-DQA1*0602-DQB1*0644 |
