# Supplementary material for: Prediction of immunogenicity of Rh antigens using in silico analysis of binding to human leukocyte antigen peptide, Basic/Translational Research
Source: PLoS One. 2025 Oct 27;20(10):e0334851. doi: 10.1371/journal.pone.0334851 (PMC12558515; doi:10.1371/journal.pone.0334851)
Supplement: S2 Table — The serology of HLA-DRB1 in RhD antigens showed that DR1 had VTAISGSSL at the 250th position of the hotspot. In contrast, DR17 (DR3) had ISVDAVLGK at the 125th position of the hotspot. DR4, DR7, DR8, and DR10 had a high frequency of YVHSAVLAG core amino acids at the 269th amino acid start position of the hotspot. Furthermore, DR12 and DR15 had high frequencies of LISVGGAKY and LSQFPSGKV core amino acids at the 303rd and 98th positions of the hotspot, respectively. RhCE in HLA-DRB1 showed a different hotspot start position in the RhD antigens. (DOCX) [file pone.0334851.s004.docx]

**S2 Table. HLA-DRB1 hotspot (SB) regions and frequencies in the four ethnic groups.** The serology of HLA-DRB1 in RhD antigens showed that DR1 had VTAISGSSL at the 250^th^ position of the hotspot. In contrast, DR17 (DR3) had ISVDAVLGK at the 125^th^ position of the hotspot. DR4, DR7, DR8, and DR10 had a high frequency of YVHSAVLAG core amino acids at the 269^th^ amino acid start position of the hotspot. Furthermore, DR12 and DR15 had high frequencies of LISVGGAKY and LSQFPSGKV core amino acids at the 303^rd^ and 98^th^ positions of the hotspot, respectively. RhCE in HLA-DRB1 showed a different hotspot start position in the RhD antigens.

| HLA-DRB1 Allele | Caucasian (n = 1070) | African American (n = 2411) | Hispanic (n = 1999) | Asian (n = 1772) | RHD*01.01/ RHD*01W.2/ RHD*01W.3 | | RHD*01W.1 | | RHCE*01 | |
| --- | --- | --- | --- | --- | --- | --- | --- | --- | --- | --- |
|  |  |  |  |  | Amino acid start position of hotspot | Core amino acids | Amino acid start position of hotspot | Core amino acids | Amino acid start position of hotspot | Core amino acids |
| 01:01 | 0.0865 | 0.026 | 0.039 | 0.0274 | – | – | – | – | – | – |
| 01:02 | 0.0202 | 0.0399 | 0.033 | 0.0003 | 250 | VTAISGSSL | 250 | VTAISGSSL | 250 | VTAISGSSL |
| 01:03 | 0.0192 | 0.0023 | 0.006 | 0.0003 | – | – | – | – | 390 | LKIWKAPHV |
| 03:01 | 0.1039 | 0.0707 | 0.073 | 0.0537 | 125 | ISVDAVLGK | 125 | ISVDAVLGK | – | – |
| 03:02 | 0.001 | 0.0653 | 0.008 | 0 | – | – | – | – | – | – |
| 03:05 | 0.001 | 0.0002 | 3E-04 | 0 | – | – | – | – | 161 | FNTDYHMNL |
| 03:06 |  | 0.0002 | 0 | 0 | 125 | ISVDAVLGK | 125 | ISVDAVLGK | – | – |
| 04:01 | 0.1039 | 0.0229 | 0.015 | 0.0091 | 269 | YVHSAVLAG | – | – | 269 | YVHSAVLAG |
| 04:02 | 0.0039 | 0.0004 | 0.02 | 0.0037 | 390 | LKIWKAPHE | 390 | LKIWKAPHE | 130 | VLGKVNLAQ |
| 04:03 | 0.0048 | 0.0023 | 0.019 | 0.0345 | – | – | – | – | – | – |
| 04:04 | 0.0471 | 0.0069 | 0.055 | 0.0091 | – | – | – | – | – | – |
| 04:05 | 0.0019 | 0.0096 | 0.019 | 0.058 | – | – | – | – | – | – |
| 04:06 | 0.001 | 0.0006 | 0.002 | 0.0206 | – | – | – | – | – | – |
| 04:07 | 0.0154 | 0.004 | 0.064 | 0.0014 | 269 | YVHSAVLAG | 269 | YGHSAVLAG | 269 | YVHSAVLAG |
| 04:08 | 0.0058 | 0.0006 | 0.004 | 0.0014 | 390 | YVHSAVLAG | 269 | YVHSAVLAG | 269 | YVHSAVLAG |
| 04:09 |  | 0.0002 | 0 | 0 | – | – | – | – | – | – |
| 04:10 | 0.001 | 0.0006 | 0.005 | 0.0037 | – | – | – | – | – | – |
| 04:11 | 0 | 0.0008 | 0.024 | 0 | – | – | – | – | – | – |
| 04:14 |  | 0 | 3E-04 | 0 | 269 | YVHSAVLAG | – | – | – | – |
| 04:17 |  | 0 | 5E-04 | 0 | 269 | YVHSAVLAG | – | – | – | – |
| 04:18 |  | 0 | 3E-04 | 0 | 358 | MIGFQVLLS | 358 | MIGFQVLLS | 358 | MIGFQVLLS |
| 04:50 | 0 |  |  |  | – | – | – | – | – | – |
| 07:01 | 0.126 | 0.0977 | 0.105 | 0.082 | – | – | – | – | 267 | MTYVHSAVL |
| 07:03 |  | 0 | 0 | 0.0003 | – | – | – | – | 267 | MTYVHSAVL |
| 08:01 | 0.0279 | 0.0046 | 0.008 | 0.0045 | – | – | – | – | – | – |
| 08:02 | 0.0029 | 0.001 | 0.073 | 0.013 | 269 | YVHSAVLAG | – | – | – | – |
| 08:03 | 0.001 | 0.0004 | 0.001 | 0.0518 | – | – | – | – | – | – |
| 08:04 | 0.0029 | 0.0505 | 0.011 | 0 | – | – | – | – | – | – |
| 08:05 |  | 0 | 0 | 0.0003 | – | – | – | – | – | – |
| 08:06 |  | 0.0052 | 0.001 | 0 | – | – | – | – | – | – |
| 08:09 |  | 0 | 0 | 0.0023 | 269 | YVHSAVLAG | – | – | – | – |
| 08:10 | 0.001 | 0 | 3E-04 | 0 | – | – | – | – | – | – |
| 08:11 | 0.001 | 0.001 | 0 | 0 | – | – | – | – | – | – |
| 09:01 | 0.0106 | 0.0316 | 0.01 | 0.1018 | 269 | YVHSAVLAG | – | – | – | – |
| 10:01 | 0.0077 | 0.0185 | 0.015 | 0.0311 | 269 | YVHSAVLAG | – | – | – | – |
| 11:01 | 0.049 | 0.0871 | 0.042 | 0.0512 | – | – | – | – | – | – |
| 11:02 | 0.0039 | 0.0391 | 0.013 | 0 | – | – | – | – | – | – |
| 11:03 | 0.0077 | 0.0006 | 0.006 | 0 | – | – | – | – | – | – |
| 11:04 | 0.0231 | 0.0056 | 0.026 | 0.0065 | – | – | – | – | – | – |
| 11:06 |  | 0 | 0 | 0.0031 | – | – | – | – | – | – |
| 11:08 |  | 0 | 0 | 0.0003 | – | – | – | – | – | – |
| 11:09 |  | 0 | 0 | 0 | – | – | – | – | – | – |
| 11:10 |  | 0.0017 | 3E-04 | 0 | – | – | – | – | – | – |
| 11:11 |  | 0 | 0 | 0.0009 | – | – | – | – | – | – |
| 11:12 | 0.001 |  |  |  | – | – | – | – | – | – |
| 11:15 |  | 0 | 3E-04 | 0 | – | – | – | – | – | – |
| 11:17 |  | 0.0002 | 0 | 0 | – | – | – | – | – | – |
| 11:39 |  | 0 | 0 | 0 | – | – | – | – | – | – |
| 12:01 | 0.0173 | 0.0395 | 0.012 | 0.0289 | 303 | LISVGGAKY | 303 | LISVGGAKY | – | – |
| 12:02 |  | 0.0027 | 8E-04 | 0.0741 | 303 | LISVGGAKY | 303 | LISVGGAKY | – | – |
| 12:08 |  | 0 | 0 | 0.0003 | 303 | LISVGGAKY | 303 | LISVGGAKY | – | – |
| 13:01 | 0.0558 | 0.0555 | 0.042 | 0.0238 | – | – | – | – | – | – |
| 13:02 | 0.0519 | 0.0645 | 0.039 | 0.0362 | – | – | – | – | 165 | YHMNLRHFY |
| 13:03 | 0.0125 | 0.037 | 0.015 | 0.0003 | – | – | – | – | – | – |
| 13:04 | 0 | 0.0131 | 0.006 | 0 | – | – | – | – | – | – |
| 13:05 | 0.0029 | 0.0004 | 0.003 | 0 | – | – | – | – | – | – |
| 13:06 |  | 0 | 3E-04 | 0 | – | – | – | – | – | – |
| 13:09 |  | 0 | 3E-04 | 0 | – | – | – | – | – | – |
| 13:10 | 0 |  |  |  | – | – | – | – | – | – |
| 13:11 |  | 0.0002 | 3E-04 | 0 | – | – | – | – | – | – |
| 13:12 |  | 0 | 0 | 0.0042 | – | – | – | – | – | – |
| 13:16 |  | 0.0004 | 0 | 0 | – | – | – | – | 165 | YHMNLRHFY |
| 13:20 |  | 0.0002 | 0 | 0 | – | – | – | – | – | – |
| 13:31 |  | 0.0006 | 0 | 0 | – | – | – | – | 165 | YHMNLRHFY |
| 13:36 |  | 0.0002 | 0 | 0 | – | – | – | – | 165 | YHMNLRHFY |
| 13:40 |  | 0 | 3E-04 | 0 | – | – | – | – | – | – |
| 13:50 |  | 0 | 0 | 0.0003 | – | – | – | – | – | – |
| 14:01 | 0.0192 | 0.0214 | 0.014 | 0.024 | – | – | – | – | – | – |
| 14:02 | 0 | 0.0006 | 0.024 | 0.0003 | – | – | – | – | – | – |
| 14:03 |  | 0 | 0 | 0.0037 | – | – | – | – | – | – |
| 14:04 |  | 0.0004 | 3E-04 | 0.0204 | – | – | – | – | – | – |
| 14:05 |  | 0 | 3E-04 | 0.0175 | – | – | – | – | – | – |
| 14:06 |  | 0 | 0.026 | 0.0028 | – | – | – | – | – | – |
| 14:07 | 0.0019 | 0 | 0.001 | 0.0026 | – | – | – | – | – | – |
| 14:08 |  | 0 | 0 | 0.0003 | – | – | – | – | – | – |
| 14:12 |  | 0 | 0 | 0.0003 | – | – | – | – | – | – |
| 14:18 |  | 0 | 0 | 0.0014 | – | – | – | – | – | – |
| 14:19 |  | 0 | 0 | 0.0006 | – | – | – | – | – | – |
| 14:22 |  | 0 | 0 | 0.0003 | – | – | – | – | – | – |
| 14:25 |  | 0 | 0 | 0.0003 | – | – | – | – | – | – |
| 15:01 | 0.1394 | 0.0293 | 0.067 | 0.0792 | 98 | LSQFPSGKV | 98 | LSQFPSGKV | 390 | LKIWKAPHV |
| 15:02 | 0.0077 | 0.0017 | 0.013 | 0.0809 | 98, 239 | LSQFPSGKV, FNTYYAVAV | 98, 239 | LSQFPSGKV, FNTYYAVAV | 239, 390 | FNTYYALAV, LKIWKAPHV |
| 15:03 | 0.001 | 0.1175 | 0.011 | 0.0006 | 98 | LSQFPSGKV | 98 | LSQFPSGKV | 390 | LKIWKAPHV |
| 15:04 |  | 0 | 3E-04 | 0.0006 | 98, 390 | LSQFPSGKV, LKIWKAPHE | 98, 390 | LSQFPSGKV, LKIWKAPHE | 390 | LKIWKAPHV |
| 15:06 |  | 0 | 0 | 0.004 | 98 | LSQFPSGKV | 98 | LSQFPSGKV | 390 | LKIWKAPHV |
| 15:07 |  | 0 | 0 | 0.0003 | 98 | LSQFPSGKV | 98 | LSQFPSGKV | 390 | LKIWKAPHV |
| 15:10 | 0.001 |  |  |  | 98, 390 | LSQFPSGKV, LKIWKAPHE | 98, 390 | LSQFPSGKV, LKIWKAPHE | 390 | LKIWKAPHV |
| 15:11 | 0.001 |  |  |  | 239 | FNTYYAVAV | 239 | FNTYYAVAV | 390 | LKIWKAPHV |
| 15:14 |  | 0 | 0 | 0.0003 | 98, 239 | LSQFPSGKV, FNTYYAVAV | 98, 239 | LSQFPSGKV, FNTYYAVAV | 239, 390 | FNTYYALAV, LKIWKAPHV |
| 16:01 | 0.0067 | 0.001 | 0.008 | 0.0003 | – | – | – | – | – | – |
| 16:02 | 0 | 0.0154 | 0.021 | 0.0195 | – | – | – | – | – | – |

*Note*: Allele Frequency: Total number of allele copies in the population sample (alleles/2n) in decimal format. Blank indicates not investigated in Allele Frequency Net Database (AFND).
