# Supplementary material for: Prediction of immunogenicity of Rh antigens using in silico analysis of binding to human leukocyte antigen peptide, Basic/Translational Research
Source: PLoS One. 2025 Oct 27;20(10):e0334851. doi: 10.1371/journal.pone.0334851 (PMC12558515; doi:10.1371/journal.pone.0334851)
Supplement: S3 Table — (DOCX) [file pone.0334851.s005.docx]

**S3 Table. HLA-DRB1 gene frequencies and Rh hotspot positions in Korean and Japanese populations.**

| Serology | Allele | Korean* (n = 474) | Japanese^#^ (n = 371) | *RHD*01.01 / RHD*01W.2 / RHD*01W.3* | | *RHD*01W.1* | | *RHCE*01* | |
| --- | --- | --- | --- | --- | --- | --- | --- | --- | --- |
|  |  |  |  | Hotspot amino acid start position | Core amino acids | Hotspot amino acid start position | Core amino acids | Hotspot amino acid position | Core amino acids |
| DR1 | 0101 | 7.4 | 6.5 | - | - | - | - | - | - |
| DR17 (DR3) | 0301 | 2.2 | 0 | 125 | ISVDAVLGK | 125 | ISVDAVLGK | 125 | ISVDAVLGK |
| DR4 | 0401 | 0.4 | 0.7 | 269 | YVHSAVLAG | - | - | 269 | YVHSAVLAG |
|  | 0403 | 2.6 | 4 | - | - | - | - | - | - |
|  | 0404 | 0.9 | 0.1 | - | - | - | - | - | - |
|  | 0405 | 8.5 | 11.5 | - | - | - | - | - | - |
|  | 0406 | 5.3 | 3.5 | - | - | - | - | - | - |
|  | 0407 | 0.3 | 0.9 | 269 | YVHSAVLAG | 269 | YGHSAVLAG | 269 | YVHSAVLAG |
|  | 0408 |  |  | 269 | YVHSAVLAG | 269 | YVHSAVLAG | 269 | YVHSAVLAG |
|  | 0410 | 0.8 | 1.8 | - | - | - | - | - | - |
| DR7 | **0701** | 7.3 | 0.3 | - | - | - | - | 267 | MTYVHSAVL |
| DR8 | 0802 | 3.2 | 4 | 269 | YVHSAVLAG | - | - | - | - |
|  | 0803 | 7.2 | 8.1 | - | - | - | - | - | - |
| DR9 | 0901 | 10.4 | 12.4 | 269 | YVHSAVLAG | - | - | - | - |
| DR10 | 1001 | 1.5 | 0.9 | 269 | YVHSAVLAG | - | - | - | - |
| DR11 | 1101 | 3.2 | 3.4 | - | - | - | - | - | - |
| DR12 | 1201 | 5.1 | 3.8 | 303 | LISVGGAKY | 303 | LISVGGAKY | - | - |
|  | 1202 | 3.6 | 1.5 | 303 | LISVGGAKY | 303 | LISVGGAKY | - | - |
| DR13 | 1301 | 1.9 | 0.7 | - | - | - | - | - | - |
|  | 1302 | 7.8 | 7.7 | - | - | - | - | 165 | YHMNLRHFY |
|  | 1307 |  |  | - | - | - | - | - | - |
| DR14 | 1401 | 3.3 | 4.2 | - | - | - | - | - | - |
|  | 1402 | 0.2 | 0 | - | - | - | - | - | - |
|  | 1403 | 1.2 | 1.5 | - | - | - | - | - | - |
|  | 1405 | 3.7 | 1.1 | - | - | - | - | - | - |
|  | 1406 | 0.2 | 1.8 | - | - | - | - | - | - |
|  | 1407 | 0.2 | 0.3 | - | - | - | - | - | - |
|  | 1412 | 0.1 | 0.1 | - | - | - | - | - | - |
| DR15 | 1501 | 8 | 8.5 | 98 | LSQFPSGKV | 98 | LSQFPSGKV | 390 | LKIWKAPHV |
|  | **1502** | 3 | 10 | 98, 239 | LSQFPSGKV, FNTYYAVAV | 98, 239 | LSQFPSGKV, FNTYYAVAV | 239, 390 | FNTYYALAV, LKIWKAPHV |
| DR16 | 1602 | 0.5 | 0.9 | - | - | - | - | - | - |

*Note*: HLA alleles with significantly different gene frequencies between Koreans and Japanese populations are underlined (*P* < 0.001) or in bold (*P* < 1×10^-6^).

*Koreans: from reference (1)

^#^Japanese: from reference (2)

**References**

1. Chung HY, Yoon JA, Han BY, Song EY, Park MH. Allelic and haplotypic diversity of HLA-A, -B, -C, and -DRB1 genes in Koreans defined by high-resolution DNA typing. Korean J Lab Med. 2010;30:685-696.

2. Saito S, Ota S, Yamada E, Inoko H, Ota M. Allele frequencies and haplotypic associations defined by allelic DNA typing at HLA class I and class II loci in the Japanese population. Tissue Antigens. 2000;56:522-529.
